# Gastric Cancer Pre-Stage Detection and Early Diagnosis of Gastritis Using Serum Protein Signatures

**DOI:** 10.3390/molecules27092857

**Published:** 2022-04-30

**Authors:** Shahid Aziz, Faisal Rasheed, Rabaab Zahra, Simone König

**Affiliations:** 1 BreathMAT Lab, Pakistan Institute of Nuclear Science and Technology (PINSTEC), Islamabad 44000, Pakistan; saziz@bs.qau.edu.pk (S.A.); frohpl@gmail.com (F.R.); 2 Department of Microbiology, Faculty of Biological Sciences, Quaid-i-Azam University, Islamabad 45320, Pakistan; rzahra@qau.edu.pk; 3 IZKF Core Unit Proteomics, University of Münster, 48149 Münster, Germany

**Keywords:** *Helicobacter pylori*, gastric cancer, ulcer, gastritis, proteomics

## Abstract

Background: A gastric cancer (GC) diagnosis relies on histopathology. Endoscopy rates are increasing. *Helicobacter pylori* infection is a major GC risk factor. In an effort to elucidate abundant blood biomarkers, and potentially reduce the number of diagnostic surgical interventions, we investigated sera and biopsies from a cohort of 219 *H. pylori* positive and negative patients diagnosed with GC, gastritis, and ulcers. This allowed the comparative investigation of the different gastroduodenal diseases, and the exclusion of protein changes resulting from bacterial infection or inflammation of the gastric mucosa when searching for GC-dependent proteins. Methods: High-definition mass spectrometry-based expression analysis of tryptically digested proteins was performed, followed by multivariate statistical and network analyses for the different disease groups, with respect to *H. pylori* infection status. Significantly regulated proteins differing more than two-fold between groups were shortlisted, and their role in gastritis and GC discussed. Results: We present data of comparative protein analyses of biopsies and sera from patients suffering from mild to advanced gastritis, ulcers, and early to advanced GC, in conjunction with a wealth of metadata, clinical information, histopathological evaluation, and *H. pylori* infection status. We used samples from pre-malignant stages to extract prospective serum markers for early-stage GC, and present a 29-protein marker panel containing, amongst others, integrin β-6 and glutathione peroxidase. Furthermore, ten serum markers specific for advanced GC, independent of *H. pylori* infection, are provided. They include CRP, protein S100A9, and kallistatin. The majority of these proteins were previously discussed in the context of cancer or GC. In addition, we detected hypoalbuminemia and increased fibrinogen serum levels in gastritis. Conclusion: Two protein panels were suggested for the development of multiplex tests for GC serum diagnostics. For most of the elements contained in these panels, individual commercial tests are available. Thus, we envision the design of multi-protein assays, incorporating several to all of the panel members, in order to gain a level of specificity that cannot be achieved by testing a single protein alone. As their development and validation will take time, gastritis diagnosis based on the fibrinogen to albumin serum ratio may be a quick way forward. Its determination at the primary/secondary care level for early diagnosis could significantly reduce the number of referrals to endoscopy. Preventive measures are in high demand. The protein marker panels presented in this work will contribute to improved GC diagnostics, once they have been transferred from a research result to a practical tool.

## 1. Introduction

Stomach cancer will kill ~11,090 patients (60% male) in the US in 2022 [1]. *Helicobacter pylori* is a major carcinogen for non-cardia gastric cancer (GC) [2]. GC is commonly diagnosed at an advanced stage, and is one of hardest to treat among all cancers, with an overall survival rate of 10–12 months [3]. The gold standard for GC diagnosis is the histopathological investigation of biopsies. The high need for endoscopy not only constitutes considerable strain for the patient, but is also a large financial burden for the health care system [4]. Although many prospective biomarkers are reported, only some are used in clinical practice (e.g., carbohydrate antigen and carcinoembryonic antigen), and they are not specific for GC [5]. Modern omics methods have advanced biomarker research in recent years leading, for instance, to the Cancer Genome Atlas [6], and a region-resolved mucosa proteome of the human stomach [7]. Different analytical methodologies, including mass spectrometry (MS), identified, mostly from biopsies, proteins with diagnostic potential for GC [5,8,9]. However, their validation is time consuming. Moreover, the influence of secondary factors such as *H. pylori* infection [10], or the location of the tumor within the stomach, was often not studied.

In this investigation, we assembled a patient cohort of both *H. pylori* positive (HPp) and negative (HPn) probands diagnosed with GC, gastritis, and ulcers. This allowed the comparative investigation of the different gastroduodenal diseases, and the exclusion of protein changes resulting from bacterial infection or inflammation of the gastric mucosa, when searching for GC-dependent proteins. Moreover, we looked at the differences between normal mucosa and diseased sites, as well as the stomach region (antrum, corpus). Ultimately aiming for abundant GC blood biomarkers, we studied both biopsies and serum of these patients, using proteomics methods based on high-resolution MS with ion mobility separation followed by multivariate statistics and network analysis. The study was conducted in conjunction with the collection of a wealth of metadata concerning GC risk factors and clinical parameters (to be published elsewhere [11]).

## 2. Results

**Figure 1:**
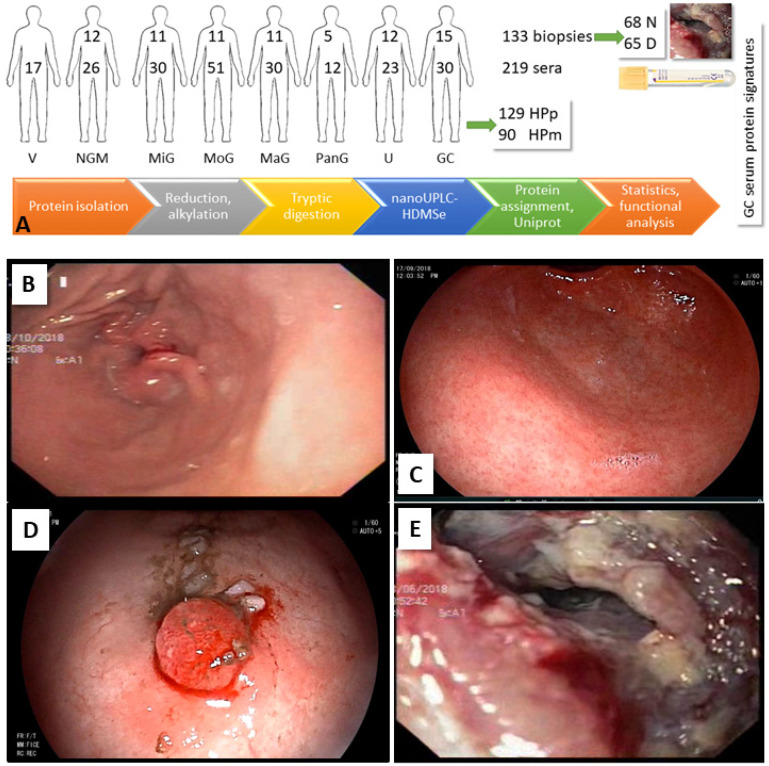
Experimental design and workflow for the elucidation of a GC serum protein marker profile. (**A**) Biopsies (133, 68 N site, 65 D site) and sera (219) were collected. More than half of the probands were *H. pylori* positive (59%). Proteins were isolated, trypsinized, and subjected to data-independent MS. Proteins were assigned, and the differences between disease groups were determined considering gender, infection status, and biopsy site, followed by functional classification and network analysis. (**B**–**E**) HPp patients with different gastroduodenal clinical manifestations. (**B**) NGM (no inflammation in antrum), (**C**) severe antral gastritis, (**D**) polyp (1st stage GC), (**E**) GC (differentiated adenocarcinoma).

We investigated the protein profiles of gastric biopsies and serum from 75 and 219 probands, respectively. The experimental design is shown in Figure 1A. Data, results, analysis information, and references are available in the Supplement and Supplementary Excel Files “tissue_analysis”, “tissue”, “serum_analysis”, and “serum”. 

### 2.1. Patients and Samples

Symptomatic patients with upper gastroduodenal problems, including acid reflux, abdominal pain, heartburn, vomiting, and bloating attending the Center for Liver & Digestive Diseases, Holy Family Hospital, Rawalpindi, for gastroduodenal endoscopy were enrolled (n = 75, Table 1). 

The patients were divided into groups, according to the gastroduodenal clinical manifestations and histopathological evaluation (normal mucosa—NGM, mild gastritis—MiG; moderate gastritis—MoG; marked gastritis—MaG; pan gastritis—PanG; ulceration—U (gastric ulcer, duodenal ulcer); GC (first and advanced stage)). Forrest classification was used to evaluate ulcer-related upper gastroduodenal bleeding, in order to identify patients at increased risk of bleeding and re-bleeding [12]. Patients with a history of use of proton pump inhibitors, H_2_ receptor antagonists, and antibiotics taken four weeks prior to endoscopy; those with active bleeding; corrosive intake; a history of gastric surgery; and gastropathy were excluded, as well as patients taking anticancer drugs. Slightly more males participated in the study, and 60% of the GC patients were male. The NGM cohort mainly consists of probands younger than 46 years of age, while the age of GC patients is distributed from 19 to 70 years. MiG is diagnosed more often in females (64%), MaG (73%), and ulcers in males (67%); this is likely because of the tendency of males with symptoms to see a doctor later than when females do. Gastritis patients, with the exception of those with PanG, are mostly comparatively young (73% < 46 years) in contrast to U patients (75% > 45 years). For the diagnosis of *H. pylori* infection, ^13^C urea breath test was performed as described previously [13], and the presence of *H. pylori* within gastric biopsy specimens was evaluated by routine histopathological methods (for details, see Supplement). Most of the study participants (70%) were *H. pylori* positive. Gastric biopsy specimens were, if possible, collected from normal (N) and adjacent diseased (D) parts of the stomach antrum (with the exception of three NGM samples from corpus) during gastroduodenal endoscopy. Histopathological grades, including *H. pylori* load; degree of neutrophil and mononuclear cell infiltration; and the presence of atypia, atrophy, intestinal metaplasia, and dysplasia were scored based on the updated Sydney System [14]. Additionally, gastric biopsies were assessed according to the Operative Link for Gastritis Assessment (OLGA) scoring system, and the Lauren and WHO classification systems [15]. Serum was obtained from 219 probands, which included the above-mentioned patients, as well as healthy volunteers (V, Table 1). Metadata were collected using a pre-examined Likert scale questionnaire [16].

### 2.2. Biopsy Analysis

The exemplary clinical manifestations in HPp patients are shown in Figure 1. Histopathology results for GC biopsies are given in Supplementary Table S1. Seven patients were diagnosed with adenocarcinoma, and five with signet ring cell carcinoma. All specimens were the diffused type according to the Lauren classification, but divided into tubular (all adenocarcinomas) and poorly cohesive forms following WHO classification. Two of the patients were special cases (isolated dextrogastria [17], gastric metastasis from primary invasive lobular breast carcinoma [18]). 

**Figure 2:**
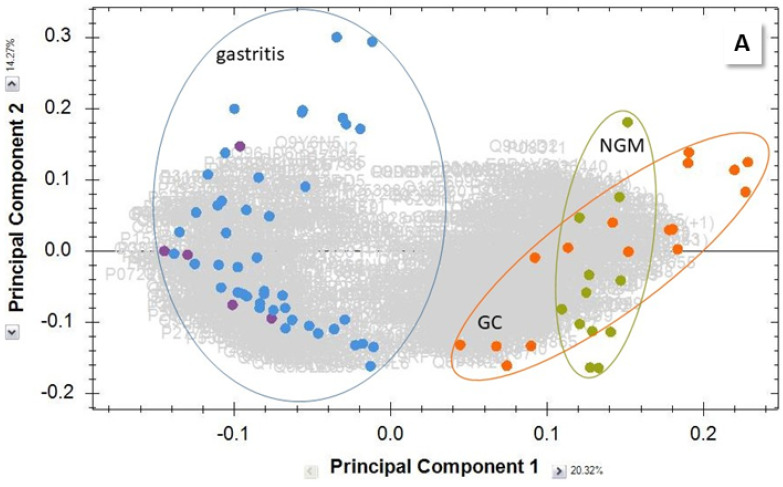

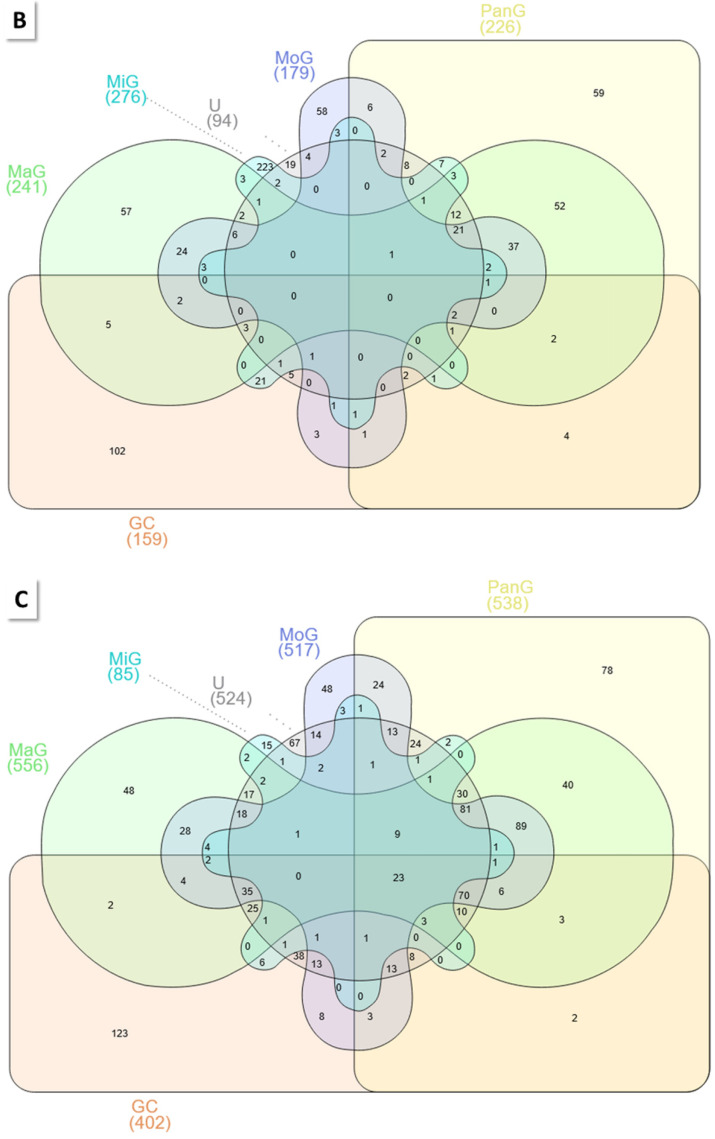
Comparative biopsy analysis. (**A**) PCA of combined MoG and MaG (blue) and PanG (purple) shortlisted proteins, compared to NGM (green) and GC (orange), demonstrate the separation of gastritis protein profiles. (**B**) Venn diagrams showing the number of shortlisted proteins common among the different biopsy groups. Proteins with (**B**) higher and (**C**) lower concentration than in NGM.

Biopsies were homogenized, the proteins extracted, reduced and alkylated, then tryptically digested, and subjected to separation by reversed-phase liquid chromatography (LC) and detection by MS (see Experimental Section). Individual sample group comparisons of the 2823 quantifiable proteins were performed and the results meeting the filter criteria (up to 1094 proteins per pair) were analyzed further. In the cancer group, six runs show peak patterns in their chromatograms resulting from contamination, and had to be removed from the project. These biopsies had been stored in RNAlater solution, a commercial product for RNA analysis, which introduced an undesired matrix. Global principal components analysis (PCA) of all data separated severe gastritis cases from GC and normal tissue (Figure 2A), while MiG samples clustered with NGM, GC, and ulcers did not form a separate group (detailed PCA, Supplementary Figure S1). The Venn diagrams in Figure 2B,C visualize the shared shortlisted of up- or downregulated proteins for the different gastric diseases, and show that 102 and 123 proteins, respectively, were specific for GC.

#### 2.2.1. GC vs. NGM

**Figure 3:**
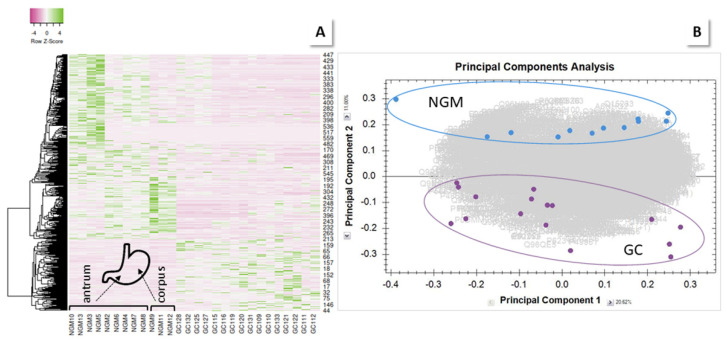

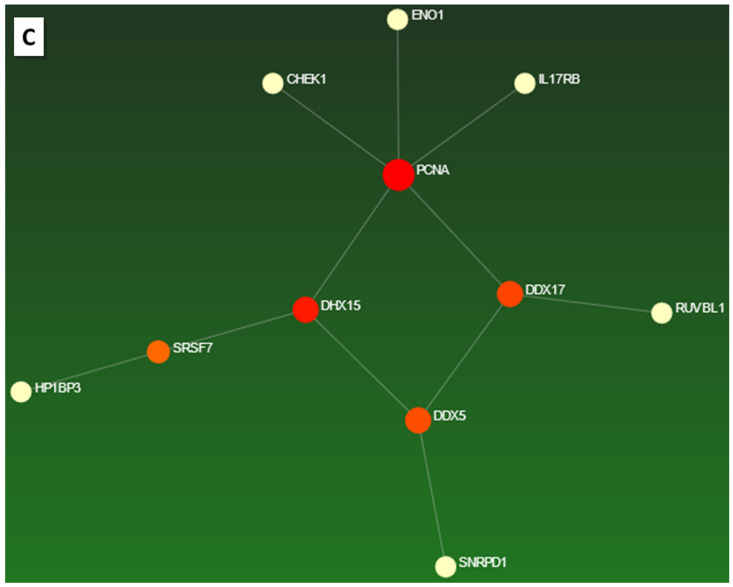

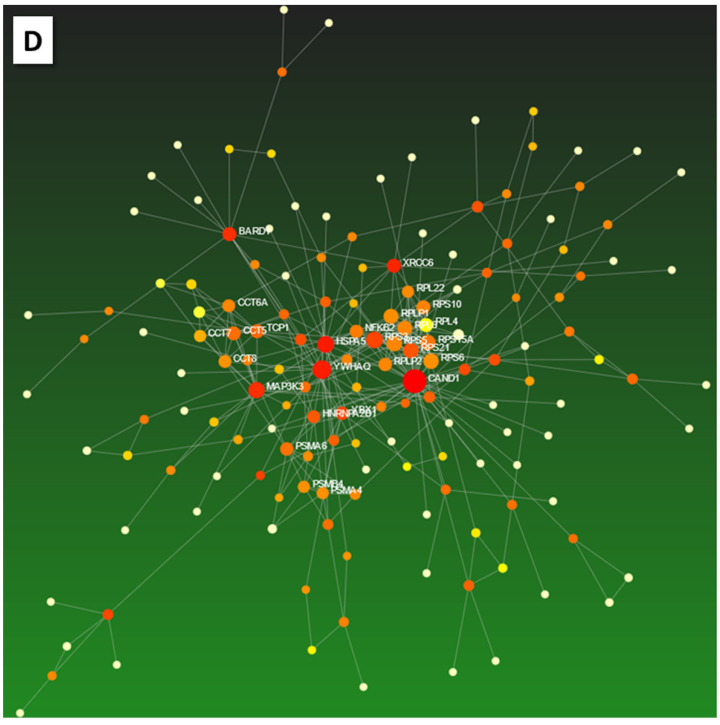
Comparison of GC vs. NGM shortlisted proteins. (**A**) Heatmap (Spearman rank correlation). (**B**) PCA. (**C**,**D**) Zero-order networks of (**C**) up-, and (**D**) downregulated proteins. For details, see Supplementary Figures S6 and S7.

As the heatmap and PCA in Figure 3A,B illustrate, both sample groups are clearly separated. However, the NMG samples cluster in at least two clearly discernible groups (Figure 3A). This effect is even more pronounced when mapping only the HPpD samples (Supplementary Figure S2) and it resulted from the origin of the biopsies, three of which were obtained from corpus, because of bleeding in antrum, while all other samples were collected from antrum. Histopathological evaluation do not show such drastic differences, irrespective of biopsy site. It is known that stomach regions exhibit differing protein profiles depending on their task, in particular, the presence of specific glands [7]. Therefore, a separate dedicated comparison of antrum samples was performed. 

Inter-individual differences in the protein profiles are larger than those between the N and D biopsy sites. This is evident in the heatmaps (Supplementary Figures S3 and S4; NGM vs. GC: all data, HPp, HPpD; GC), where the N and D samples, despite subtle differences, cluster together for each patient, rather than separating in person-independent N and D blocks. 

When the shortlisted, upregulated proteins in GC were investigated by network enrichment analysis, as expected [19], many proteins from metabolic pathways were detected, including α-enolase (ENO1), previously earmarked as a cancer biomarker [20] (Supplementary Figure S5). The zero-order protein network of these proteins is shown in Figure 3C, and features proliferating cell nuclear antigen (PCNA) in addition to RNA helicases, which were proposed as oncotherapeutic targets [21] (network visualizations, Supplementary Figures S6 and S7). The network of proteins downregulated in GC also contains many components of metabolic pathways (Figure 3D, Supplementary Figures S8–S10). Classification analysis shows that the molecular functions of the up- and downregulated proteins differ in transducer activity, as a result of highly expressed in GC: receptor-type tyrosine-protein kinase (FLT3); interleukin-17 receptor B (IL17RB); lithostathine-1-β (REG1B); vascular endothelial growth factor receptor 1 (FLT1); and glycine receptor subunit α-3 (GLRA3) (Supplementary Figure S11), all of which are associated with cancer, and specifically GC [22,23,24,25,26].

##### HPpD

The number of shortlisted regulated proteins is reduced (from 561 to 457) and, as expected, not all proteins are shared with those of the global analysis (94 up-, 278 downregulated in GC; Venn diagram, Supplementary Figure S12). Interestingly, ENO1, among others, is not shared and, thus, seems to be acting independent of *H. pylori.* Network analyses and functional classification are supplied in Supplementary Figures S13–S17. 

##### GC N vs. D Sites

The protein profiles of normal mucosa and cancerous biopsies differ by comparatively few (39) proteins (for upregulated proteins in GC, see Supplementary Table S2). The PCA and the exemplary expression profile of the abundant villin-1 (VIL1) [27] are shown in Supplementary Figure S18. VIL1 and heat shock 70 kDa protein 4 (HSPA4) varied in concentration between D and N biopsy sites (Venn diagram GC vs. NGM, D vs. N, Supplementary Figure S19). HSPA4 is called the “evil chaperone” in the context of delayed ulcer healing [28], and is predictive of poor GC diagnosis [29]. Fatty acid biosynthesis, metabolism, and elongation processes are, not surprisingly [19], increased in D sites, while members of the cancer-associated ErbB signaling pathway [30] and proteoglycans [31] are reduced (network, functional classification, Supplementary Figures S20 and S21). 

##### Gender

No strong correlation is observed in the heatmap (Supplementary Figure S4), but protein factors are still detected, which separate the groups when comparing the samples of D sites from males and females (n = 4 each, PCA, Supplementary Figure S22). Among the 224 differently regulated proteins that are more abundant in male D sites are tricarboxylate transport protein (SLC25A1) [32], proteasome β-4 subunit (PSMB4) [33], and type II cytoskeletal 2 oral keratin (KRT76) [34], all with known roles in cancer (expression profiles, Supplementary Figure S22). Metabolic processes seem to be more active in males than in females, and more pathways are involved (network and functional analyses, Supplementary Figures S23–S28).

##### 1st and Advanced Stage GC

Four samples of advanced and three of 1st stage GC were compared. The shortlisted proteins (283) separate the groups in PCA (Supplementary Figure S29). At the top of the list for the proteins upregulated in advanced GC are α-actin cardiac muscle 1 (ACTC1), which is noted in prostate cancer [35], and complement C4. A relationship between the complement system, and tumor prognosis and immune infiltration in colon cancer and GC is detected [36]. Signaling pathways including p53, Wnt, and VEGF are involved (Supplementary Figures S30–S37). The zero-order network of proteins upregulated in advanced GC centers on cullin-3 (CUL3), which is part of the ubiquitin–proteasome system controlling many physiological processes, including cancer [37]. 

##### Antrum Samples

The entire analysis was repeated for the NGM antrum biopsies (PCA NGM vs. GC, Supplementary Figure S38). As the Venn diagram in Supplementary Figure S39 shows, only 106 of the in-GC upregulated proteins are shared with the analysis for antrum and corpus biopsies. For HPpD sites, 84 proteins are shared (PCA, Venn, Supplementary Figures S40 and S41; functional classification, network, Supplementary Figures S42–S49). Independently of *H. pylori* infection, 89 proteins are more abundant in GC than in NGM (top hits, Supplementary Table S3; Venn, network, Supplementary Figures S50 and S51). 

#### 2.2.2. Other Group Comparisons

PCA demonstrates group separation, and the results are shown in Supplementary Figures S52 and S53. In order to isolate the proteins that are dominant in GC compared to other gastroduodenal afflictions, Venn analysis was performed (Supplementary Figures S54 and S55). Proteins that are both more abundant in HPpD GC vs. U and combined MaG/MoG (111), and those that show the highest values in GC in general in the entire dataset, were screened to elucidate the most prominent GC-related proteins. A list of these 34 proteins is given in Supplementary File “analysis” including references, because all of the proteins were already discussed in the context of cancer. The analysis was repeated for antrum biopsy specimens. Independent of *H. pylori* infection, 56 proteins are highly expressed in GC (Supplementary Table S3). They represent a cohort of target proteins for further GC marker research. Their zero-order network (Supplementary Figure S51) is similar to the one shown in Figure 3, for the GC/NGM comparison featuring the CHEK1- (checkpoint kinase 1 Chk1)PCNA-DDX17 (probable ATP-dependent RNA helicase 17)DDX5-SNRPD1 (small nuclear ribonucleoprotein Sm D1) axis, but missing the connections to DDX15 and ENO1. CHK1 is central in coordinating the response to DNA damage and, thus, of great interest in oncology and the development of cancer therapeutics [38]. The splicing factor SNRPD1 is associated with alternative splicing events in lung, breast, and skin cancer [39]. RNA helicases also have known roles in cancer [32]; in particular, DDX5 promotes GC cell proliferation [40], and DDX17 hepatocellular carcinoma progression via inhibiting Klf4 transcriptional activity [41].

### 2.3. Comparative Serum Analysis

**Figure 4:**
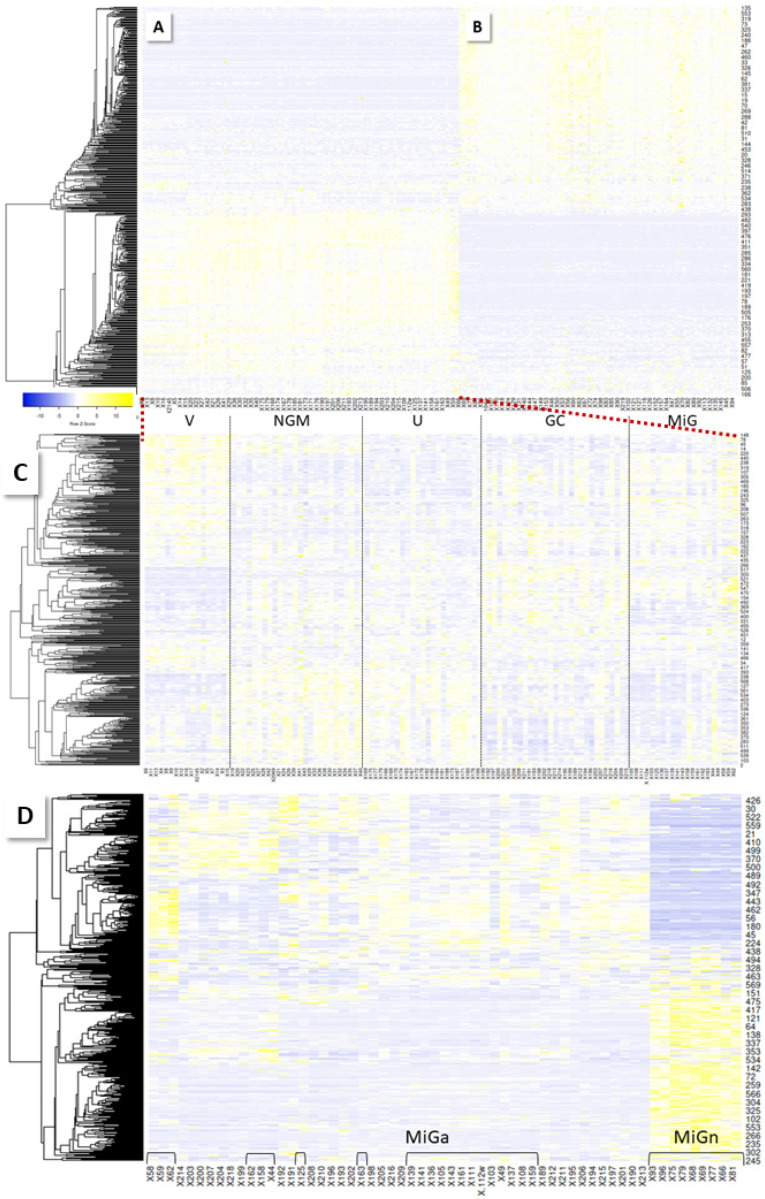
Heatmaps of serum sample results (Spearman rank). A clear distinction in the protein profiles between gastritis samples (**B**) and all other groups (**A**) is detected. Most interestingly, a part of the MiG cases (called MiGa below) cluster with GC samples. (**C**) Zoom-in. (**D**) Heatmap of MiG and GC samples clustered by rows and columns. Brackets indicate MiG samples; all other samples are from GC patients. Regular MiG (MiGn) is set apart from GC/MiGa.

Analogous to biopsy proteins, serum proteins were tryptically digested and analyzed, using LC-MS (see Experimental Section). A total of 884 proteins were quantified and subjected to sample group comparisons. The PCA of all shortlisted data visualizes the complete separation of the NGM and V groups (Supplementary Figure S56). GC and U are well distinguished from V; gastritis samples are more spread out (PCAs V and NGM, HPp/m, vs. GC, U and gastritis, Supplementary Figures S57–S60). The heatmap of the entire shortlisted data (Figure 4) illustrates the surprising finding of a sharp distinction between gastritis samples and all other groups, which is not observed in the same way in biopsies. 

#### 2.3.1. MiG

Even more interesting is the finding that more than half of the MiG cases sided with the GC cases in the heatmap (Figure 4C,D). Normal MiG (MiGn) samples are clearly differentiated from atypical MiG (MiGa) cases, which are distributed among the GC samples. Those turn out, gender independently, to all be from HPp patients, plus some HPn patients (36%), while the other MiG cases are all from HPn patients. Moreover, it is the cases where histopathology examination has detected either gastric atypia (6 of 21 cases), or atrophic gastritis (12/21). In one case, no histopathology is available, and two others appear to be normal. Normal mucosa may be detected among diseased biopsies, by chance, when several samples need to be collected during one endoscopy procedure. The PCA (Supplementary Figure S59) demonstrates group separation for MiGn vs. V and NGM, as well as for MiGa vs. V, but there is a slight overlap for the MiGa vs. NGM samples. 

Given that both gastric atypia [42] and atrophic gastritis [43] potentially develop into malignant stages, this result is of great interest, as it implies that GC can be predicted from serum at an early stage. As the string networks of the upregulated proteins in MiGn and MiGa (Supplementary Figures S61 and S62) show, albumin/apolipoprotein-centered interactions in MiGn lose importance in MiGa and give way to, among others, members of the glutathione antioxidant system (glutathione S-transferase theta-1 (GSTT1), glutathione peroxidase 3 (GPX39)); these disturbances are implicated in tumor initiation and progression [44]. In fact, these enzymes increase in red blood cells of GC patients [45]. Also, the protein S100A9 is observed, which is known to promote invasion and migration through the p38 mitogen-activated, protein kinase-dependent NF-κB activation in gastric cancer cells [46]. The proteins differing between the upregulated proteins in MiGa and MiGn vs. NGM were determined (Venn, Supplementary File “serum_analysis”, 64 shared proteins), and a panel of 25 proteins (Table 2), all with potential as markers for the detection of early stage GC in serum, was chosen following manual spectral data evaluation from the 41 proteins characteristic for MiGa (25preGC-panel). These proteins, for the most part, did not belong to the abundant proteins in MiGa serum, but all have a connection to cancer, and even GC (for references, see Supplementary File “serum_analysis”).

Furthermore, six proteins matched between the subsets MiGa (serum) and polyp (biopsies) vs. NGM, including DDX17 and GSTT1, which were already part of the panel. Moreover, immunoglobulin heavy constant γ 4 (IGHG4), protein S100 A10 (S100A10), cAMP-dependent protein kinase inhibitor α (PKIA), and the TBC1 domain family member 25 (TBC1D25) are shared. Immunoglobulins have a critical role of in cancer [47]. S100A10 accelerates aerobic glycolysis and malignant growth by activating the mTOR-signaling pathway in GC [48]. Protein kinase A is involved in the control of a wide variety of cellular processes, and it is implicated in the initiation and progression of many tumors [49]. The least is known about the connection of TBC1D25 and cancer, but the protein is involved in the fusion of autophagosomes with endosomes and lysosomes, and was discussed in a study of mutations altering autophagy selectivity in human cancer [50]. These four proteins were additionally considered for the above preGC marker panel.

#### 2.3.2. Fibrinogen to Albumin Ratio (FAR)

**Figure 5:**
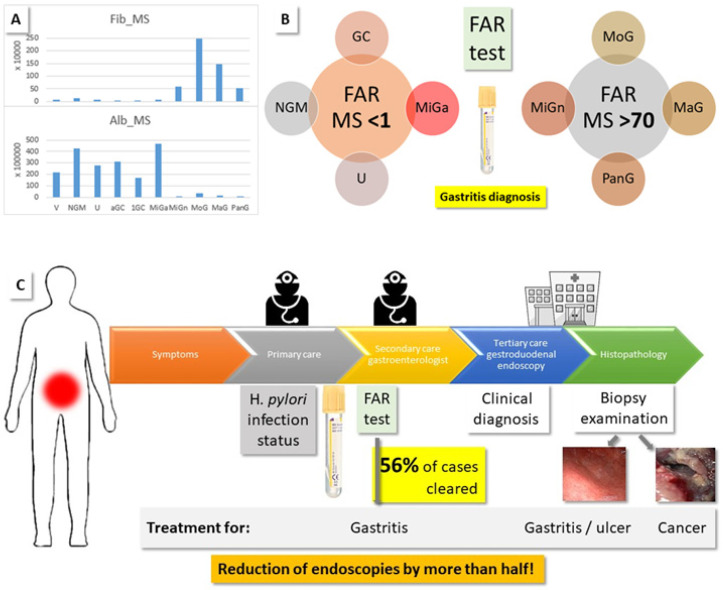
FAR identifies gastritis cases. (**A**) Average MS intensities for serum albumin and fibrinogen detected per patient group. (**B**) FAR-MS significantly distinguished gastritis samples from NGM, GC, U, and MiGa, as a result of hypoalbuminemia and increased serum fibrinogen. (**C**) Current procedure for patients with gastroduodenal symptoms: If the efforts of the primary care physician are not successful, a gastroenterologist is consulted. Persistent cases are referred to endoscopy, which clarifies the clinical diagnosis by histopathological examination of gastric biopsies. We suggest that a FAR lab test, in addition to the determination of the *H. pylori* infection status, can identify the gastritis cases without the need for endoscopy and, thus, reduce the number of medical procedures by about half (as calculated based on the present patient cohort and data).

FAR was assigned a prognostic value in GC patients who had recently received first-line chemotherapy [51]. The quantification in that work was based on routine lab assays, whose results were not directly comparable to our MS intensities, where α/β/γ-fibrinogen chains were measured separately. Nevertheless, we calculated the FAR by adding all intensity values for the fibrinogen chains and dividing this number by the intensity detected for albumin in the different groups (FAR-MS, Table 3). To our surprise, we measure striking differences (*p* < 0.001) between gastritis and all other cases, with FAR-MS values of 74–98 and 0.1–0.3, respectively (Figure 5). Importantly, we noticed the same split of the MiG cases as discussed above: MiGa shows FAR-MS values like the non-gastritis groups, while the values for MiGn are similar to the gastritis cases. NGM, U, advanced GC, and MiGa have higher than normal albumin values, while the fibrinogen values are not changed, except in NGM, where they are double, and in 1st stage GC, where they are about half. In all gastritis cases, except MiGa, albumin decreases up to 30-fold, while the fibrinogen concentration increases almost 50-fold, leading to the huge difference in the FAR-MS values. Based on the MS intensities of serum fibrinogen and albumin, we, therefore, clearly differentiate gastritis cases from other gastroduodenal afflictions, including ulcers and GC. 

The serum albumin level is part of a standard panel of parameters determined in the clinical laboratory, e.g., in liver and kidney disease. Hypoalbuminemia was observed in protein-losing gastropathy [52,53]. *H. pylori* eradication helped in some of these cases, because *H. pylori* infection can lower the serum albumin level [54]. Fibrinogen is an acute-phase protein, i.e., its blood level rises in response to systemic inflammation or trauma by several orders of magnitude. Elevated levels of fibrinogen were suggested as the cause of thrombosis and vascular injury that may occur in inflammation or cancer [55]. In patients with *Helicobacter* associated chronic gastritis, significantly increased plasma fibrinogen levels were observed [56]. We noted differences between HPp and HPn samples (FAR-MS ranges: HPp: 79–87, 0.1–0.3; HPn: 70–116, 0.04–0.3), but they are of no importance in light of the major gap between the values for FAR-MS group 1 (GC, NGM, U, MiGa) and group 2 (gastritis, Figure 5), and were not further investigated.

#### 2.3.3. NGM vs. V

The NGM group was comprised of patients who underwent gastroduodenal endoscopy for symptoms of upper gastroduodenal unrest, but did not have conspicuous mucosa. These sera reflected the unwell state of the patients, e.g., an increased C-reactive protein (CRP), which is a common inflammation marker, compared to V. The PCA shows group separation of the 237 shortlisted proteins differing in the V vs. NGM comparison (Supplementary Figure S57E,F, network Figure S63). *H. pylori* infection led to subtle changes, such as increased keratin presence. Alterations in different cytokeratin expression were previously associated with the weakening of epithelial tight junctions observed in *H. pylori*-infected gastric mucosa [57]. In addition, changes in the complement system are observed, possibly leading up to the complement activation in *H. pylori*-associated gastritis noted by other authors [58]. For functional classification analysis see Supplementary Figures S64 and S65.

Gender-associated differences within the V or NGM groups are negligible (5 and 3 regulated, low abundant proteins, respectively). In addition, few differences are observed between either V or NGM with respect to *H. pylori* infection status (PCAs, Supplementary Figure S57A,C), apart from the observation of abundant immunoglobulin heavy constant mu (IGHM) in both V and NGM HPp samples. A direct relationship of IGHM to *H. pylori* is not evident, but gastric mucosa-associated lymphoid tissue lymphoma B cells are known to express polyreactive, somatically mutated immunoglobulins [47,59]. 

#### 2.3.4. GC vs. V and NGM

The PCA demonstrates group separation (Supplementary Figure S58A–D). Of the 217 differentially regulated proteins, 108 are more abundant in the sera of GC patients than in the V sera. Among them are members of the Wnt and TGF-β signaling pathways, as well as proteins involved in T-cell activation (network, functional classification for HPp/n, Supplementary Figures S66–S69). Elevated levels of inter-α-trypsin inhibitor heavy chain H4 (ITIH4) and α-1B-glycoprotein (A1BG) are detected, both of which were discussed as GC markers, respective as treatment target, in other studies [60,61]. The latter, however, is not as involved when an *H. pylori* infection is present. Instead, other proteins become important, such as frizzled-6 (FZD6), which is the regulator of the Wnt non-canonical pathway in the pathogenesis of different types of human malignancies [62]. NGM is likely the better comparison to find GC-specific markers, as protein changes resulting from other processes, such as inflammation, can be excluded. Here, 181 proteins are changed and 87 are upregulated in GC (network, functional classification for HPp/n, Supplementary Figures S70–S73). While ITIH4 failed to be a potential marker in this group comparison, FZD6 remained as such, for both HPp and HPn. In addition, kallistatin (SERPINA4) was noticeable, and was previously described as a suppressor in cancer development [63] Some of the proteins discussed above (S100A9, CRP, GSTT1) also remain. In order to find the shared and differing proteins with respect to *H. pylori* infection, Venn analysis was performed, and this shows that 20 proteins (Supplementary Table S4) are common upregulated proteins in GC vs. V and GC vs. NGM, HPp/n; they present a *H. pylori* infection-independent selection of potential GC markers. For the GC vs. NGM HPp/n comparison, 41 common proteins are detected. 

#### 2.3.5. GC 

The effects of gender or infection status within the GC group are minor (PCAs, Supplementary Figure S58E,G,H). Less than 20 shortlisted proteins contribute. Of note is the appearance of double the amount of abundant immunoglobulin heavy constant γ2 (IGHG2) in females than in males [47,59]. Of the 11 proteins contributing to group separation of samples from 1st stage and advanced GC (PCA, Supplementary Figure S58F), 10 are more abundant in advanced GC. Among them is fibrinogen β (FGB), which is 6.7-fold more concentrated in advanced GC (in agreement with other studies), which associates elevated plasma levels with GC progression [64]. Ficolin 3 (FCN3, 2.7-fold up in aGC) was already suggested as a potential prognostic serum biomarker in esophageal cancer [65]. The only upregulated protein in 1st stage GC is fibulin 1 (FBLN1). Reduced expression was found in GC cell lines [66], so this protein could be investigated as a possible marker for tumor progression, having a high concentration only in early GC.

#### 2.3.6. Other Comparisons

PCA demonstrates group separation for the shortlisted proteins of V and NGM vs. U and gastritis, and the results are shown in Supplementary Figure S60 (PCAs NGM vs. gastritis, Supplementary Figure S60G,H). Proteins, which are more abundant in GC (87), U (25), and combined MaG/MoG/PanG (117) vs. NGM, were screened and 19 GC-specific proteins were extracted (Supplementary Table S5). The proteins, which are only detected in HPp samples (24), are also available in Supplementary Table S5.

#### 2.3.7. Selection of Marker Proteins

From the result of the above considerations, prospective GC serum markers were chosen. We thereby focus on those proteins, which are upregulated in GC, independent of *H. pylori* infection, and whose analytical performance is above average. The selection of 10 proteins (10GC-P) is presented in Table 4. 

## 3. Discussion 

Cancer is a complex genetic disease, but energy metabolism is a major hallmark, and was hailed as the “Achilles’ heel” of the disease [19]. We also measured proteins such as hexokinase (HK), transketolase (TKT), and ENO1, among many other enzymes, especially in biopsies. However, the more detailed the analysis became, the more other protein classes became important. This was exemplified above for ENO1, which was upregulated in GC biopsies vs. NGM in our initial analysis, but was not among the proteins found when the analysis was specified to antrum samples. Other proteins from our lists, which were named previously as GC markers by other authors, such as phospholipid transfer protein (PLTP), tripeptidyl peptidase 1 (TPP1) [67], and annexin A8 (ANXA8) [68], also did not make it to the final marker panel. In fact, we were surprised to find a link to cancer, and even specifically a link to GC, for the majority of our shortlisted proteins. 

There are a number of reasons for the discrepancy between the small selection of routinely used clinical GC markers [5] and the abundance of proposed proteins for the purpose. First is the availability of large enough patient cohorts in order to overcome biological variation, which is often limiting, especially in rare diseases. Moreover, cohorts tend to be biased; volunteer donors are often younger than patients. We were fortunate to work with a large cohort, but not every question we asked of subgroups was answered with sufficient experimental backing, e.g., the comparison between 1st stage and advanced GC; there were only three samples available for the former, and one of them came from a male. Therefore, we present these data with a word of caution. 

Second, even if the tissue comes from the same organ, protein profiles might differ regionally [7]. We also noted remarkable differences between gastric biopsies of corpus and antrum. In clinical practice, it is not always possible to sample the most desirable tissue. The effect such sampling issues has on the final protein profile is illustrated by the fact that of the 159 proteins upregulated in GC vs. NGM (corpus samples included), only 106 are shared in the antrum-only analysis. 

Third, marker research in oncology is often based on tissue comparisons, because it is a logical choice to investigate the tumor directly vs. healthy tissue. We also used this approach as part of our study, but we feel that a truly useful clinical GC marker should be accessible using minimally invasive procedures, such as a blood draw. Even the collection of gastric juice, where α1-antitrypsin precursor (SERPINA1) was suggested as biomarker [69], is difficult, and not pleasant for the patient. We compared the gastric biopsy and serum datasets, and did not find much overlap in the extracted protein profiles. Only six proteins are shared, for instance, in the analyses between the sera of MiGa cases and polyp biopsies vs. NGM. 

While blood is a great study source, being readily available, it is also an overly complex medium, with an extremely high dynamic range in protein concentration of more than 10 orders of magnitude [70]. Nevertheless, we detected a panel of 10 prospective biomarkers, including FZD6, which is the regulator of the Wnt pathway in different cancer types [62].

A fourth point is the fact that any analytical procedure comes with limitations. A comparison of our results to the data obtained by 2D-DIGE [71] shows the same protein hits, but they are not among the marker candidates in our case. For GC-associated genes identified by gene expression analysis [72], no direct correlation was expected, as, in the majority of cases, there is no correspondence between the gene copy number and the change of the respective protein [73]. Other authors suggest fibrinogen α chain precursor (FGA), α-2-HS-glycoprotein precursor (AHSG), and apolipoprotein A-I precursor (APOA1) as diagnostic serum biomarkers for GC, based on MALDI-TOF analysis [74]. We find these proteins in different subsets of our comparative analyses, e.g., APOA1 and AHSG in GC vs. U, and FGA in NGM vs. GC, but, with the exclusion of the shared proteins with the gastritis and U datasets, they are not significant anymore. 

Fifthly, for omics studies, the entire workflow, from the clinic to the lab, must be standardized to avoid method-dependent flaws, or false-positive results. However, procuring samples for research in a clinical environment can be exceedingly difficult. We had to remove six GC biopsies from the analysis, because their sampling procedure was different, which we only learned from our experiments, when the results deviated. 

With our study, we provide a wealth of information concerning protein expression in gastroduodenal diseases. So far, we mined this dataset for proteins prominent in GC, and present a 10-member panel of GC-specific serum proteins (10GC-P), as well as a 29-member panel of prospective indicators for early stage GC (29preGC-P, for information on the proteins, see Supplement). The latter panel was generated from MiGa cases that were flagged with potential pre-cancerous histopathology [42], and whose serum profiles were significantly different from those of MiGn. The 10GC panel was assembled from upregulated proteins in GC, independent of *H. pylori* infection, and only including proteins whose analytical performance was above average. 

MS spectra quality is, in fact, point six of above discussion. As MS-based proteomics examines ten thousands of spectra in one run, and provides thousands of protein IDs, there is always potential for false-positive matches. Filter-based software algorithms also assign peptide hits to weak spectra, which is both blessing and curse [75]. Manual spectral assessment alleviates some of these problems, but validation with orthogonal methods of each proposed marker protein is still necessary.

Prognostic value in GC patients was assigned to the albumin-fibrinogen relationship in serum [51], and we also found drastic concentration differences between sera of gastritis patients (hypoalbuminemia, high levels of fibrinogen), and those of all the other cases (V, NGM, U, GC), including MiGa. It appears that gastritis constitutes a state of acute-phase inflammation, in contrast to U and GC [76]. We have clear evidence that FAR confidently distinguishes gastritis from other gastroduodenal diseases, such as U and GC, and, thus, has the potential to reduce the number of endoscopies by more than half, as calculated based on our project cohort. As Figure 5 illustrates, in the current situation, symptomatic patients see a specialist following unsuccessful primary care and are, if symptoms do not improve, referred to endoscopy. Only histopathological examination of biopsies clarifies the nature of the stomach problems. We propose to intervene early, at the primary to secondary care level, with a FAR serum lab test, which identifies the gastritis patients, and eliminates the need for endoscopy for this group. In addition, we suggest that the *H. pylori* infection status is determined, because most of our MiGa cases are *H. pylori* positive, while all MiGn cases are negative. In this way, based on the patient numbers in our cohort, less than half of the endoscopic procedures (44%) were required in the present study. 

## 4. Experimental Section

More details are provided in the Supplement and the Supplementary File “analysis”.

### 4.1. Permissions

Ethical approvals were obtained from the Ethical Technical Committee of the Pakistan Institute of Nuclear Science and Technology (PINSTECH), Islamabad (Ref.-No. PINST/DC-26/2017); the Bioethics Committee of Quaid-i-Azam University, Islamabad (Ref.-No. BBC-FBS-QAU2019-159); the Institutional Research Forum of Rawalpindi Medical University, Rawalpindi (Ref.-No. R-40/RMU); and the Ethics Committee of the University of Münster, Germany (Ref.-No. 2021-339-f-N). Informed written consent was obtained from each participant.

### 4.2. Protein Isolation and Analysis

Total protein from gastric biopsies was extracted using T-PER extraction reagent (Thermo Fisher Scientific, Waltham, MA, USA), according to the instructions of the manufacturer; serum was processed with lysis buffer. Samples were dried and stored at −80 °C at PINSTECH, until transport to IZKF Core Unit Proteomics for further analysis. They were processed according to an established protocol for filter-aided tryptic digestion [77]. Peptide solutions were analyzed using reversed-phase LC, coupled with high-definition (HD) MS, with Synapt G2 Si/M-Class nanoUPLC (Waters Corp., Manchester, UK) using C18 µPAC columns (trapping and 50 cm analytical; PharmaFluidics, Ghent, Belgium), with a 90 min gradient (solvent system 100% water versus 100% acetonitrile, both containing 0.1% formic acid) as described [77]. 

### 4.3. Data Analysis/Statistics

Data were analyzed, and PCA was performed with Progenesis for proteomics (QIP, Nonlinear Diagnostics/Waters Corp.), using the human Uniprot database (UP000005640, January 2021, FDR 4%). Shortlists of the protein output were created by demanding protein assignment by at least two peptides, a fold value of at least 2, and a significance of ANOVA *p* ≤ 0.05. Heatmaps were created using the heatmapper software tool [78], and Venn diagrams with InteractiVenn [79]. Geneontology analysis was performed with the Panther classification system [80], and protein network analysis with String (String Consortium 2021, ELIXIR Core Data Resource) and NetworkAnalyst [81]. T-tests (two-tailed) were performed using SPSS (Version 26IBM Corp., Armonk, NY, USA).

## 5. Conclusions

This investigation compared the biopsy and serum protein profiles of 219 patients with gastroduodenal diseases, namely gastritis, U, and GC, with the goal of elucidating robust serum markers specific for GC. An advantage was the availability of a wealth of metadata, clinical information, and histopathological evaluation, along with *H. pylori* infection status, which were cross-analyzed [11]. We elucidated two GC serum marker panels, 29preGC-P and 10GC-P, for early stage and advanced GC, respectively. The majority of these proteins were previously discussed in the context of cancer or GC. For most of them, individual commercial tests are already available. We envision the design of multiplex assays, incorporating several to all of the panel members, in order to gain a level of specificity that cannot be achieved by testing a single protein alone. The prime example in this respect is CRP, which is part of the 10GC panel, but is also routinely used as a general inflammation marker. We will employ targeted MS to set up a dedicated multiprotein detection method, which has, based on triple-quadrupole technology, the potential to find application in clinical labs. MS methods can be expensive, but so can antibody-based detection, and both technologies have their advantages and disadvantages. In any case, the test must be reasonably priced in comparison to endoscopy to truly find application in clinical practice. 

In that light, FAR-based gastritis diagnosis may be a quick way forward. Both albumin and fibrinogen are routinely measured in the clinical laboratory anyway. While the effect was discovered in MS data, it does not require this comparatively expensive technique for its use. Still, the MS-based observations need to be validated using routine lab assays. These are not as sensitive as MS, but we are confident that the observed dramatic concentration changes will be detectable. The determination of FAR during primary or secondary care would significantly reduce the number of referrals to endoscopy—in our study, 56% of all cases. With an average cost of $2.750 for one endoscopy in the United States [82], this amounts to fictive savings of $313.500 for the 114 gastritis patients in our study, who would not need this examination. 

Preventive measures are in high demand. The protein marker panels presented in this work will contribute to improved GC diagnostics once they have been transferred from a research result to a practical tool.

## Figures and Tables

**Table 1:** Patient information. GU—gastric ulcer, DU—duodenal ulcer, PU—peptic ulcer, 1GC—1st stage GC, aGC—advanced GC.

Biopsies					NGM	MiG	MoG	MaG	PanG	U	GC	Total	%
***Patients***					12	11	11	11	5	12	13	75	
Female					4	8	6	3	2	4	5	32	43
Male					8	3	5	8	3	8	8	43	57
Age < 46					9	7	7	10	-	3	6	43	56
Age > 45					3	4	4	1	5	9	7	34	44
***Samples***					12	22	22	22	5	24	26	133	
Female					4	16	12	6	2	8	11	59	44
Male					8	6	10	16	3	16	15	74	56
Normal mucosa—N site					12	11	11	11	-	12	11	68	51
Diseased mucosa—D site					-	11	11	11	5	12	15	65	49
*H. pylori* positive—HPp					9	14	10	16	3	20	21	93	70
*H. pylori* negative—HPn					3	8	12	6	2	4	5	40	30

**Serum**	**V**	**NGM**	**MiG**	**MoG**	**MaG**	**PanG**	**GU**	**DU**	**PU**	**1GC**	**aGC**	**Total**	**%**
***Patients***	17	26	30	51	30	12	16	5	2	10	20	219	
Female	9	14	13	30	11	3	6	2	-	4	6	98	45
Male	8	12	17	21	19	9	10	3	2	6	14	121	55
Age < 46	17	21	23	40	14	5	6	2	2	7	4	141	64
Age > 45		5	7	11	16	7	10	3	-	3	16	78	36
HPp	10	15	16	25	18	8	9	4	1	7	16	129	59
HPn	7	11	14	26	12	4	7	1	1	3	4	90	41

**Table 2:** 25preGC-panel: proteins upregulated in MiGa vs. NGM, and not shared with MiGn vs. NGM (Venn analysis in Supplementary File “serum_analysis”). GC-specific serum proteins chosen for validation as serum markers and their detection results. Sorted by decreasing intensity.

Accession	Name	Description	Peptide Count	Unique Peptides	Confidence Score	Max Fold Change
O95872	GPANK1	G patch domain and ankyrin repeat-containing protein 1	7	2	40	2.5
P0DP01	IGHV1-8	Immunoglobulin heavy variable 1–8	6	2	46	2.3
P18564	ITGB6	Integrin β-6	5	5	34	2.1
P04433	IGKV3-11	Immunoglobulin κ variable 3–11	5	2	46	4.7
P22352	GPX3	Glutathione peroxidase 3	5	2	30	2.3
Q5UE93	PIK3R6	Phosphoinositide 3-kinase regulatory subunit 6	5	1	25	3.3
P0DP08	IGHV4-38-2	Immunoglobulin heavy variable 4-38-2	5	1	45	2.4
P23786	CPT2	Carnitine O-palmitoyltransferase 2_ mitochondrial	4	1	23	3.1
Q64ET8	FRG2	Protein FRG2	4	1	28	4.6
Q9H0U6	MRPL18	39S ribosomal protein L18_ mitochondrial	4	2	20	4.1
P41247	PNPLA4	Patatin-like phospholipase domain-containing protein 4	4	1	18	2.4
Q15032	R3HDM1	R3H domain-containing protein 1	4	2	30	18.2
Q9NR63	CYP26B1	Cytochrome P450 26B1	3	1	19	3.1
O00255	MEN1	Menin	3	1	18	2.1
P08865	RPSA	40S ribosomal protein SA	3	1	15	7.9
P40855	PEX19	Peroxisomal biogenesis factor 19	3	2	16	2.7
O96007	MOCS2	Molybdopterin synthase catalytic subunit	3	1	24	2.5
Q92841	DDX17	Probable ATP-dependent RNA helicase DDX17	3	1	17	2.6
Q6WQI6	HEPN1	Putative cancer susceptibility gene HEPN1 protein	3	2	24	2.3
O43692	PI15	Peptidase inhibitor 15	2	1	24	2.9
Q96JF0	ST6GAL2	Β-galactoside α-2_6-sialyltransferase 2	2	2	17	2.4
Q5VZM2	RRAGB	Ras-related GTP-binding protein B	9	1	55	8.4
P06702	S100A9	Protein S100-A9	7	6	57	2.0
P0DP09	IGKV1-13	Immunoglobulin kappa variable 1-13	7	4	74	3.5
P30711	GSTT1	Glutathione S-transferase theta-1	9	5	54	2.1

**Table 3:** Calculation of FAR using average MS intensities (FAR-MS, intensity fibrinogen/intensity albumin). The result was multiplied by 100, as in reference [55]. MS intensities are also set in relation (Cmp) to the reference values for albumin (35–55 g/L) and fibrinogen (2–4 g/L) [51], using an approximate medium value of 45 and 3 g/L, respectively, for the V group, to calculate FAR-Cmp. In order to show the fold change to normal values, the average MS intensities for the respective groups are divided by those seen in V (Factor to V). For details, see Figure 5, Supplementary File “serum_analysis”.

		Albumin			Fibrinogen			FAR	
	**Group**	**Cmp/g/L**	**MS int./counts**	**Factor to V**	**Cmp/g/L**	**MS int./counts**	**Factor to V**	**Cmp/g/L**	**MS**
**Ref.**	**V**	**45.0**	21659682		**3.0**	53631		**6.7**	0.25
	**1GC**	35.3	16989199	0.8	1.1	19117	0.4	3.0	0.11
**High albumin**	**NGM**	88.9	42806672	2.0	6.8	120974	2.3	7.6	0.28
	**U**	57.2	27549815	1.3	3.4	60796	1.1	5.9	0.22
	**aGC**	64.6	31103607	1.4	2.9	50985	1.0	4.4	0.16
	**MiGa**	97.1	46721703	2.2	3.0	54459	1.0	3.1	0.12
**Low albumin/high fibrinogen**	**MiGn**	1.6	756160	0.03	31.9	570425	10.6	2031.1	75.44
	**MoG**	7.0	3364998	0.16	139.4	2491549	46.5	1993.6	74.04
	**MaG**	3.1	1494820	0.07	81.9	1463330	27.3	2635.7	97.89
	**PanG**	1.3	633851	0.03	29.5	528261	9.8	2243.9	83.34

**Table 4:** 10GC-P: GC-specific serum proteins chosen for orthogonal validation as serum markers, sorted by decreasing intensity, and their detection results (fold value for GC vs. NGM, average intensities from 2799–242760 counts in GC sera).

Accession	Name	Description	Peptide Count	Unique Peptides	Confidence Score	Max Fold Change
P19827	ITIH1	Inter-α-trypsin inhibitor heavy chain H1	80	60	596	3.1
O60353	FZD6	Frizzled-6	27	19	198	2.3
Q8N608	DPP10	Inactive dipeptidyl peptidase 10	14	10	103	3.4
P29622	SERPINA4	Kallistatin	22	17	147	2.6
Q02952	AKAP12	A-kinase anchor protein 12	15	6	98	2.8
P06702	S100A9	Protein S100-A9	7	6	57	2.6
A5A3E0	POTEF	POTE ankyrin domain family member F	43	4	295	2.3
Q02641	CACNB1	Voltage-dependent L-type calcium channel subunit β-1	12	5	82	2.0
P02741	CRP	C-reactive protein	11	7	83	3.4
Q86T90	KIAA1328	Protein hinderin	9	3	62	2.1

## Data Availability

All data are provided in the Supplement or are available on request.

## Supplementary Materials

The following supporting information can be downloaded at: https://www.mdpi.com/article/10.3390/molecules27092857/s1, Supplement280422.pdf; Supplementary Excel Files: Supplement_serum, Supplement_serum_analysis, Supplement_tissue, Supplement_tissue_analysis. References: [13,46,62,63,77,78,79,80,81,83,84,85,86,87,88,89,90,91,92,93,94,95,96,97,98,99,100,101,102,103,104,105,106,107,108,109,110,111,112,113,114,115,116,117,118,119]

## Sample Availability

Sample availability was limited to the authors.

## Abbreviations

GC—gastric cancer, MiG—mild gastritis, MiGa—atypical MiG, MiGn—normal MiG, MoG—moderate gastritis, MaG—marked gastritis, PanG—pan gastritis, NGM—normal gastric mucosa, HPp—*H. pylori* positive, HPn—*H. pylori* negative, D—disease site, N—normal site, HDMS—high definition mass spectrometry, PCA—principal components analysis, V—volunteer, LC—liquid chromatography.
