# Supplementary material for: Gastric Cancer Pre-Stage Detection and Early Diagnosis of Gastritis Using Serum Protein Signatures
Source: Molecules. 2022 Apr 30;27(9):2857. doi: 10.3390/molecules27092857 (PMC9099457; doi:10.3390/molecules27092857)
Supplement: Supplementary file 1 [file molecules-27-02857-s001.zip › supplementary materials-1691505/suppl_rev/supplementary materiasl-1691505.pdf]

Gastric cancer pre-stage detection and early diagnosis of gastritis using serum protein signatures

Shahid Aziz[1, 2, 3], Faisal Rasheed[1], Rabaab Zahra[2], Simone König[3*],

[1]BreathMAT Lab, Pakistan Institute of Nuclear Science and Technology, Islamabad, Pakistan

[2]Department of Microbiology, Faculty of Biological Sciences, Quaid-i-Azam University Islamabad, Pakistan

[3]IZKF Core Unit Proteomics, University of Münster, Germany

***Corresponding author:** koenigs@uni-muenster.de

Experimental

Gastric biopsy sampling

Gastric biopsy specimens were, if possible, collected from normal (N) and adjacent diseased (D) parts of the stomach antrum (with the exception of three NGM samples from corpus) during gastroduodenal endoscopy. Two biopsy specimens each were immerged in 10% formalin for histopathological examinations (HE) and one N and D specimen each were wrapped in aluminum foil and transported in liquid nitrogen to BreathMAT Lab, PINSTECH, for protein extraction.

Diagnosis of H. pylori infection

^{13}C urea breath test (UBT): Pre-dose and post-dose breath samples were collected from study participants as described previously [13]. Briefly, after overnight fasting, the pre-dose breath sample was taken. A dose containing 75 mg ^{13}C enriched urea (Cambridge Isotope Laboratories, USA) was given to the patients and the post-dose breath samples were collected in a 12 ml Exetainer® vial (Labco, UK) after 30 min. Both breath samples were analyzed to assess the $^{13}CO_2/^{12}CO_2$ ratio using BreathMAT^{plus} mass spectrometer (Thermo Finnigan, Germany) and Delta V mass spectrometer (Thermo Scientific, USA). A change in the δ ^{13}C value over baseline of more than 3‰ was considered positive.

HE: The presence of *H. pylori* within gastric biopsy specimens was evaluated by routine histopathological methods. Briefly, formalin fixed paraffin embedded tissues were sectioned and stained with hematoxylin/eosin and giemsa (Merck).

Tissue preparation for protein expression analysis

Total protein from gastric biopsies was extracted using T-PER ™ extraction reagent (Thermo Scientific, USA) according to the instructions of the manufacturer, dried and stored at -80 °C at PINSTECH until transport on dry ice to IZKF Core Unit Proteomics for further analysis.

Samples were processed according to an established protocol with slight modifications. Briefly, 200 µl Milli-Q water were added to each sample for re-dissolvation and the solution was treated in an ultrasonic M homogenizer (80% amplitude, 0.6% cycles, 6 min; Sartorius, Germany). Sodium desoxycholate (2 µl, 2%; Thermo Scientific, USA) was added to the solution followed by short vortexing and 15 min incubation at room temperature (RT). Then, 20 µl of 100% trichloroacetic acid (Sigma-Aldrich) were added to the tubes, which were immediately vortexed. Precipitation proceeded at RT for 1 h. After centrifugation (30,000 × g, 4 °C, 30 min), the pellet was washed twice with ice-cold acetone (400 µl), each time being vortexed and shaken on IKA Vibrax VXR Basic (5 min, IKA-Werke, Germany). The samples were placed in a freezer (-20 °C, 15 min). Subsequently, the supernatant was discarded after centrifugation (12,500 × g, 4 °C, 15 min; Hettich Lab Technology, Germany). Residual acetone was allowed to evaporate in a hood and 100 µl of lysis buffer (4 M urea, 50 mM Tris base, 4 g sodium dodecyl sulfate (SDS), 10 mM tris (2-carboxyethyl) phosphine (TCEP; all from SERVA, Germany)) was added. The protein concentration was determined by using the Pierce® BCA protein assay kit – reducing agent

compatible (Thermo Scientific, USA) according to the manufacturer's instructions with Ultraspec 2000 UV/visible spectrophotometer (Pharmacia Biotech, USA). The standard curve was generated with bovine serum albumin.

Filter-aided tryptic digestion

Nanosep® centrifugal filter units 10 kDa cut-off (Pall Corporation, USA) were rinsed with 500 µl 1% aqueous formic acid (FA, (EMD Millipore Corporation, USA) containing 5% acetonitrile (ACN; Honeywell, USA) three times to eliminate production residues. The total of 10 µg of each sample was added to the filter unit and the volume was adjusted to 200 µl with urea buffer (urea 4 M, Tris base 100 mM) followed by vortexing and centrifugation (12,500 × g, 15 min, RT). The samples were washed with 100 µl urea buffer and centrifuged (12,500 × g, 15 min, RT). For reduction, 100 µl of 50 mM dithiothreitol (DTT; SERVA, Germany) solution was added to the filter unit and incubated at RT on Vibrax (500 rpm, 45 min). The filter units were centrifuged (12,500 × g, 15 min, RT) and washed with 100 µl of urea buffer. For alkylation, 100 µl of 50 mM iodoacetamide (SERVA, Germany) solution was added to each filter unit and incubated in darkness on Thermomixer Comfort (RT, 500 rpm, 30 min; Eppendorf, Germany) followed by centrifugation. The reaction was quenched by adding 100 µl 50 mM DTT and a second incubation in the dark (15 min, 500 rpm). The filter units were centrifuged (12,500 × g, 15 min, RT) and washed four times with 300 µl NH_4HCO_3 (50 mM, Sigma-Aldrich) containing 10% ACN followed by centrifugation. The filter units were set into new PALL collection tubes and 100 µl of 0.002 µg/µl trypsin (1:50 enzyme/substrate ratio; SERVA, Germany) digestion solution was pipetted into each filter unit. The units were sealed with parafilm and placed on a thermoshaker (Hettich Lab Technology, Germany) for overnight digestion at 37 °C and 800 rpm. Subsequently, the filter units were centrifuged and washed three times with 40 µl 0.1% FA / 5% ACN solution. Peptide solutions were collected, dried using a Speed Vac Concentrator (Thermo Scientific, USA) and stored until further use. For subsequent MS analysis, samples were redissolved in 20 µl 5% ACN / 0.1% FA in order to generate a stock solution of 500 ng/ µl, which was diluted 1:1 for MS.

Blood sampling

Venous blood (5 ml) was collected in BD Vacutainer SST II Advance tubes (BD Corporates, USA) and centrifuged (10 min, 20,000 x g; Hettich Lab Technology, Germany). The serum was transferred to new sterile Eppendorf tubes, centrifuged again (30 min, 20,000 x g) and the cell-free supernatant was collected.

Serum protein isolation

Serum samples (250 and 500 µl) were processed with 500 µl or 1 ml, respectively, lysis buffer (8 M urea, 100 mM Tris base, 2% w/v SDS) and centrifuged at 16,000 x g for 5 min. The supernatant was pipetted into a new sample tube and 100 µl TCEP (10 mM in H_2O) was added. After vortexing and centrifugation (5 min, 16,000 x g) the supernatant was lyophilized by means of CentriVap Concentrator (Labconco, USA). Samples were stored at -80 °C in BreathMAT Lab,

PINSTECH, and transported on dry ice to IZKF Core Unit Proteomics, University of Münster, Germany. The dried serum protein samples were re-solubilized as described [83]. Briefly, the dry pellet was disintegrated by means of Vibrating Mixer Mill Type MM 300 (20 min, 30 Hz; Retsch, Germany) and 1 ml of 1 M guanidine hydrochloride (Sigma-Aldrich) containing 20% ACN was added. Vortexing generated a milky homogenous emulsion, which was further processed for MS analysis.

Serum preparation for expression analysis

Serum proteins were treated as described above for tissue with few modifications. A serum equivalent of 10 µl was used for digestion. For re-dissolvation of tryptic peptides 100 and 200 µl, respectively, were used, depending on the original serum amount.

Proteomic analysis

Peptide solutions (3 µl for tissue, 0.5 µl for serum) were analysed by reversed-phase liquid chromatography (LC) coupled to high-definition (HD)MS with Synapt G2 Si / M-Class nanoUPLC (Waters Corp., Manchester, UK; resolution mode with resolution >20,000 and additional ion mobility separation) using PharmaFluidics C18 µPAC columns (trapping and 50 cm analytical; PharmaFluidics, Ghent, Belgium) with a 90 min gradient (solvent system 100% water versus 100% ACN, both containing 0.1% FA) as described [77]. Data were analysed using Progenesis for Proteomics (QIP, Nonlinear Diagnostics/Waters Corp., Hi-3 relative quantification) and the human Uniprot database. One missed cleavage was allowed, carbamidomethylation was set as fixed and methionine oxidation as variable modifications, respectively. A false-discovery rate < 4 was set. QIP was also used for principal component analysis (PCA). Shortlists of the protein output were created by demanding protein assignment by at least two peptides, a fold value of at least 2 and a significance of ANOVA $p \leq 0.05$. Heatmaps were created using the heatmapper software tool [78] and Venn diagrams with InteractiVenn [79]. Geneontology analysis was performed with the Panther classification system [80] and protein network analysis with String (String Consortium 2021, ELIXIR Core Data Resource) and NetworkAnalyst [81].

Table S1: Histopathological evaluation of GC biopsy specimens. All specimens were of the diffused type according to the Lauren classification.

**Endoscopic evaluation**	**HE results**	
	Differentiation	WHO classification
1-polyp<br>2-large infiltrative growth	1-adenocarcinoma<br>2-signet ring cell carcinoma	1-tubular<br>2-poorly cohesive
1	1	1
1	1	1
1	1	1
2	1	1
2	1	1
2	1	1
2	1	1
2	2	1
1	2	2
1	2	2
2	2	2
2	2	2
2	2	2

Tissue analysis

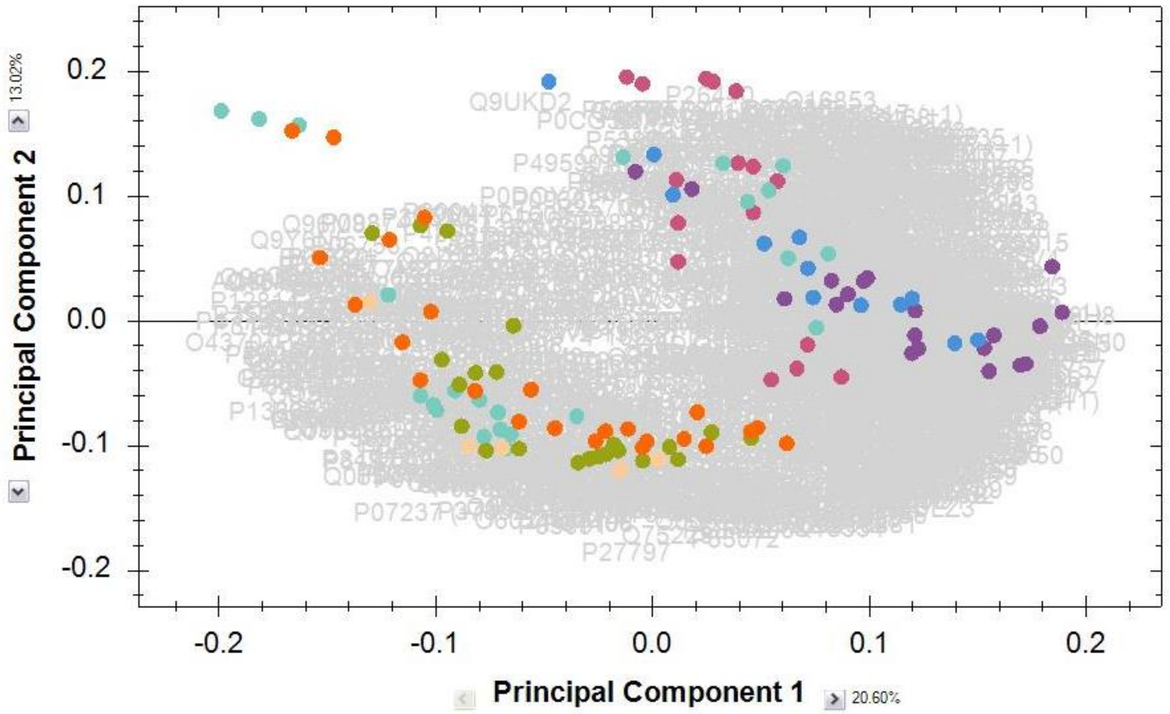

Figure S1: PCA of MoG (orange), MaG (green), MiG (purple), PanG (beige), (U) turquoise, NGM (blue) and GC (berry).

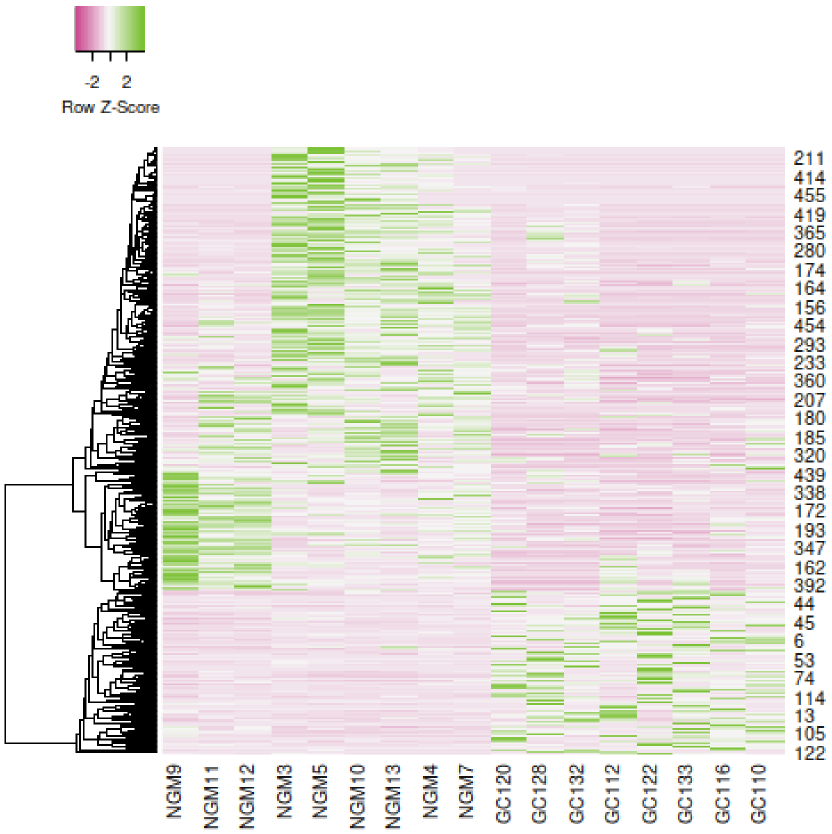

Figure S2: Heatmap (Spearman rank correlation) of the shortlisted proteins of HPpD NGM and GC samples.

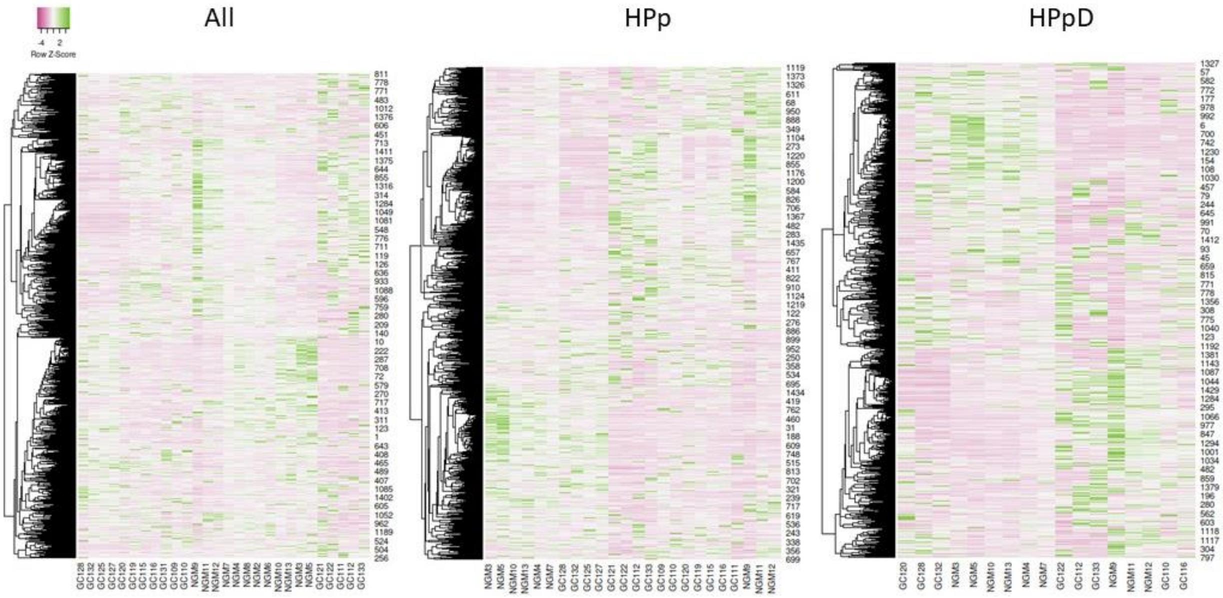

Figure S3: Heatmaps (Spearman rank correlation, rows and columns sorted) of the 1500 most abundant proteins of NGM and GC samples (minimum 2 peptide matches).

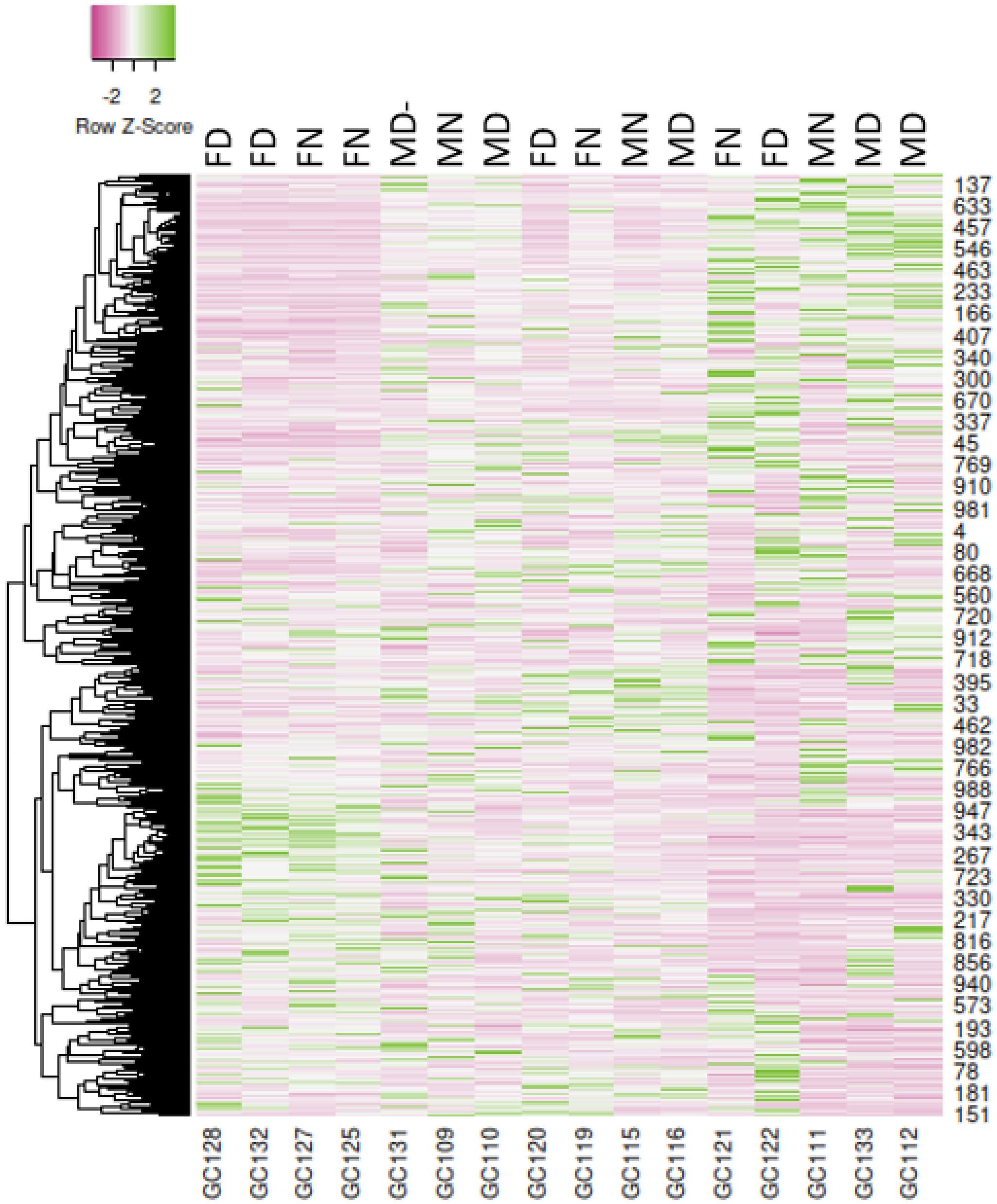

Figure S4: Heatmap (Spearman rank correlation) of the GC runs (1000 most abundant proteins, confidence 100). F – female, M – male, D and N sites, (-) – single HPn sample

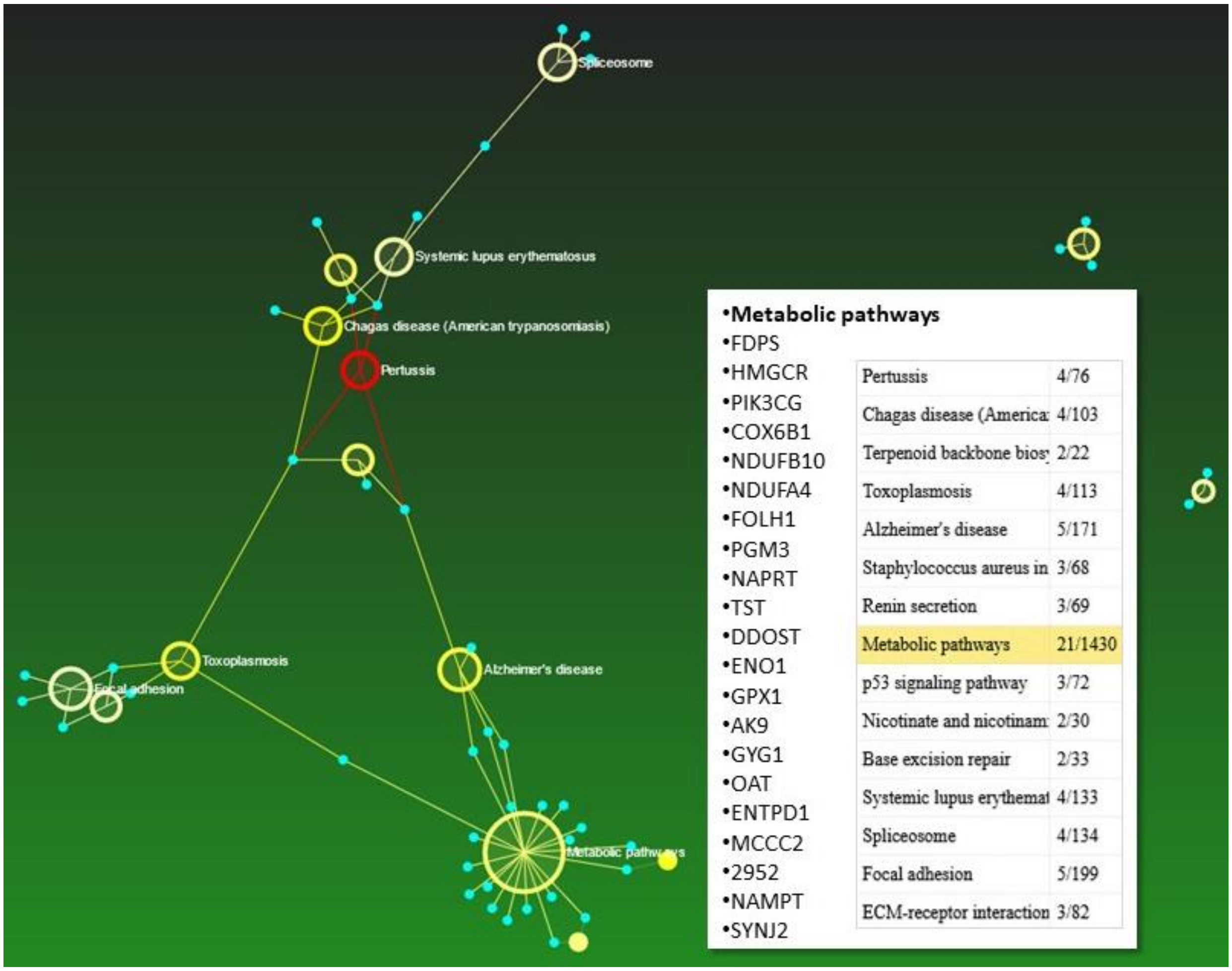

Figure S5: Enrichment ORA network (NetworkAnalyst) of shortlisted proteins upregulated in GC *vs.* NGM.

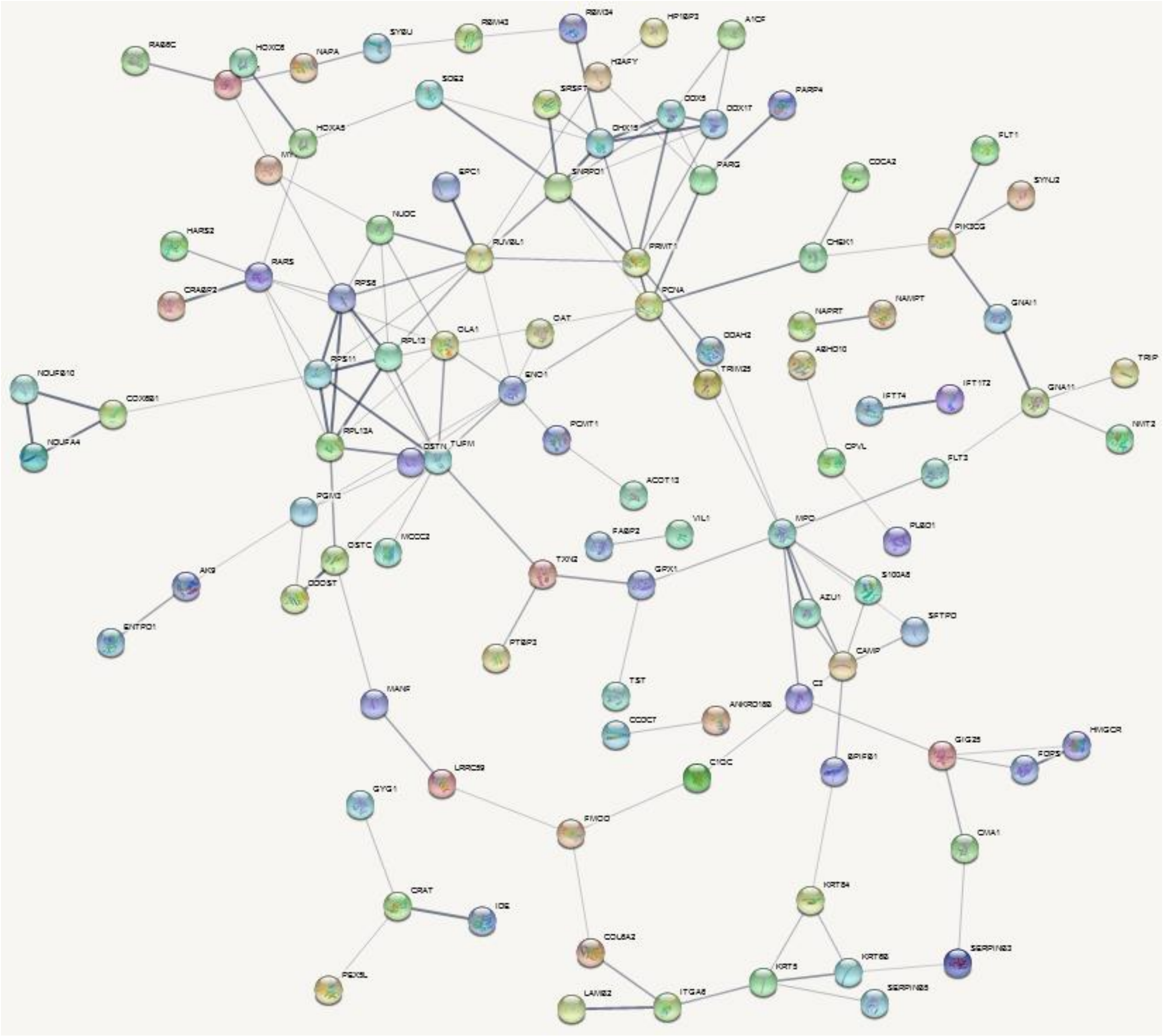

Figure S6: String network of shortlisted proteins upregulated in GC *vs*. NGM.

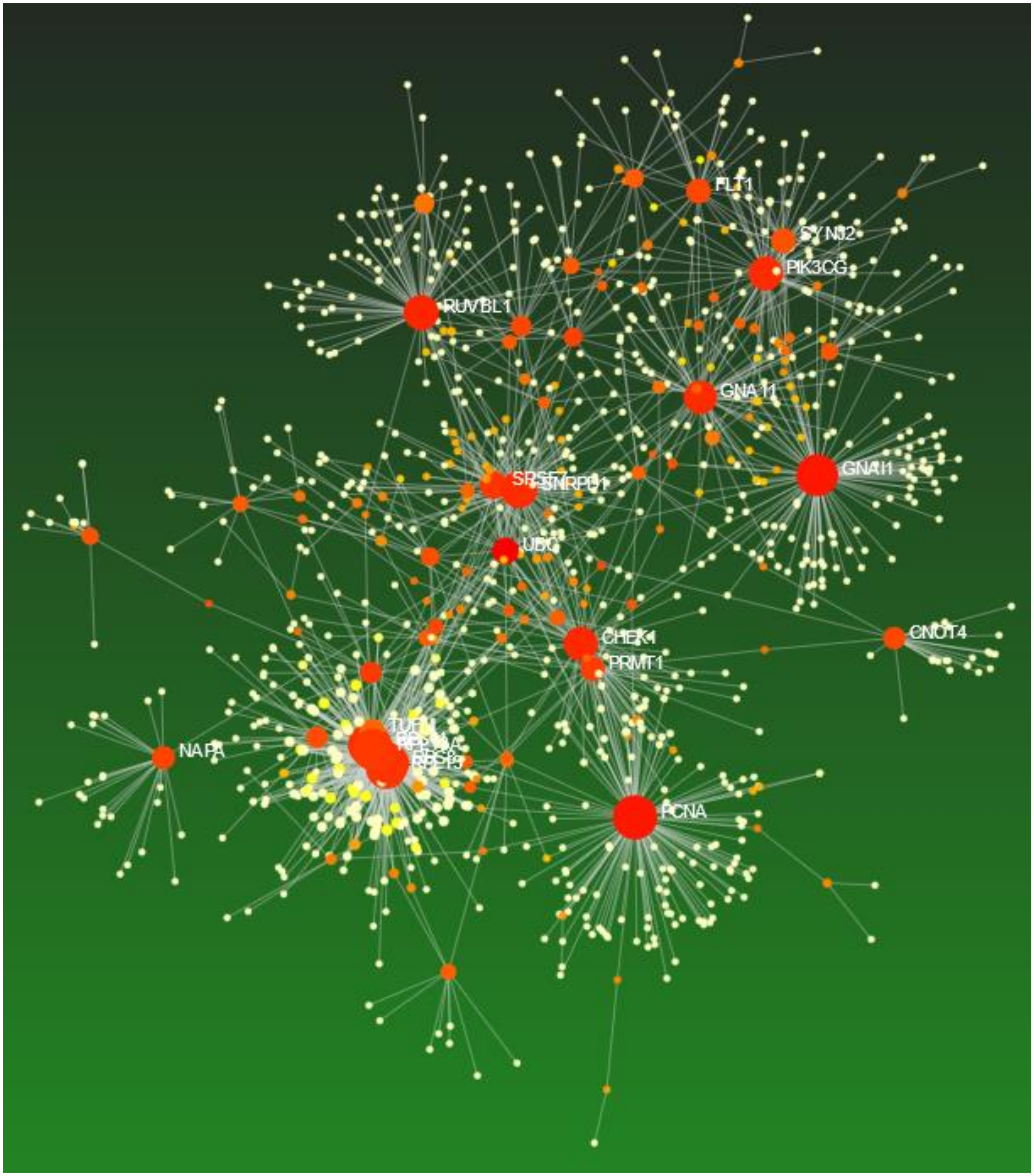

Figure S7: Larges subnetwork obtained with NetworkAnalyst for shortlisted proteins upregulated in GC *vs.* NGM.

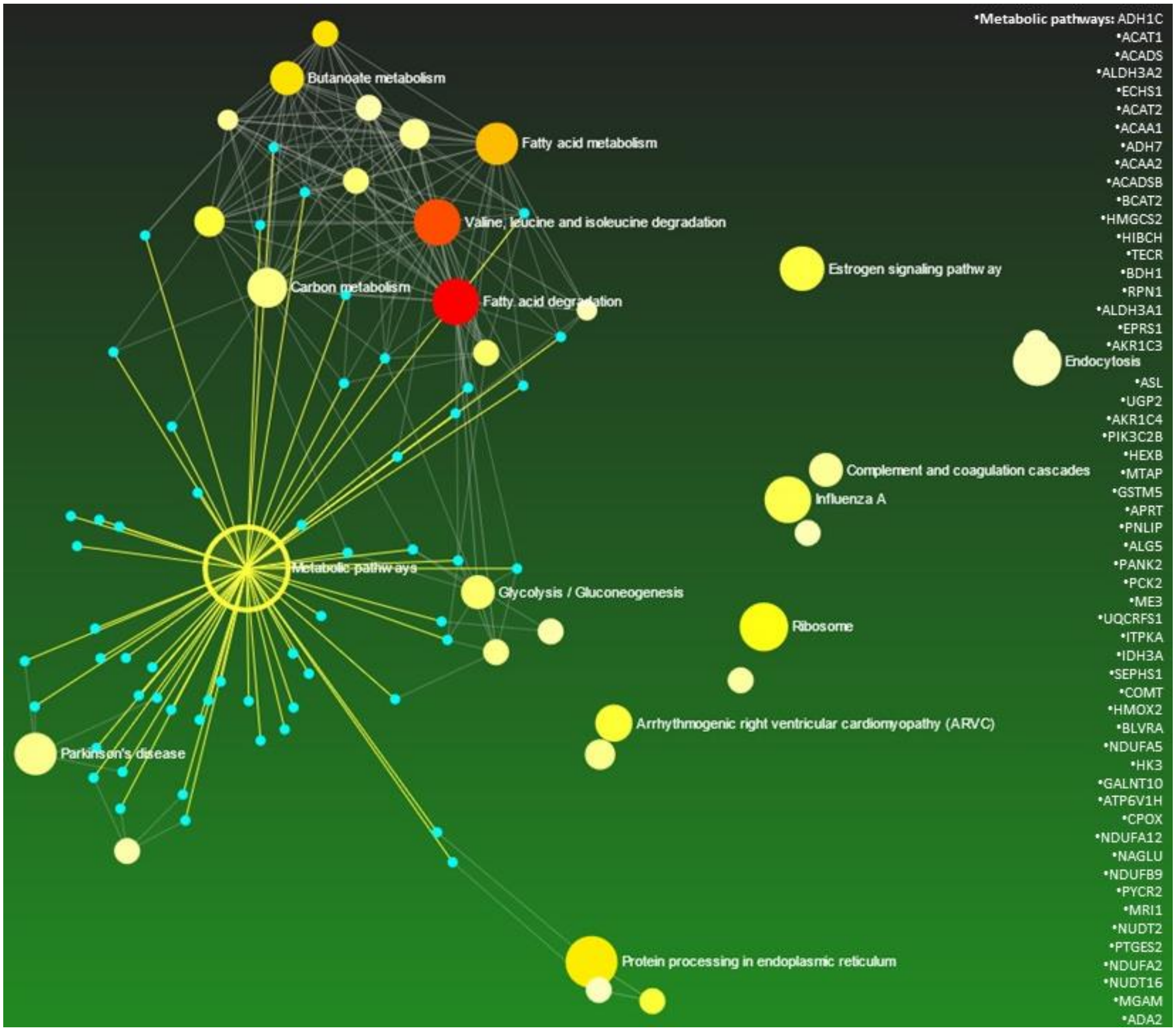

Figure S8: Enrichment ORA network (NetworkAnalyst) of shortlisted proteins downregulated in GC *vs.* NGM and participating proteins in metabolic pathways.

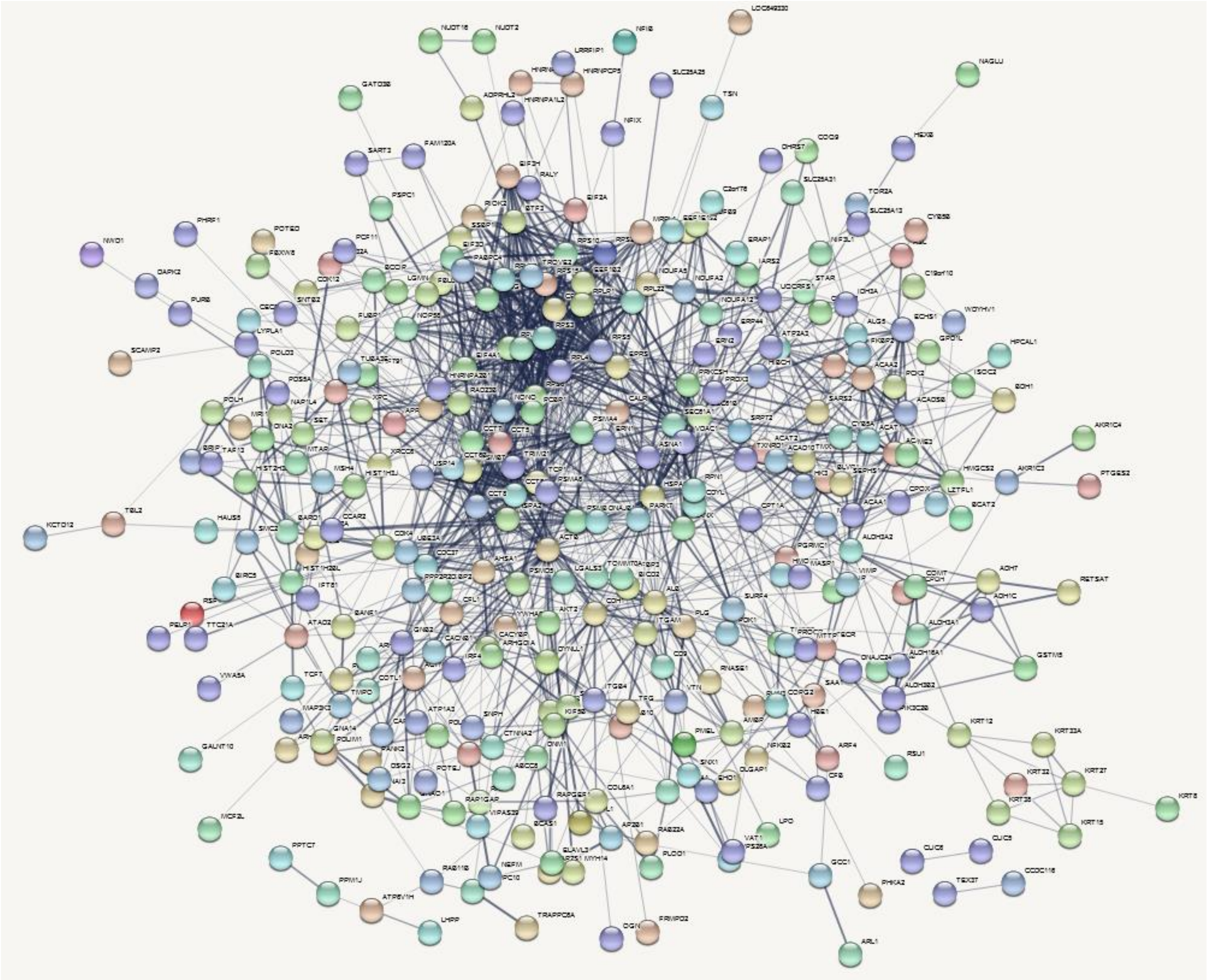

Figure S9: String network of shortlisted proteins downregulated in GC *vs.* NGM.

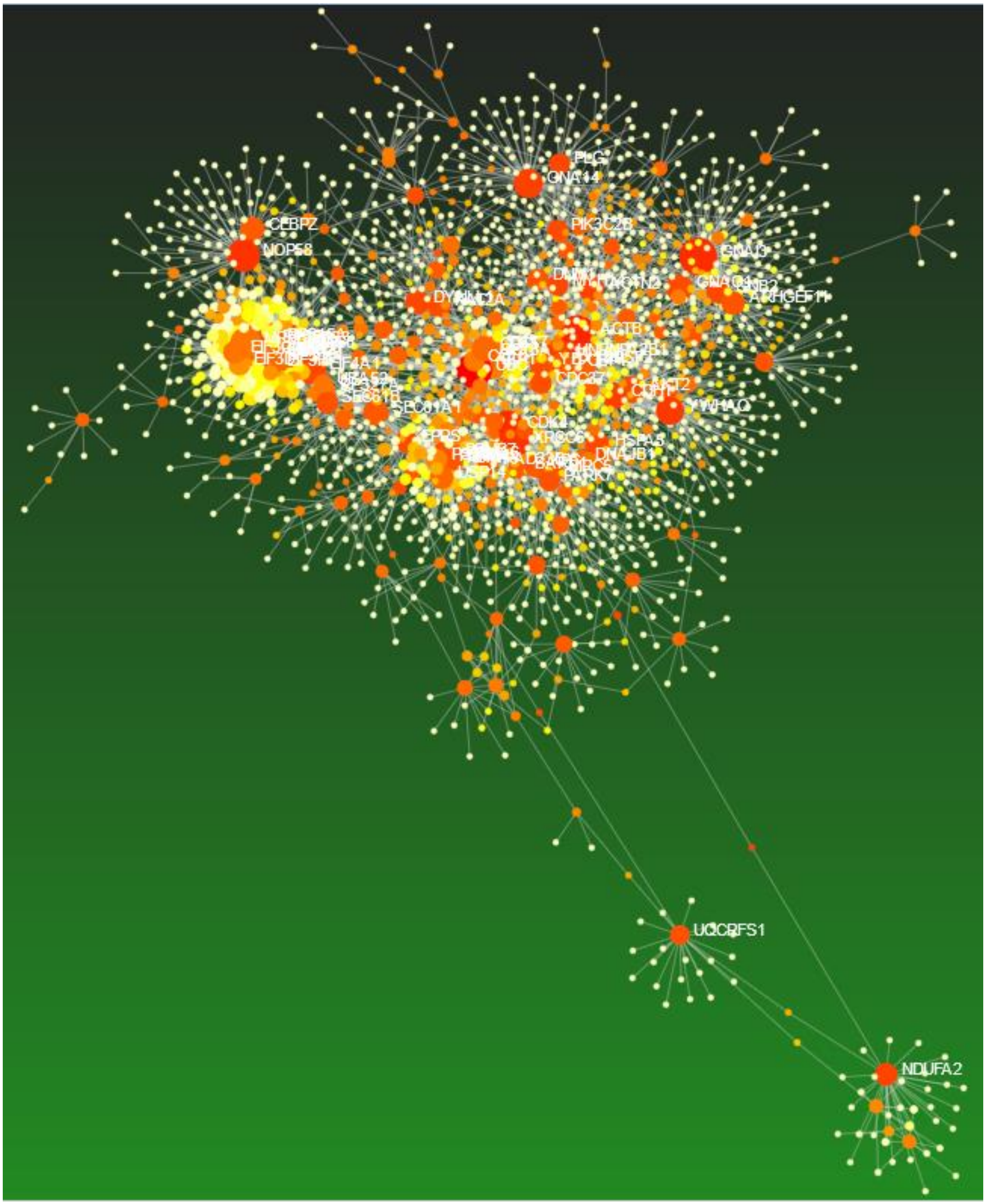

Figure S10: Network (string, NetworkAnalyst) of shortlisted proteins downregulated in GC *vs.* NGM.

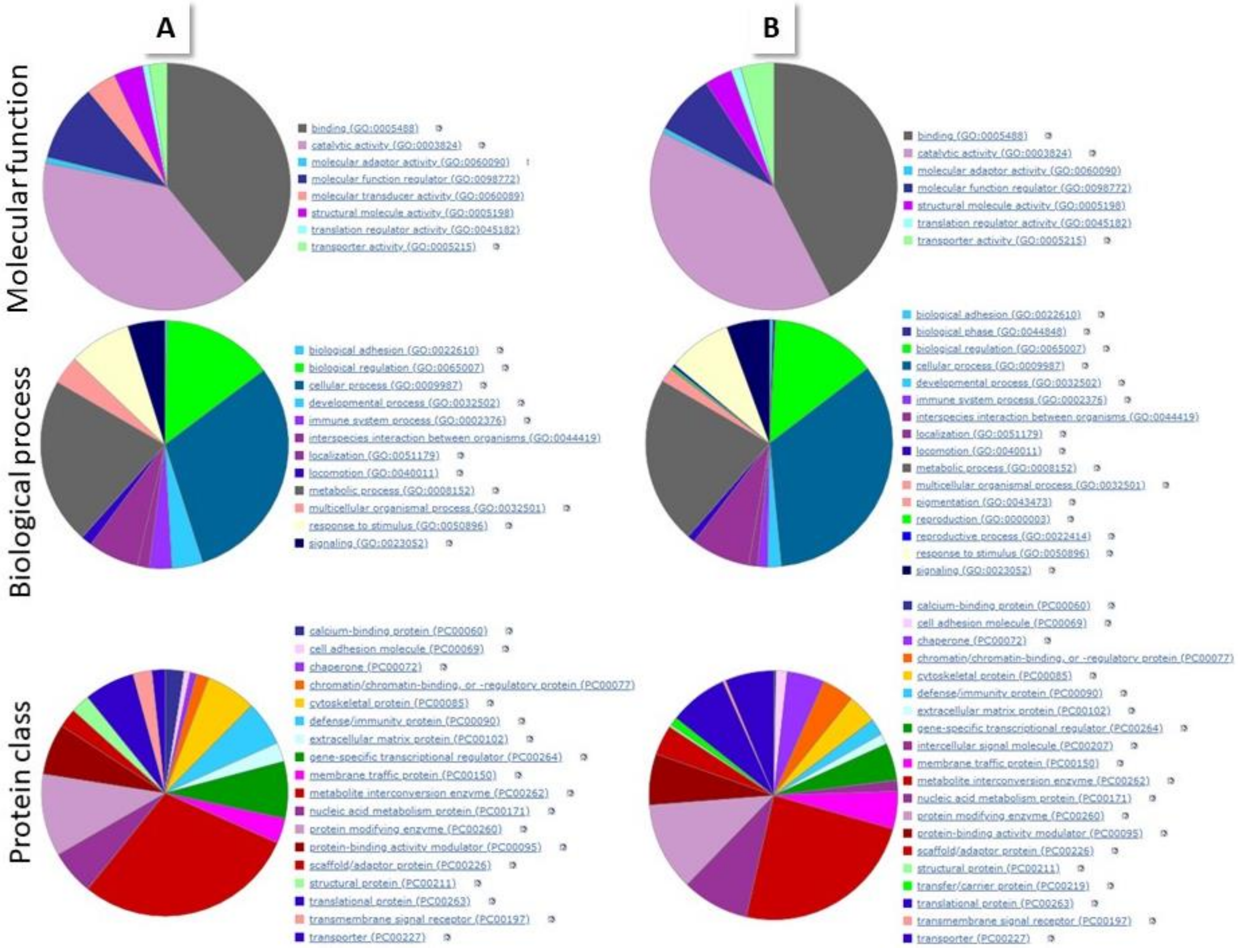

Figure S11: Functional classification analysis (Panther DB) of shortlisted proteins A) up- and B) downregulated in GC vs. NGM.

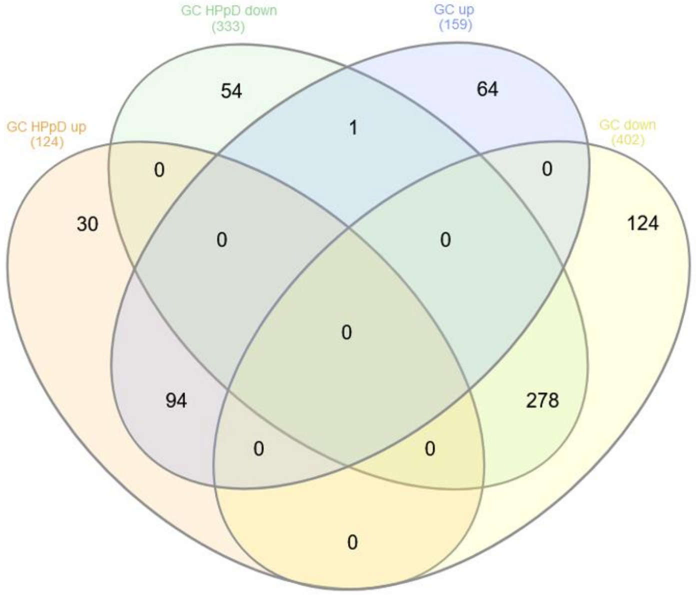

Figure S12: Venn diagram of shortlisted proteins for comparisons GC vs. NGM and HPpD GC *vs.* NGM.

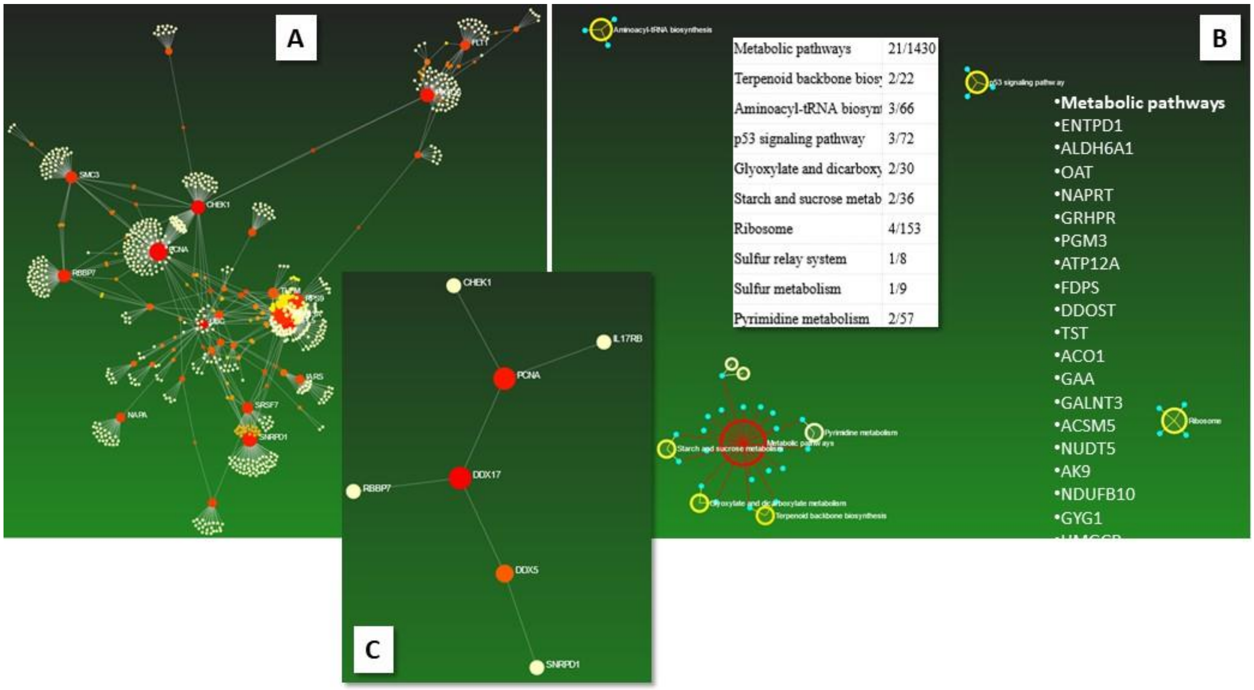

Figure S13: Network analysis (NetworkAnalyst) of shortlisted proteins upregulated in GC *vs.* NGM HPpD. A) Protein network (string), B) Enrichment ORA network, C) Zero-order network.

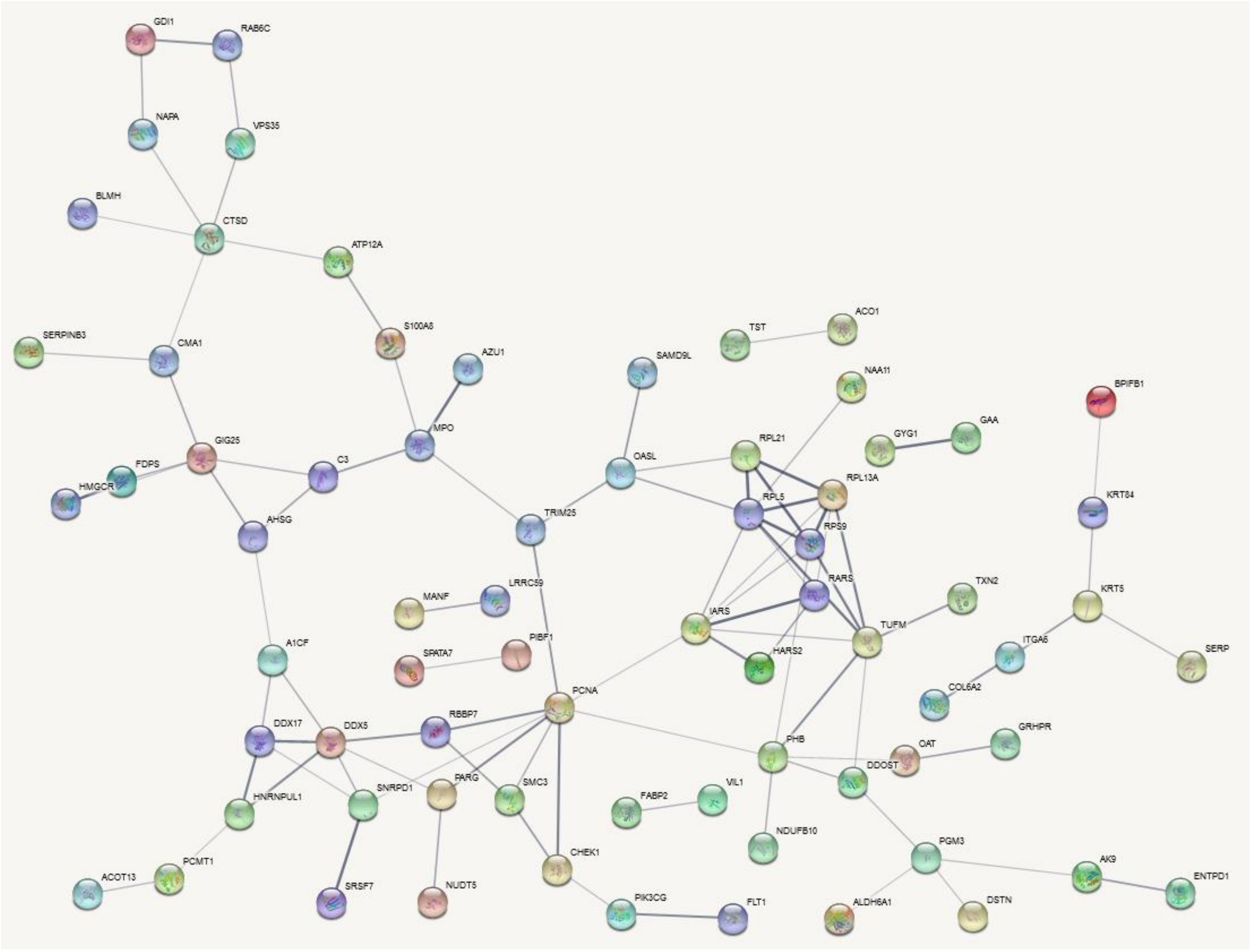

Figure S14: String analysis of shortlisted proteins upregulated in GC vs. NGM HPpD.

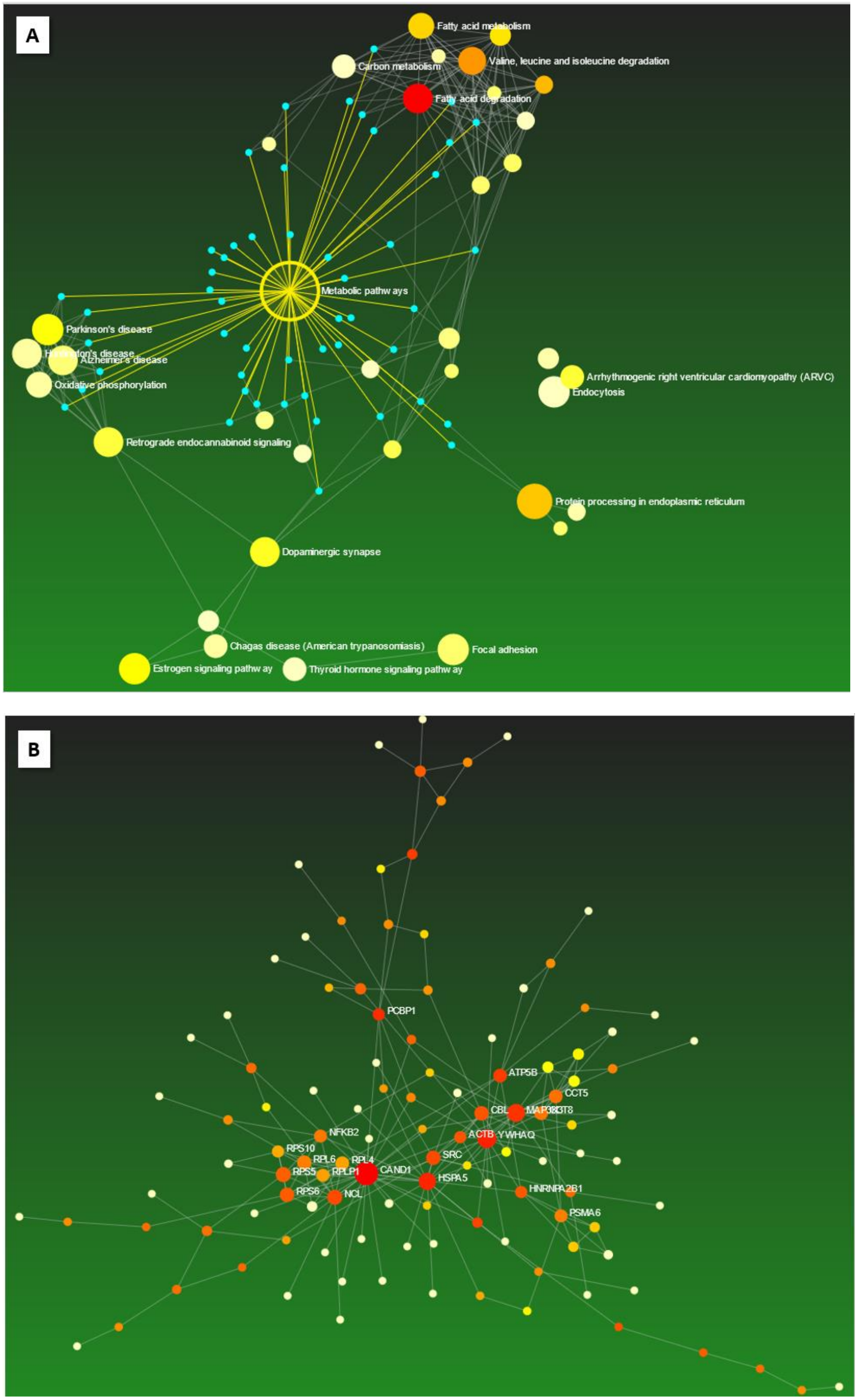

Figure S15: Network analysis (NetworkAnalyst) of shortlisted proteins downregulated in GC vs. NGM HPpD. A) Enrichment ORA network, B) Zero-order network.

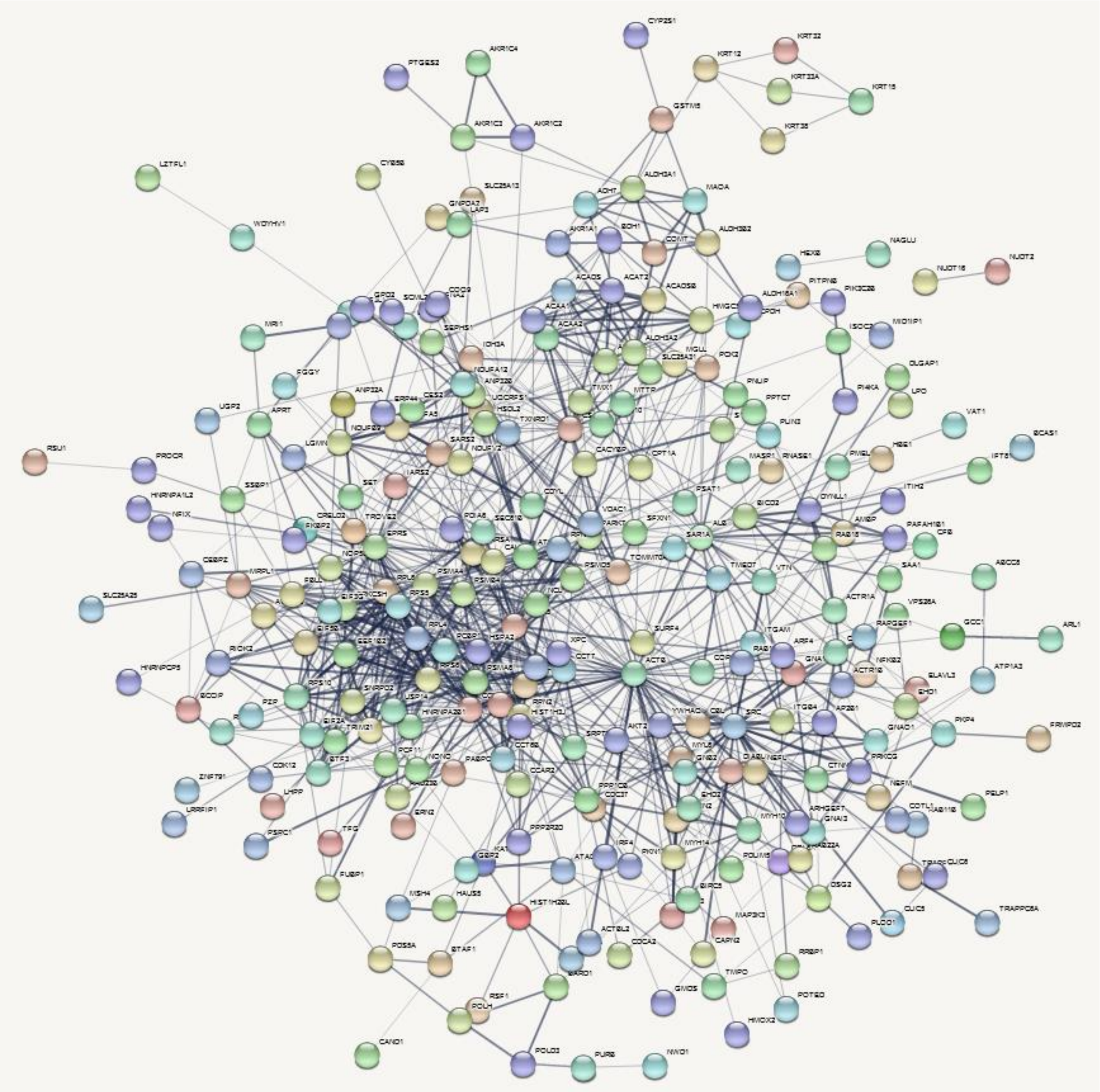

Figure S16: String analysis of shortlisted proteins downregulated in GC vs. NGM HPpD.

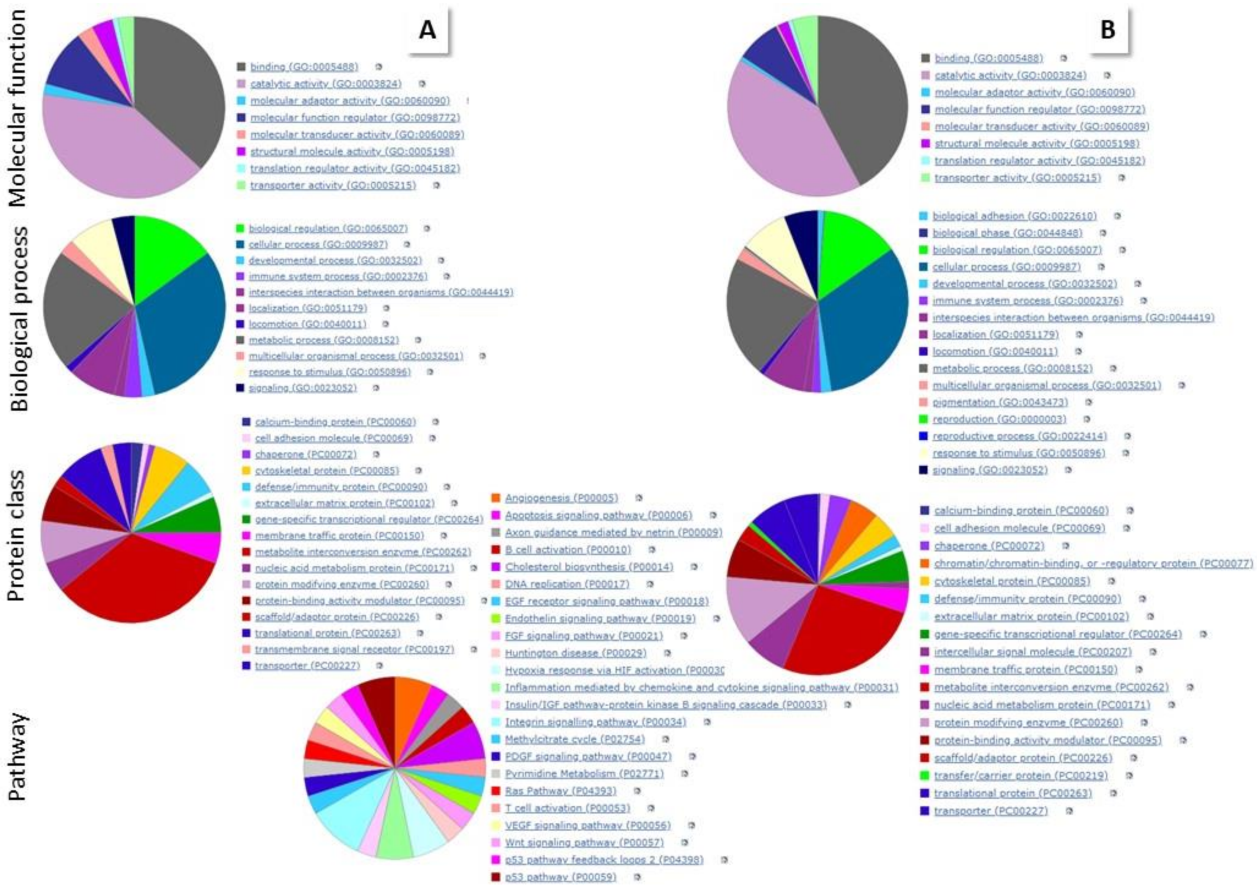

Figure S17: Functional classification analysis (Panther DB) of shortlisted proteins A) up- and B) downregulated in HPpD GC vs. NGM.

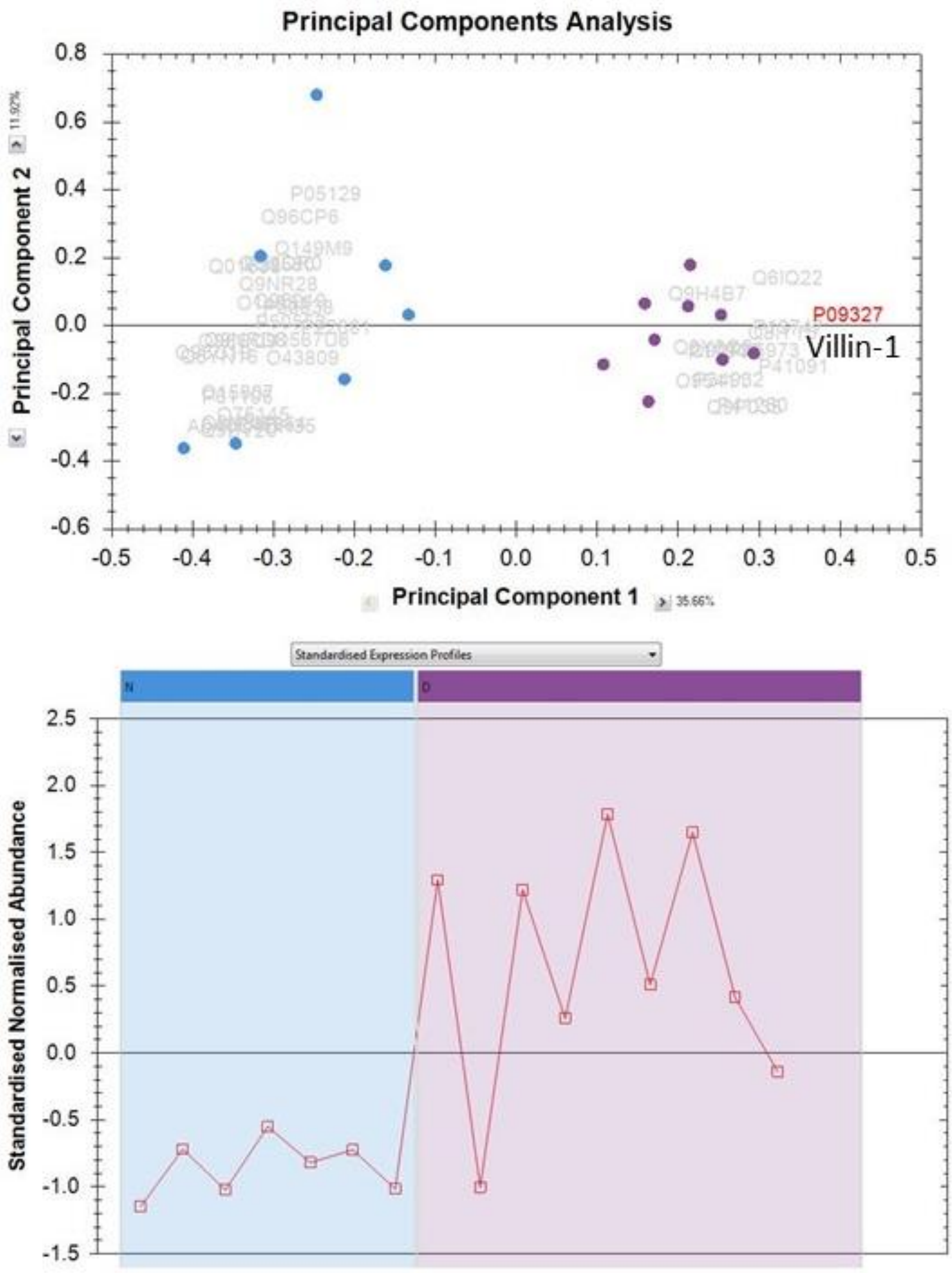

Figure S18: PCA of samples of diseased and normal sites in the same patients with GC HPp and the expression profile of one of the most abundant proteins in the set (VIL1).

Table S2: Upregulated shortlisted proteins in GC HPpD, sorted by the number of detected peptides and the fold value *vs.* N site.

**Accession**	**Peptide count**	**Max fold change**	**Description**	**Name**
P34932	82	2,1	Heat shock 70 kDa protein 4	HSPA4
P09327	50	3,2	Villin-1	VIL1
P11047	34	3,6	Laminin subunit gamma-1	LAMC1
P41091	34	8,4	Eukaryotic translation initiation factor 2 subunit 3	EIF2S3
Q9H4B7	28	2,1	Tubulin beta-1 chain	TUBB1
P41250	24	2,4	Glycine--tRNA ligase	GARS1
Q6IQ22	20	2,5	Ras-related protein Rab-12	RAB12
Q9Y6Q5	19	19,9	AP-1 complex subunit mu-2	AP1M2
Q8IYT3	14	2,8	Coiled-coil domain-containing protein 170	CCDC170
P13747	14	43,4	HLA class I histocompatibility antigen_ alpha chain E	HLA-E
Q9P035	6	2,5	Very-long-chain (3R)-3-hydroxyacyl-CoA dehydratase 3	HACD3
Q92738	5	2,8	USP6 N-terminal-like protein	USP6NL
O95411	3	2,6	TGFB1-induced anti-apoptotic factor 1	TIAF1
P45973	3	3,4	Chromobox protein homolog 5	CBX5

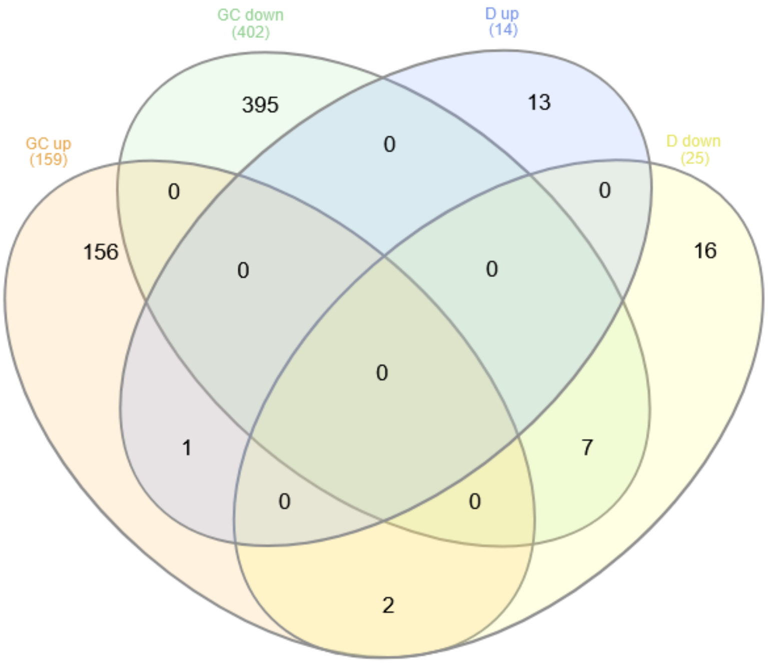

**D down GC up**	**D down GC down**	**D up GC up**
Q01538	P30838	P09327
Q96CP6	Q15907	
	Q149M9	
	P45954	
	A0A0C4DH35	
	Q9BV20	
	Q8N9M1	

Figure S19: Venn diagram and shared proteins of shortlisted proteins of samples of D and N sites in the same patients with GC HPp *vs.* NGM comparison.

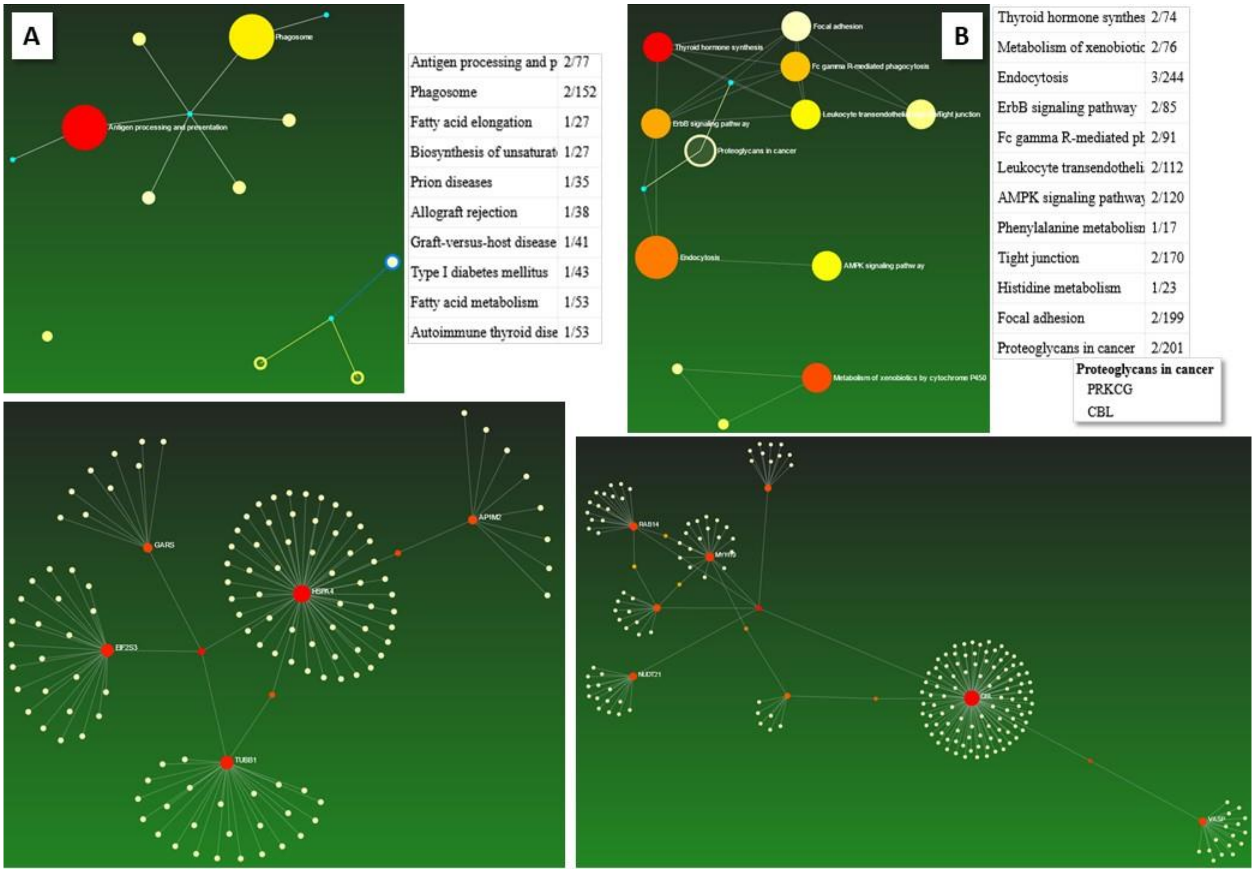

Figure S20: Network analysis (NetworkAnalyst) of samples of diseased and normal sites in the same patients with GC HPp. A) Shortlisted proteins up-, B) downregulated in D.

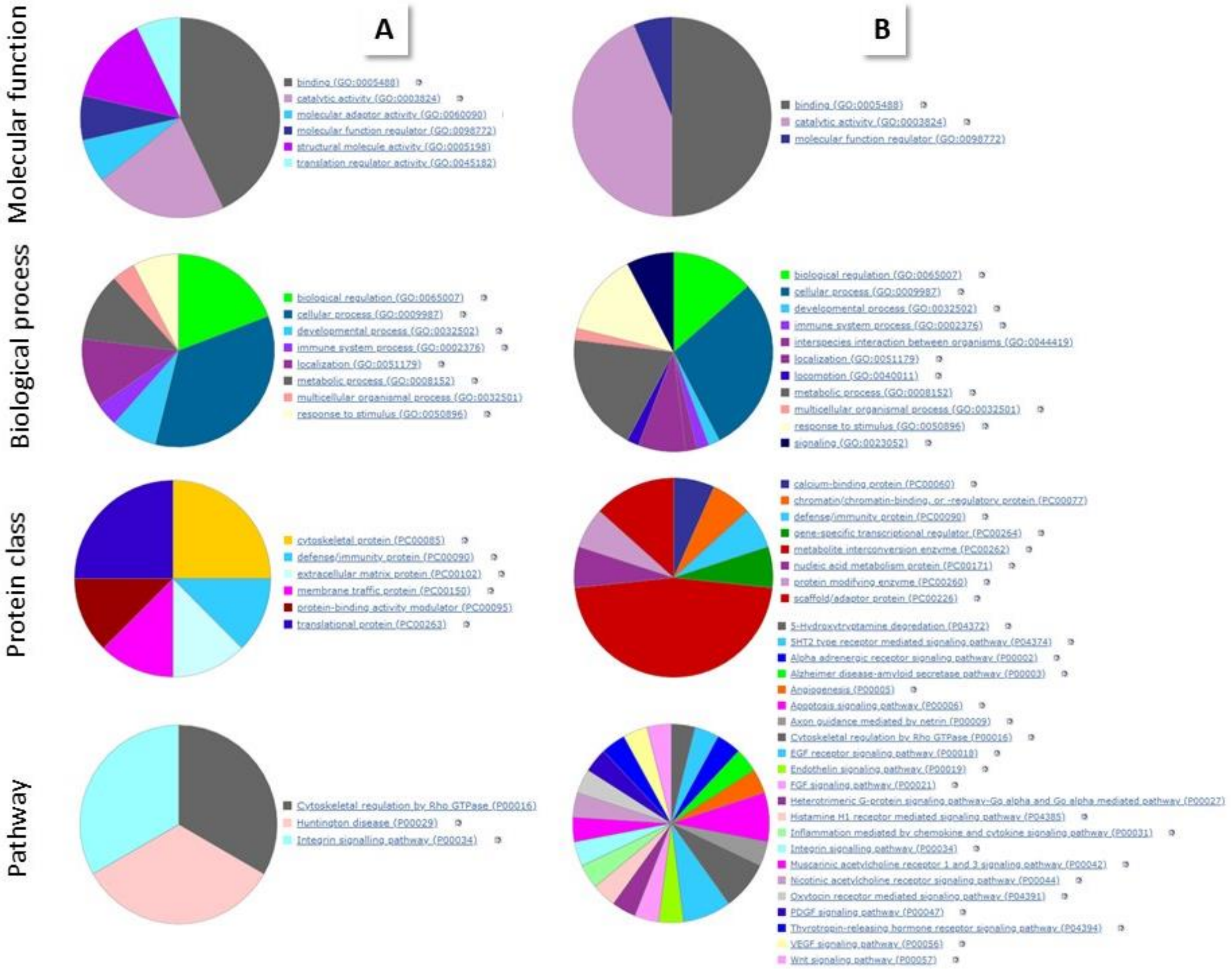

Figure S21: Functional classification analysis (Panther DB) of shortlisted proteins of samples of diseased and normal sites in the same patients with GC HPp. A) Proteins up-, B) downregulated in D.

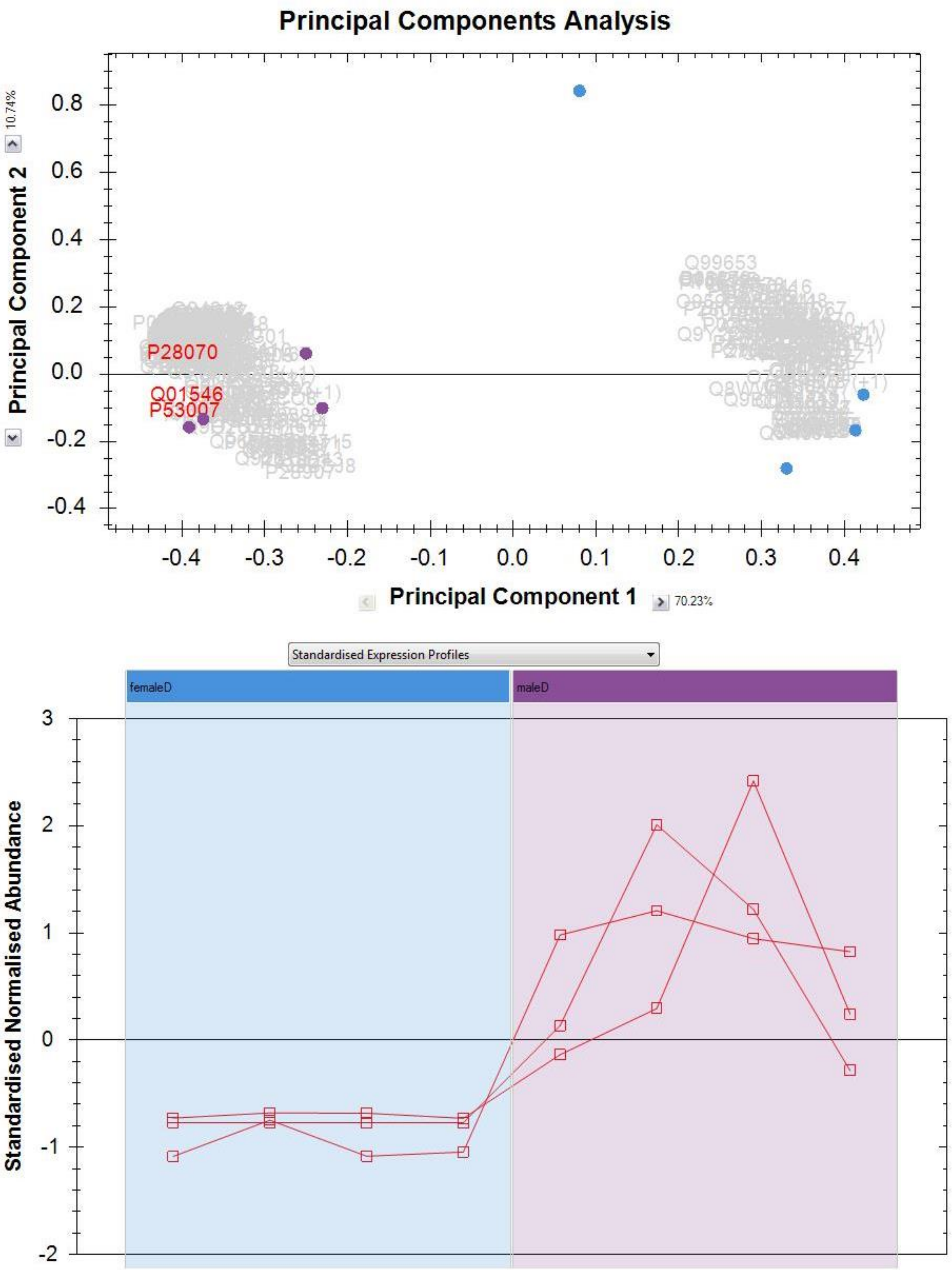

Figure S22: PCA of shortlisted proteins from samples of female and male GC patients (HPp) and three characteristic expression profiles (Q01546 - KRT76, P53007 – SLC25A1, P28070 – PSMB4).

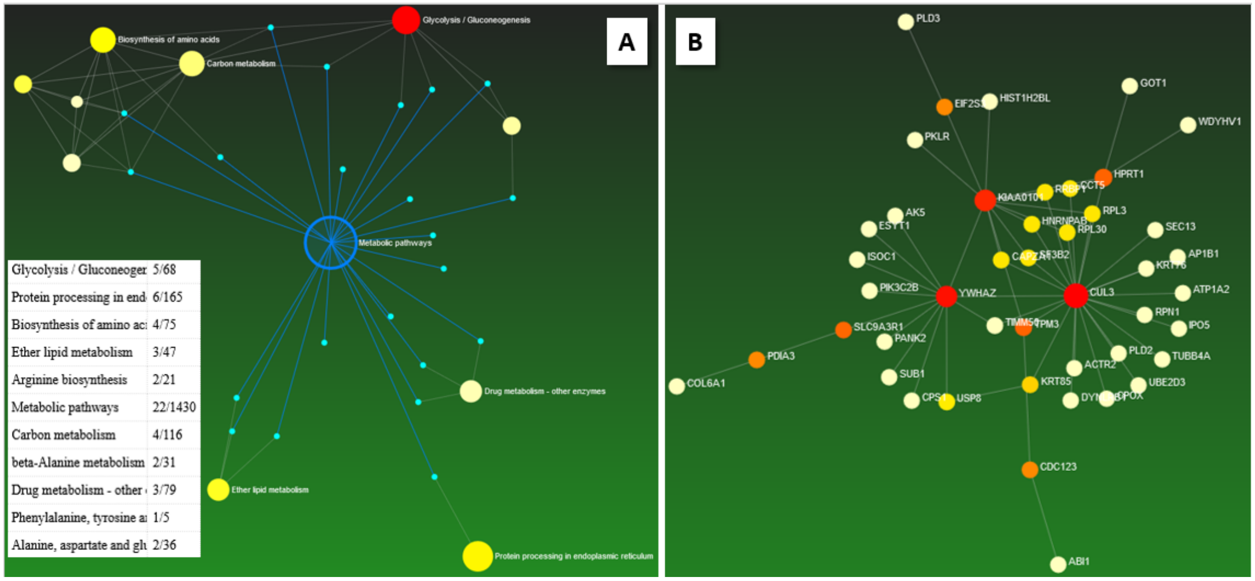

Figure S23: Network analysis (NetworkAnalyst) of shortlisted proteins upregulated in GC HPpD males vs. females. A) Enrichment ORA network, B) Zero-order network.

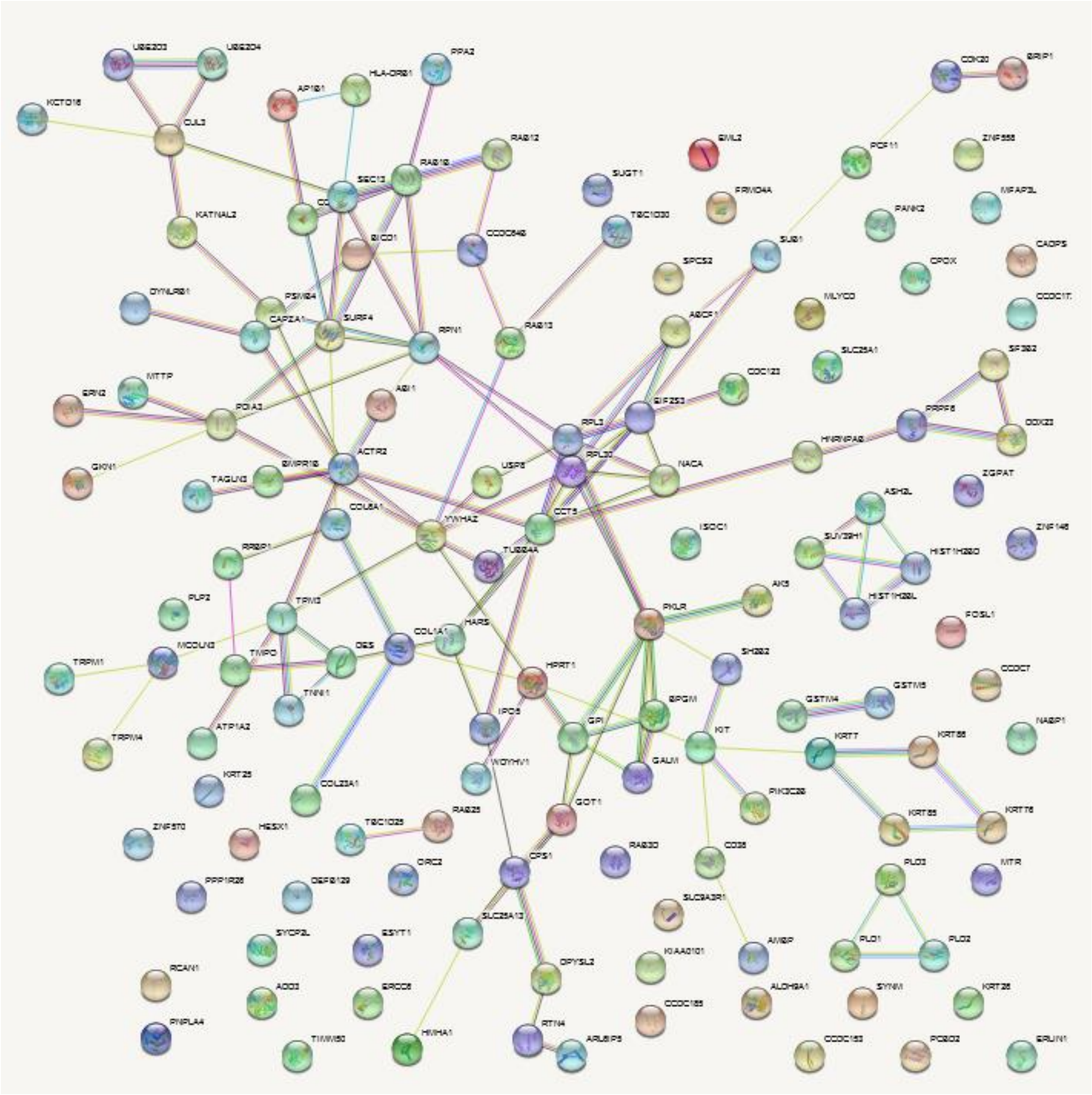

Figure S24: String network of shortlisted proteins upregulated in GC HPpD males *vs.* females.

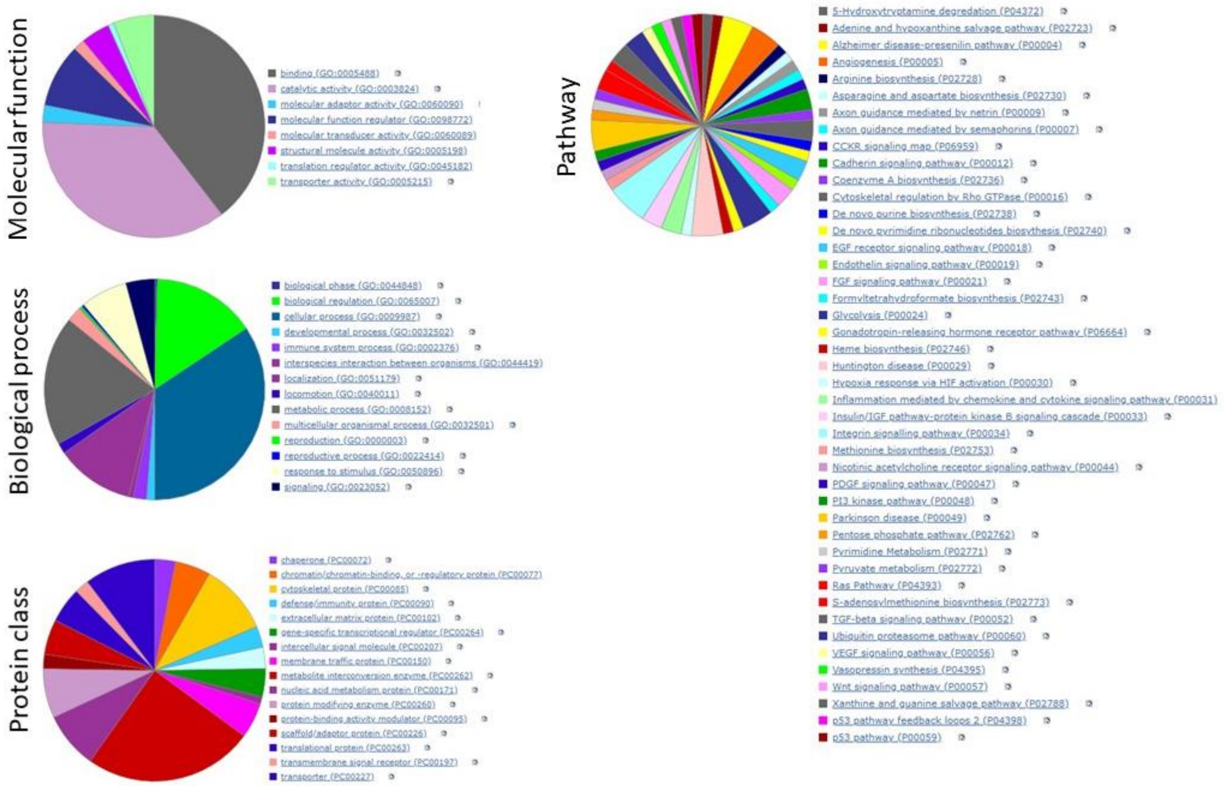

Figure S25: Functional classification of shortlisted proteins upregulated in GC HPpD males *vs.* females.

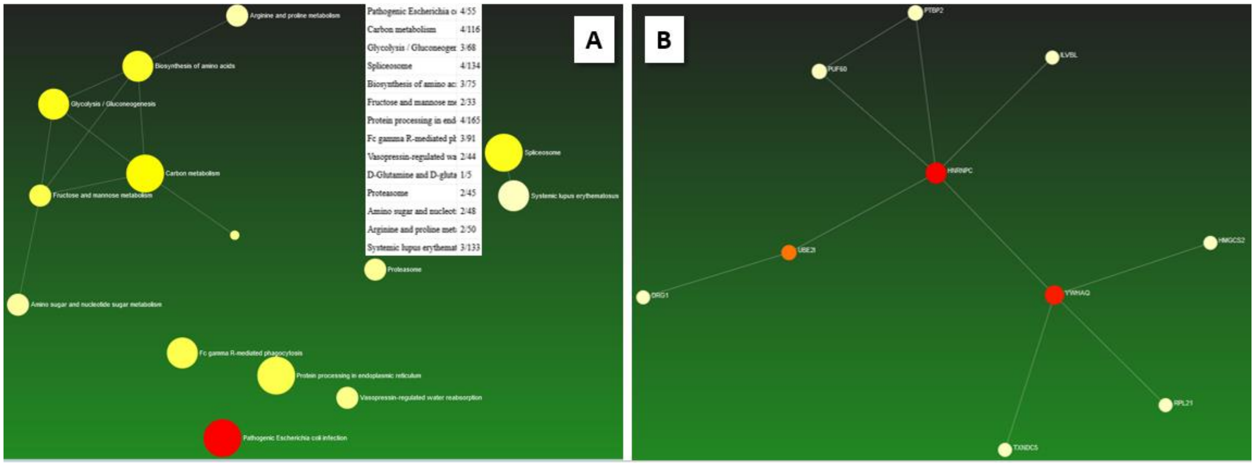

Figure S26: Network analysis (NetworkAnalyst) of shortlisted proteins upregulated in GC HPpD females *vs.* males. A) Enrichment ORA network, B) Zero-order network.

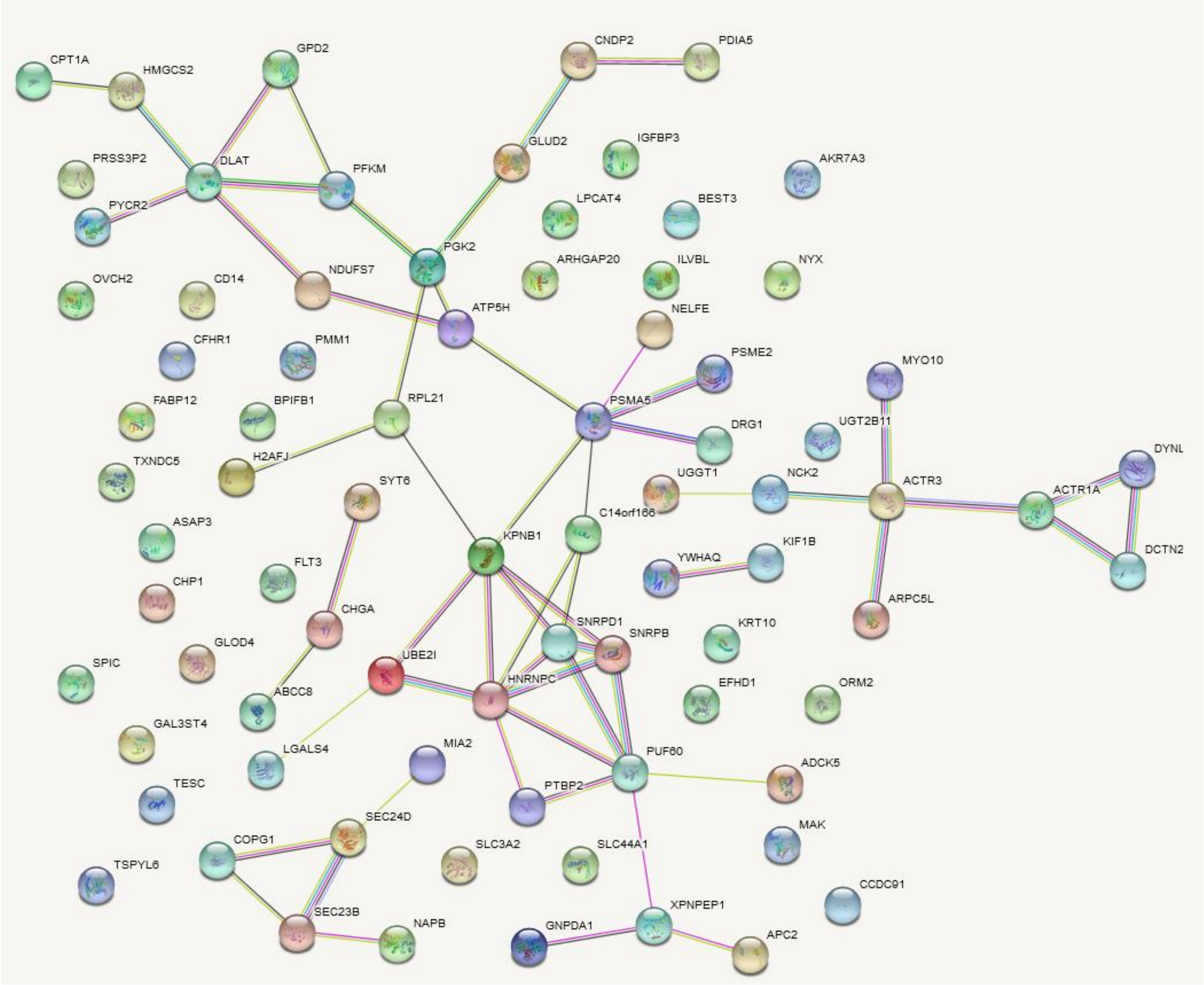

Figure S27: String network of shortlisted proteins upregulated in GC HPpD females *vs.* males.

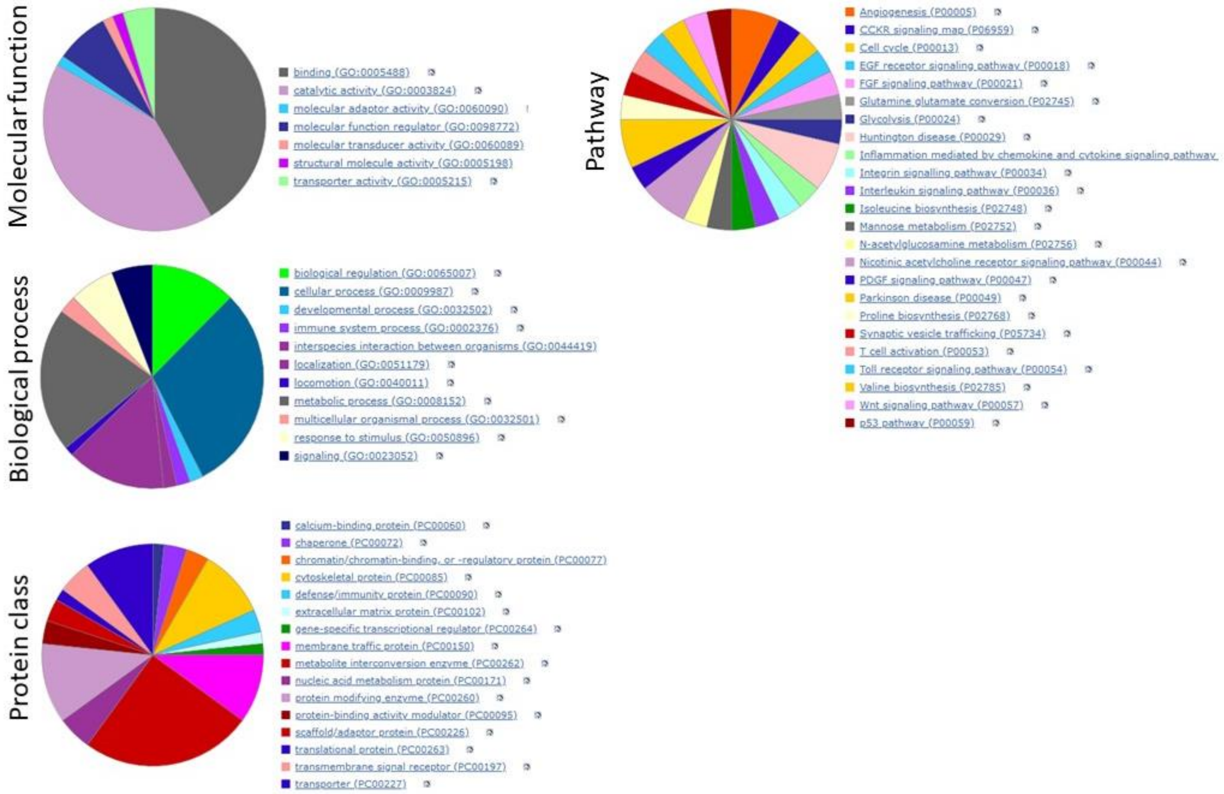

Figure S28: Functional classification of shortlisted proteins upregulated in GC HPpD females *vs.* males.

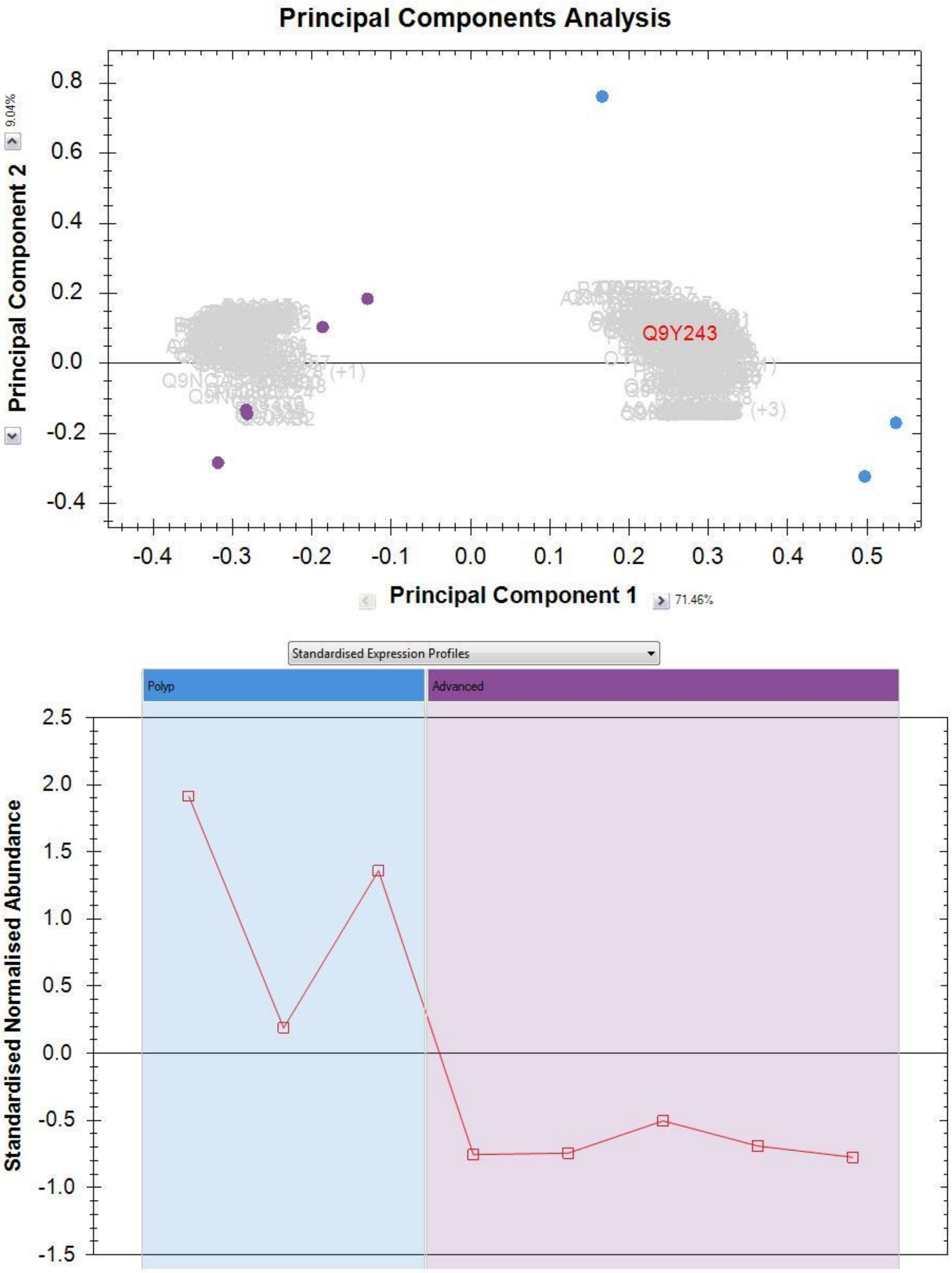

Figure S29: PCA HPpD 1st stage *vs.* advanced GC.

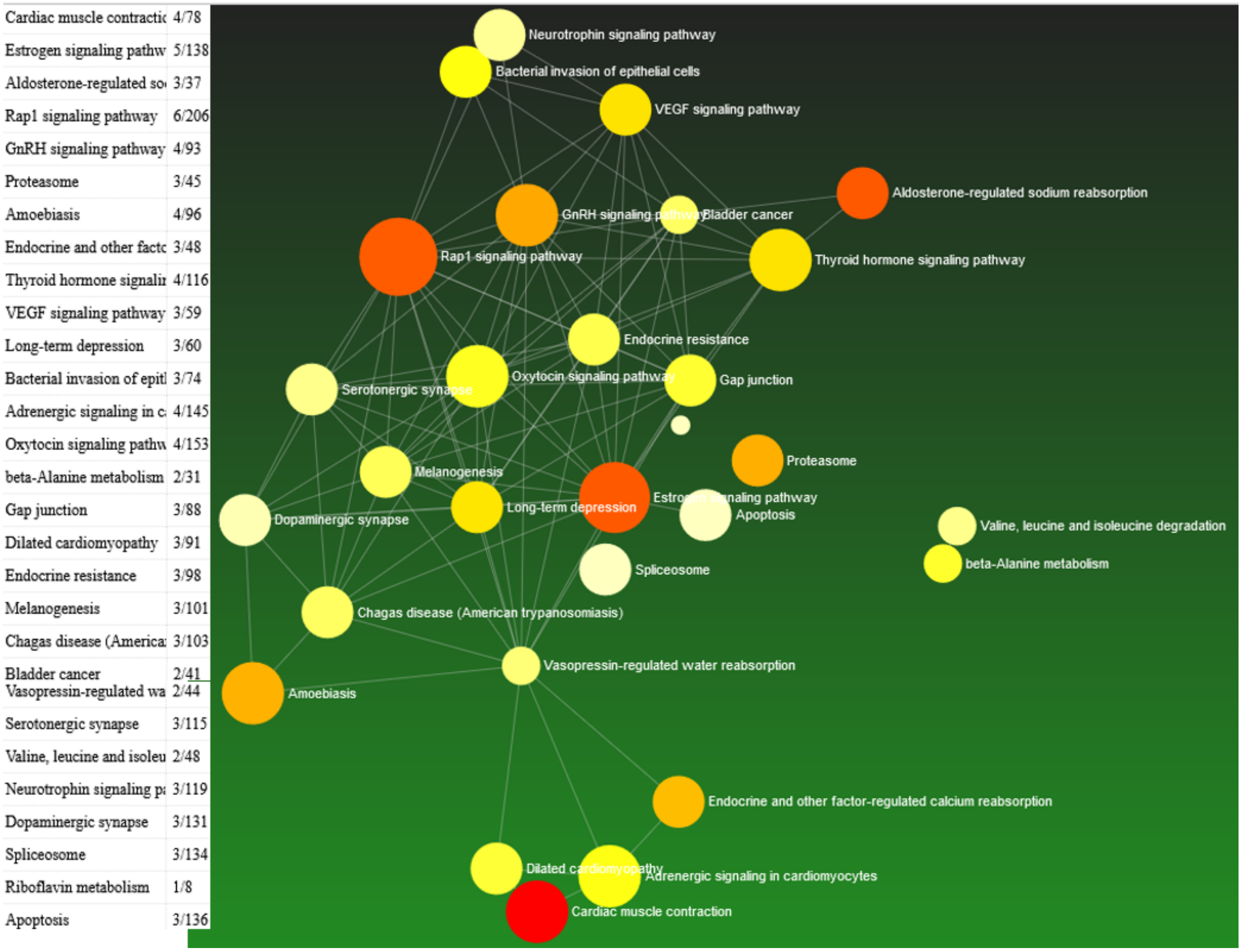

Figure S30: ORA network of shortlisted proteins upregulated in advanced GC HPpD *vs.* 1st stage GC.

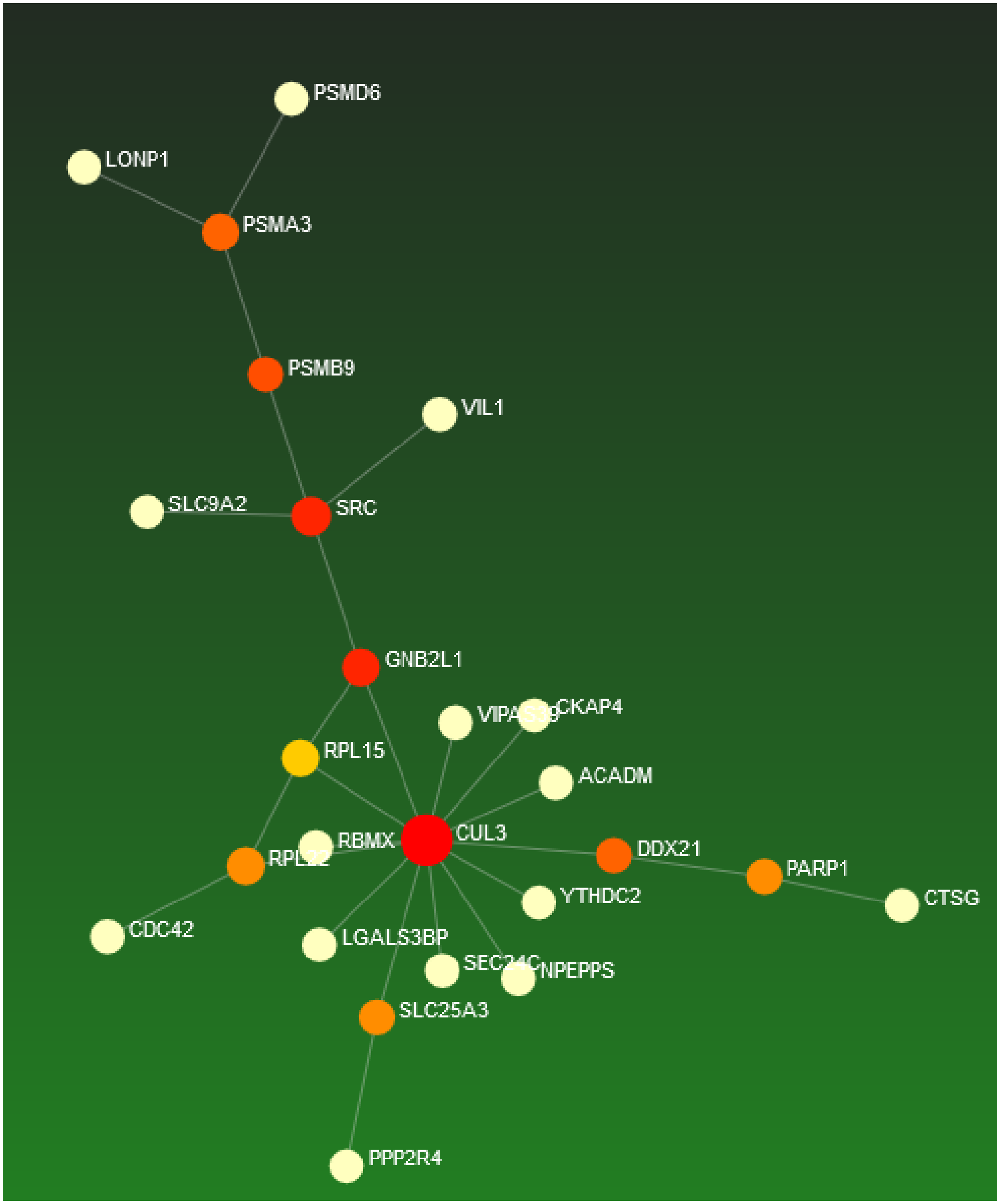

Figure S31: Zero order network of shortlisted proteins upregulated in advanced GC HPpD *vs.* 1st stage GC.

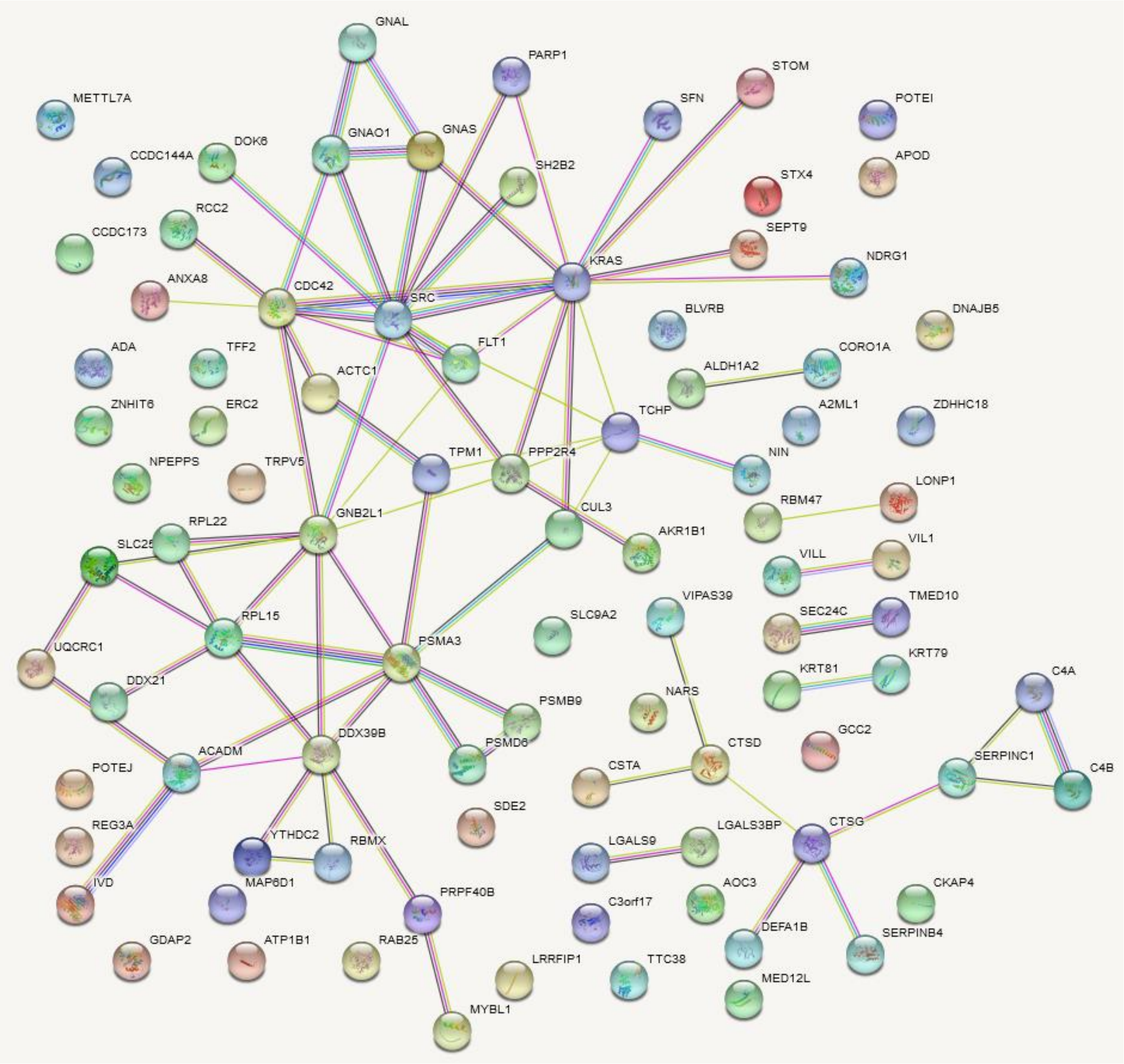

Figure S32: String network of shortlisted proteins upregulated in advanced GC HPpD *vs.* 1st stage GC.

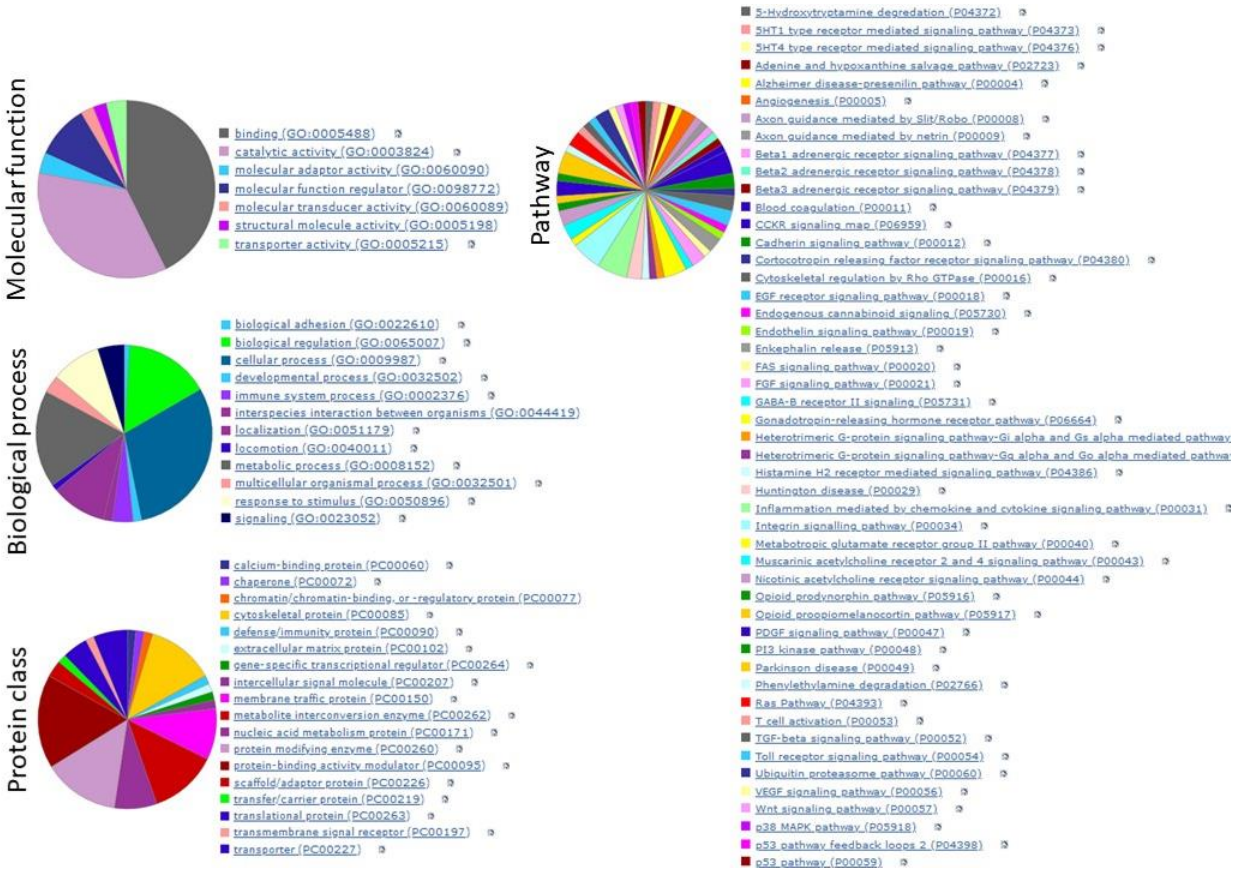

Figure S33: Functional classification ofshortlisted proteins upregulated in advanced GC HPpD *vs.* 1st stage GC.

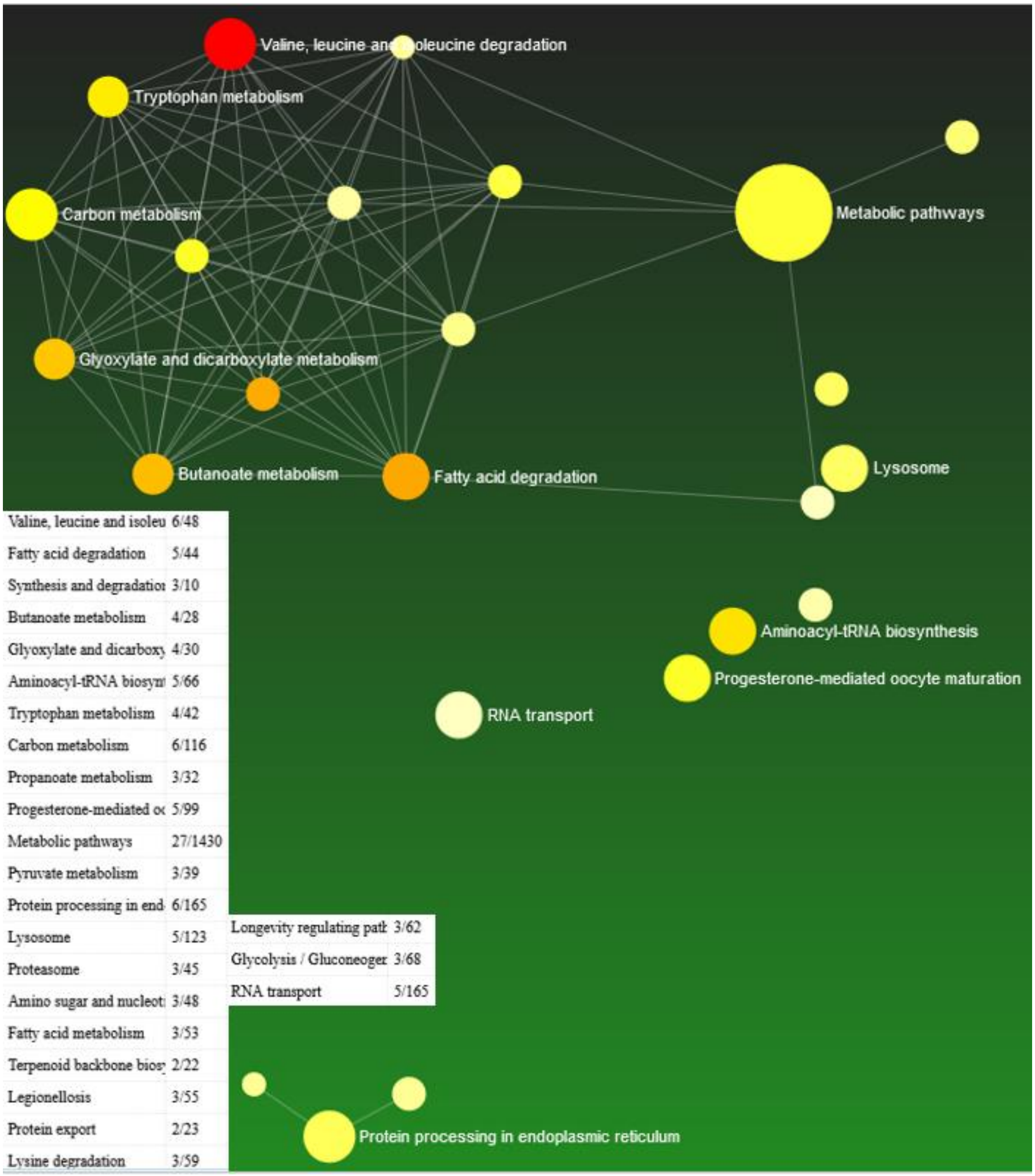

Figure S34: ORA network of shortlisted proteins downregulated in advanced GC HPpD *vs.* 1st stage GC.

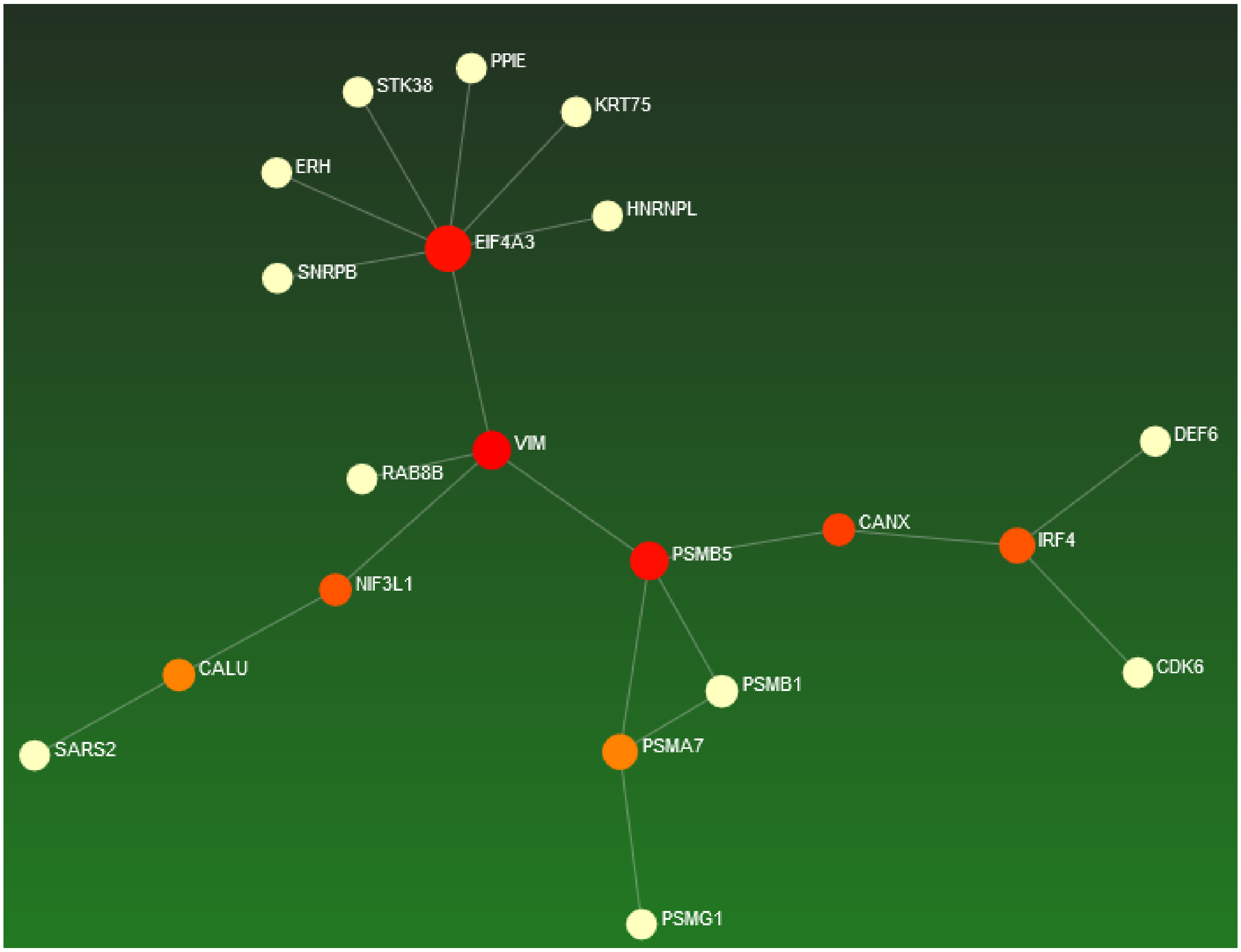

Figure S35: Zero order network of shortlisted proteins downregulated in advanced GC HPpD *vs.* 1st stage GC.

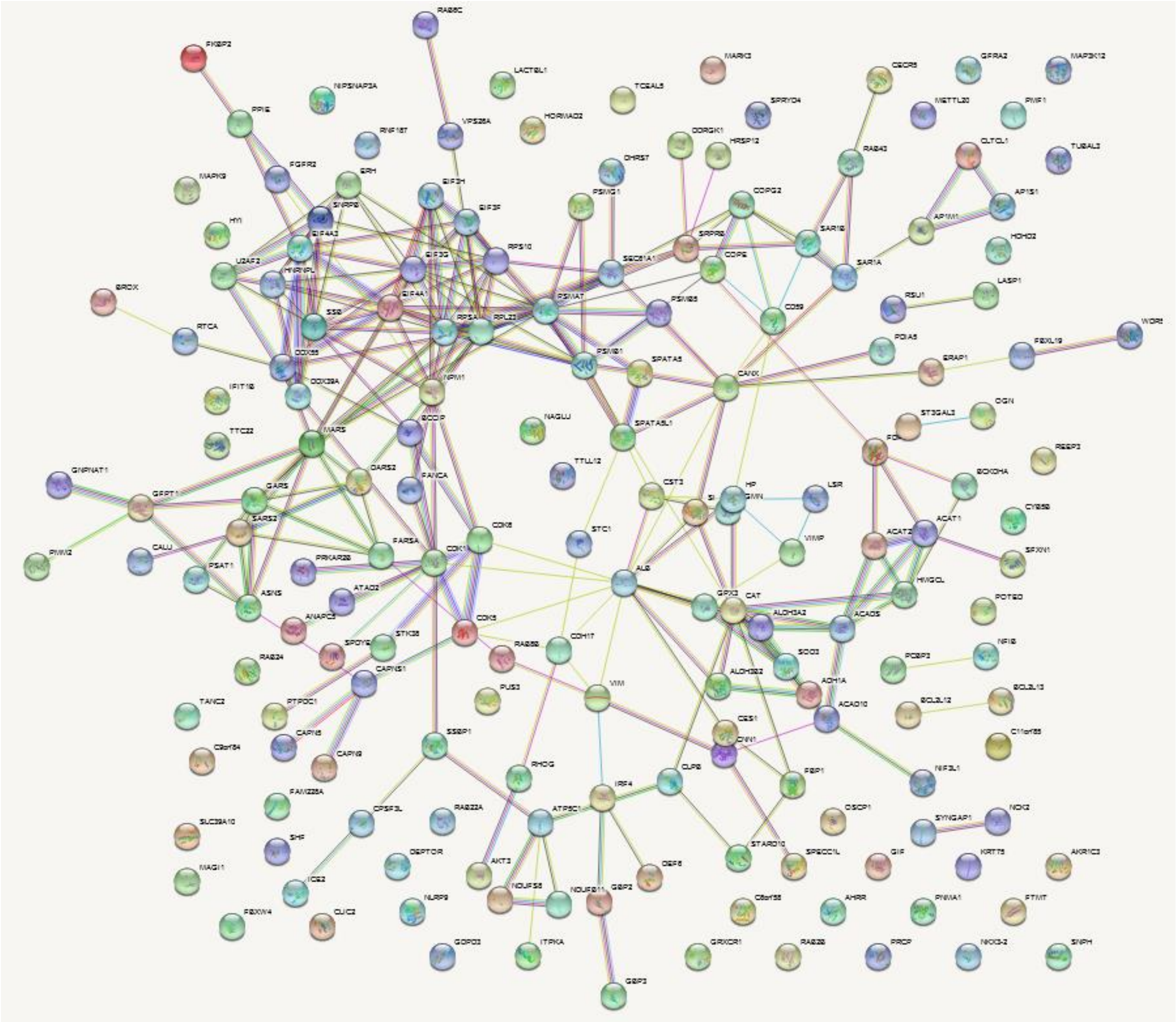

Figure S36: String network of shortlisted proteins downregulated in advanced GC HPpD *vs*. 1st stage GC.

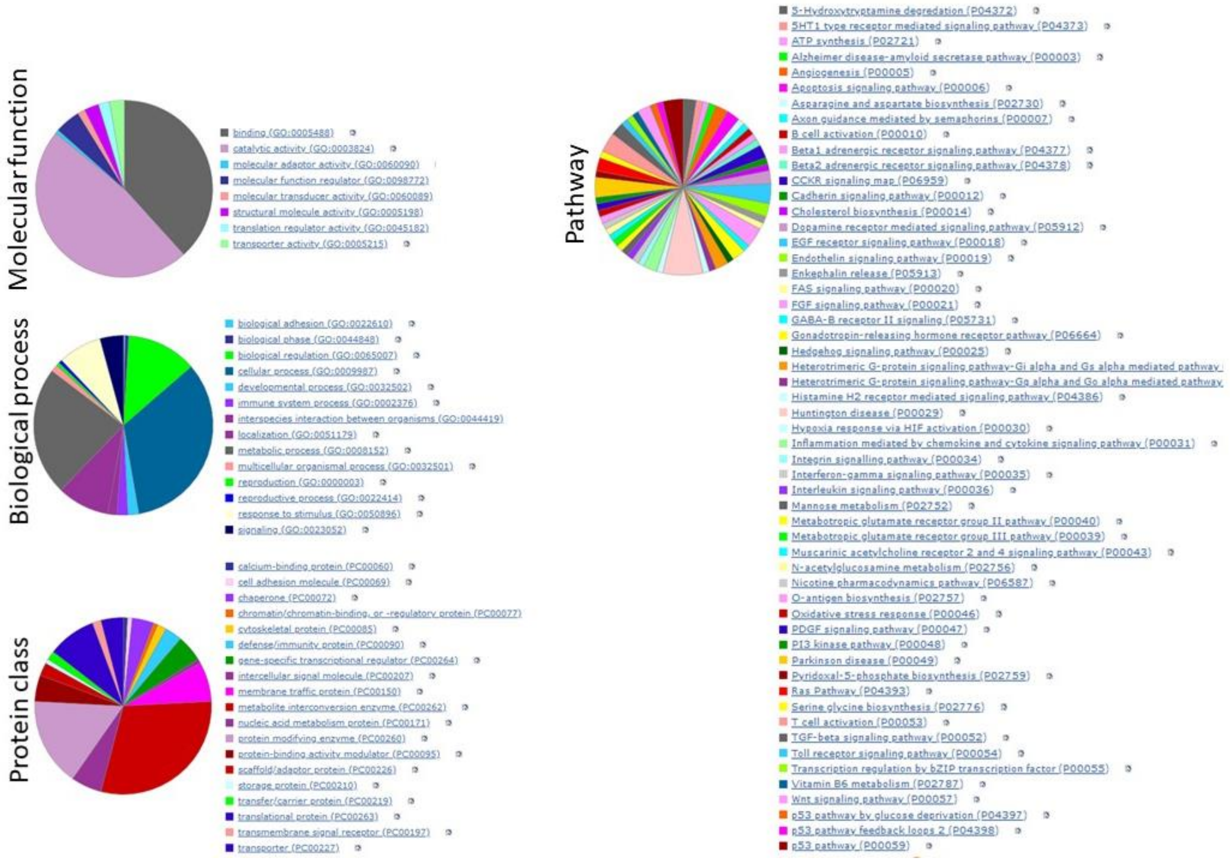

Figure S37: Functional classification ofshortlisted proteins downregulated in advanced GC HPpD *vs.* 1st stage GC.

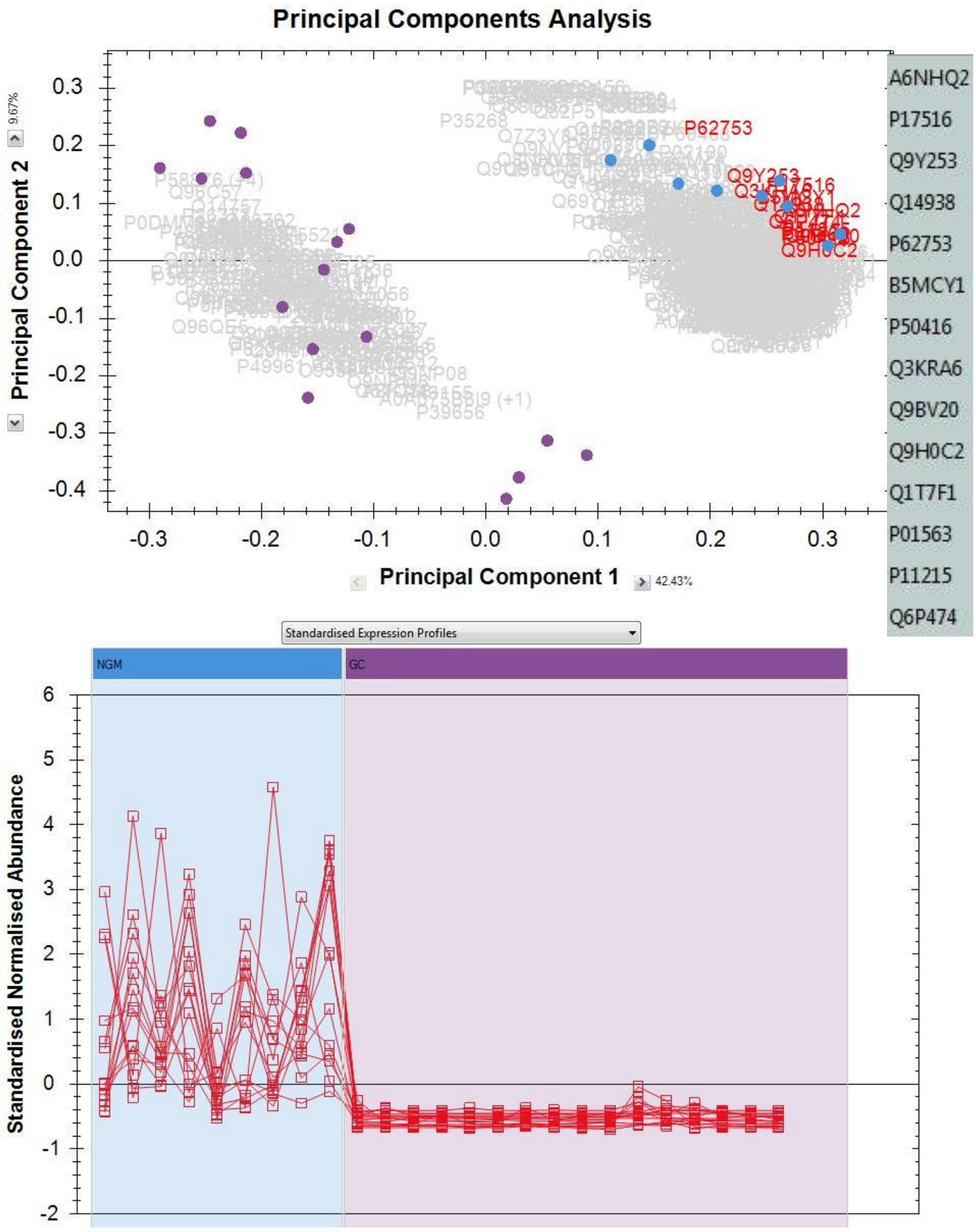

Figure S38: PCA for antrum NGM *vs.* GC.

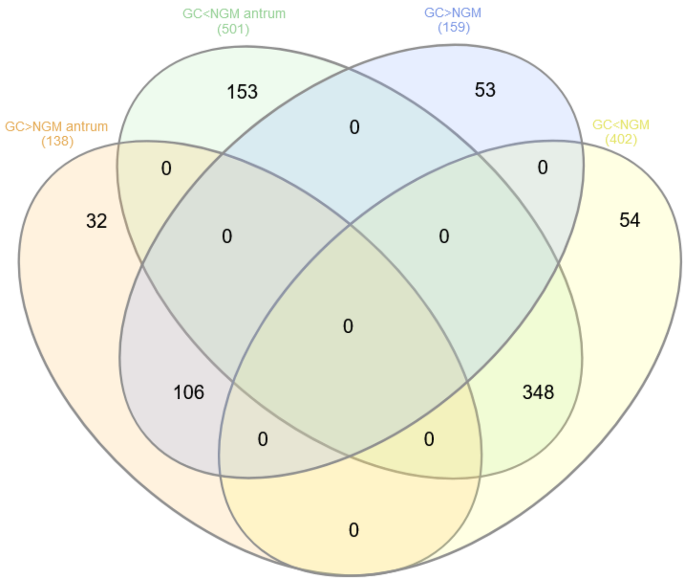

Figure S39: Venn diagram for the comparison of NGM (only antrum or all samples) vs. GC. See protein lists in Supplementary file "analysis".

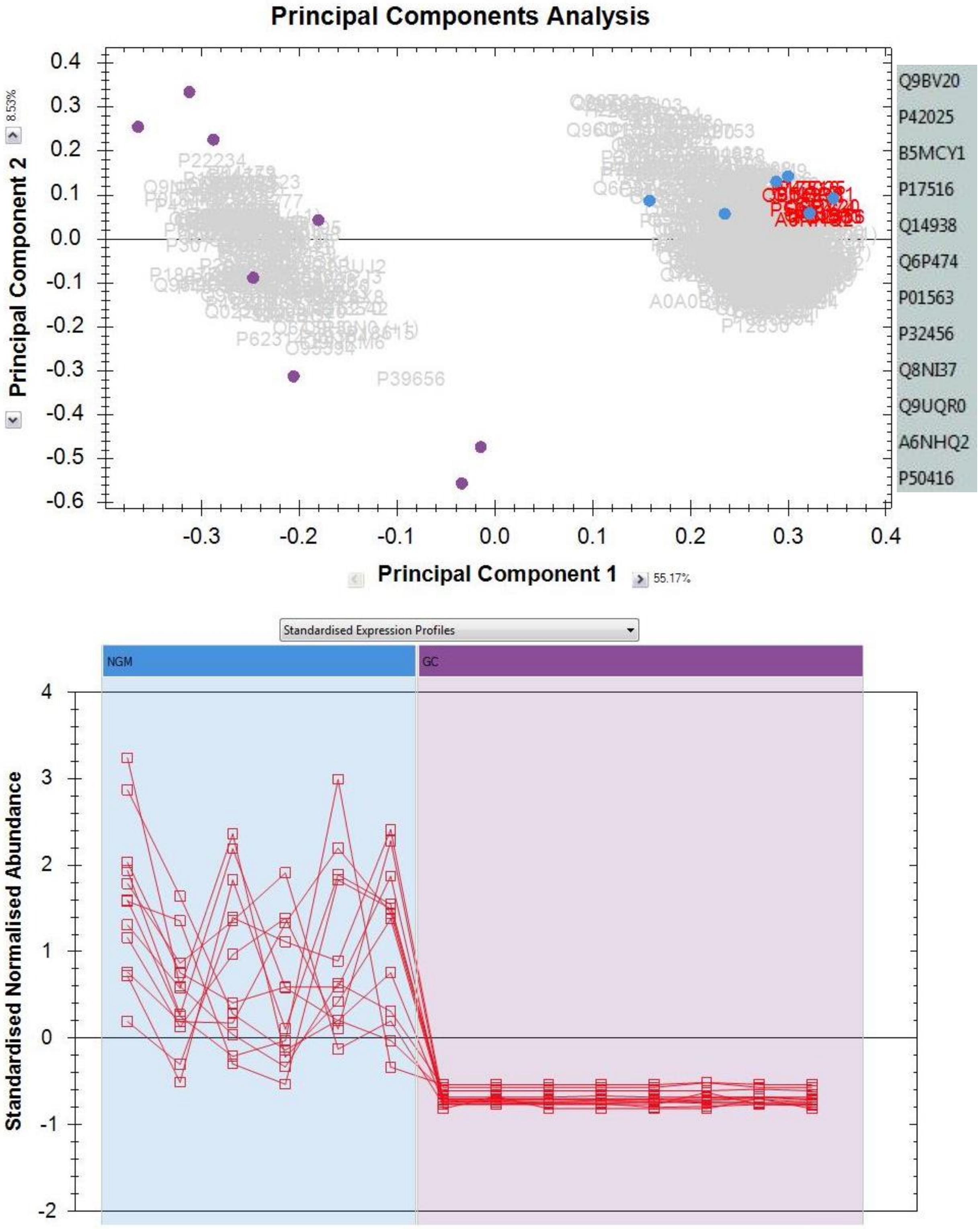

Figure S40: PCA for antrum NGM *vs.* GC HPpD and expression profiles for the proteins in the grey box.

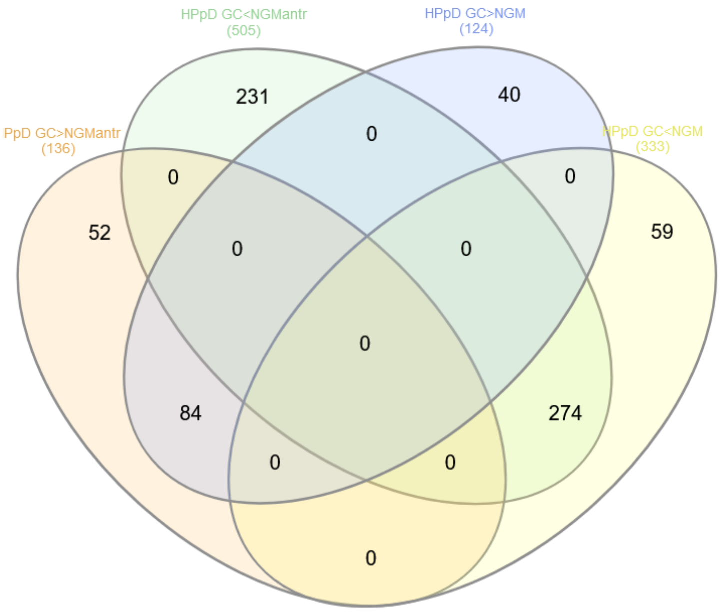

Figure S41: Venn diagram for the comparison of NGM (only antrum or all samples) *vs.* GC HPpD. See protein lists in Supplementary file "analysis".

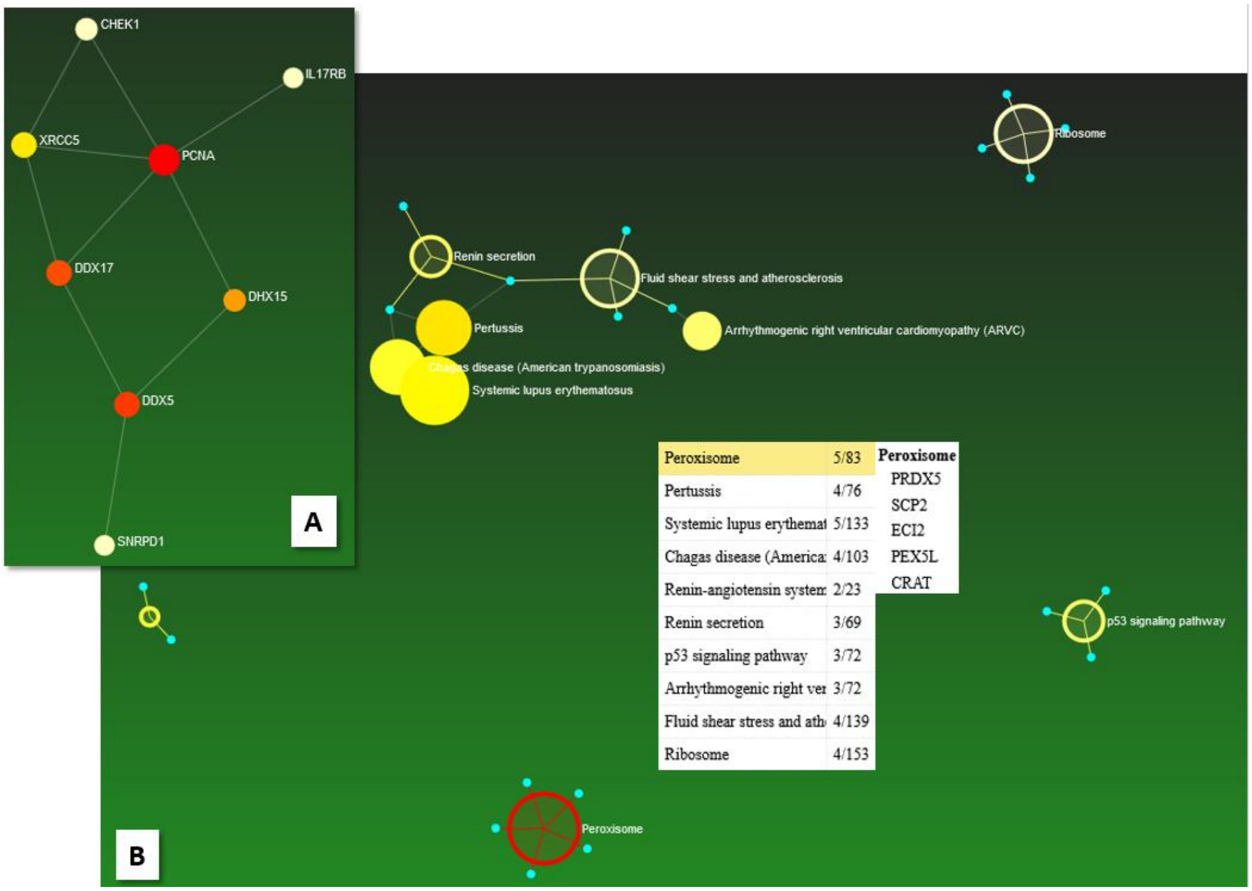

Figure S42: Network analysis (NetworkAnalyst) of shortlisted proteins upregulated in GC *vs.* NGM antrum. A) Zero-order network. B) Enrichment ORA network,

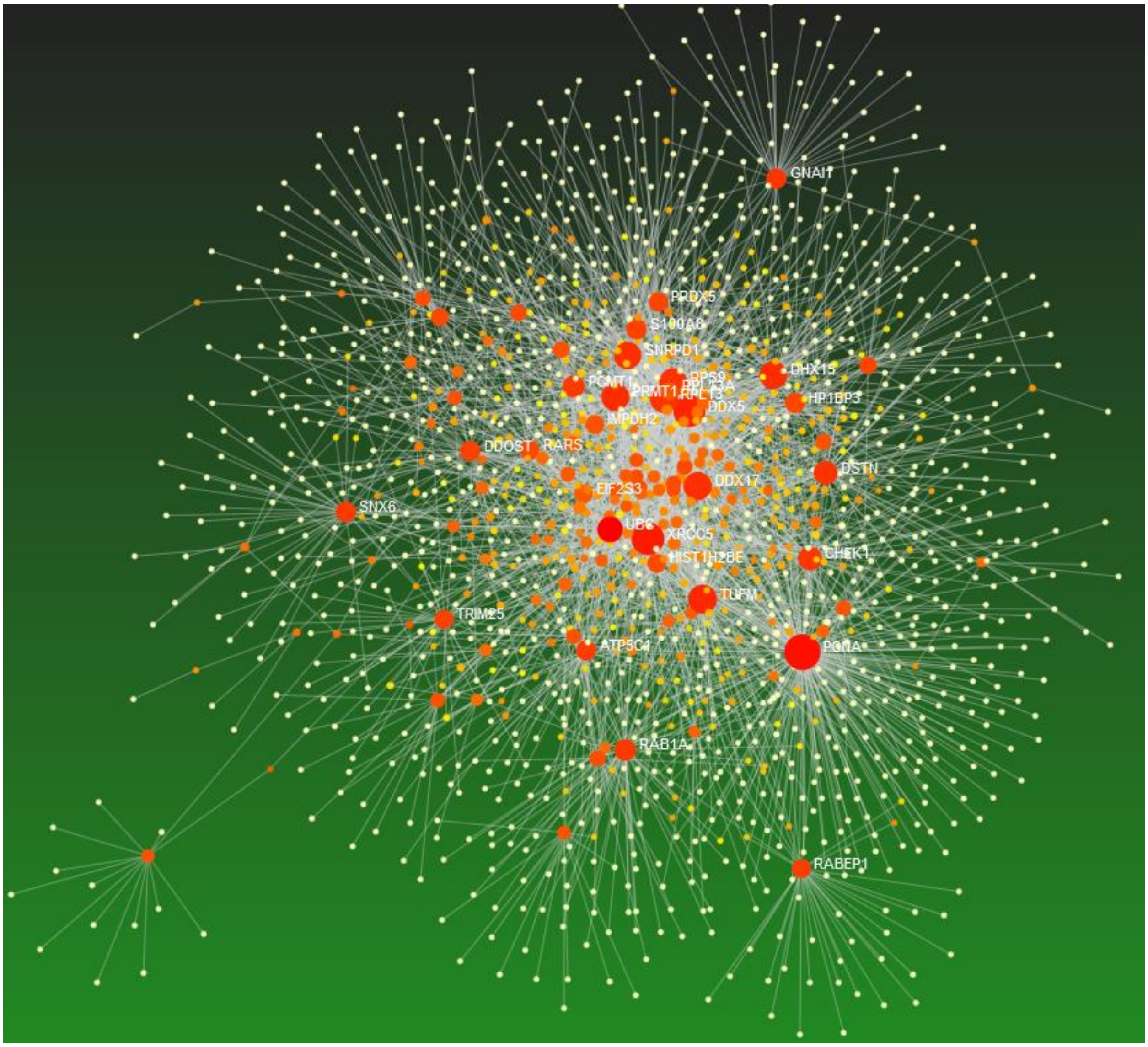

Figure S43: Network visualisation (NetworkAnalyst) of shortlisted proteins upregulated in GC *vs.* NGM antrum.

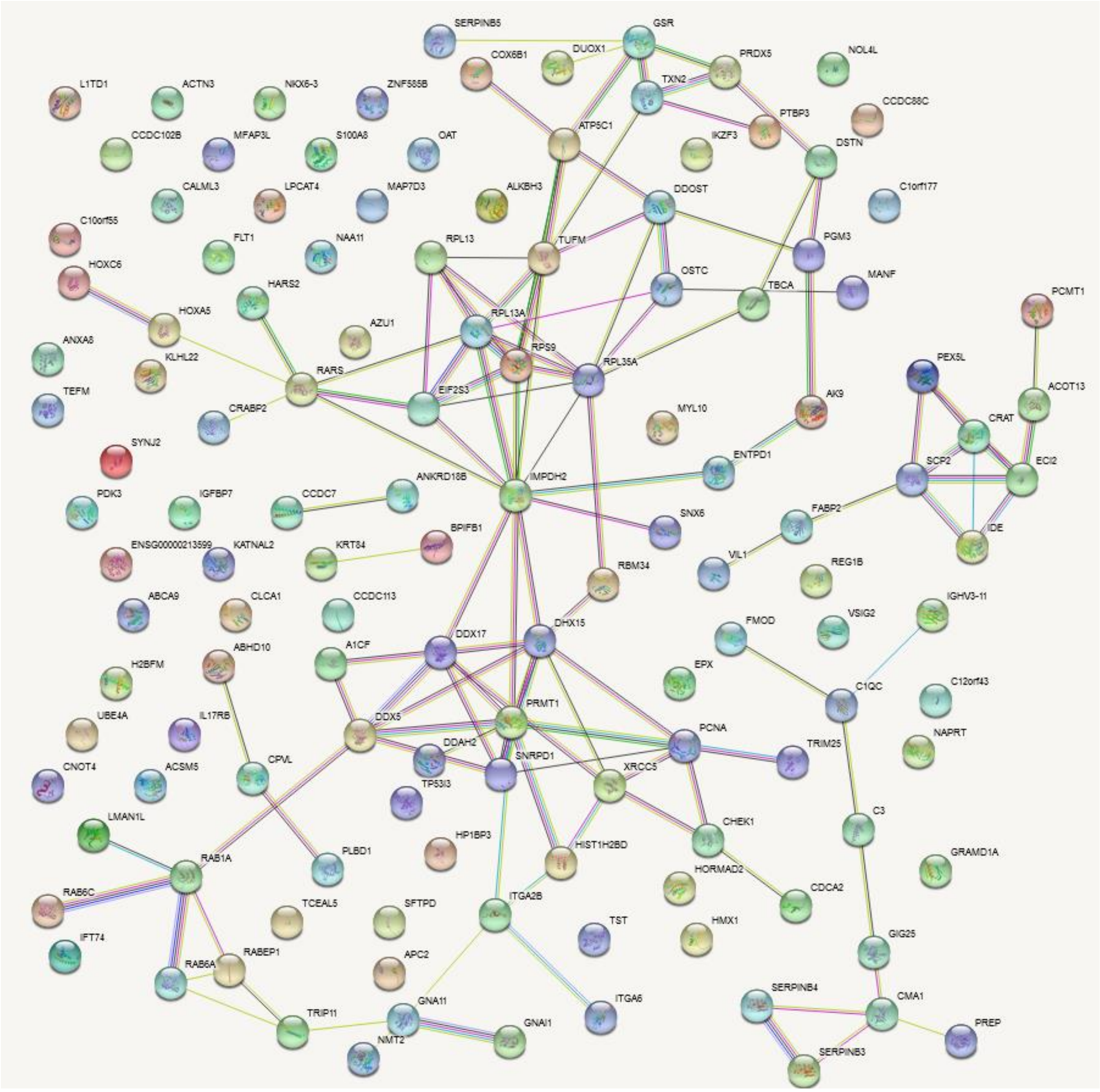

Figure S44: String analysis of shortlisted proteins upregulated in GC *vs.* NGM antrum.

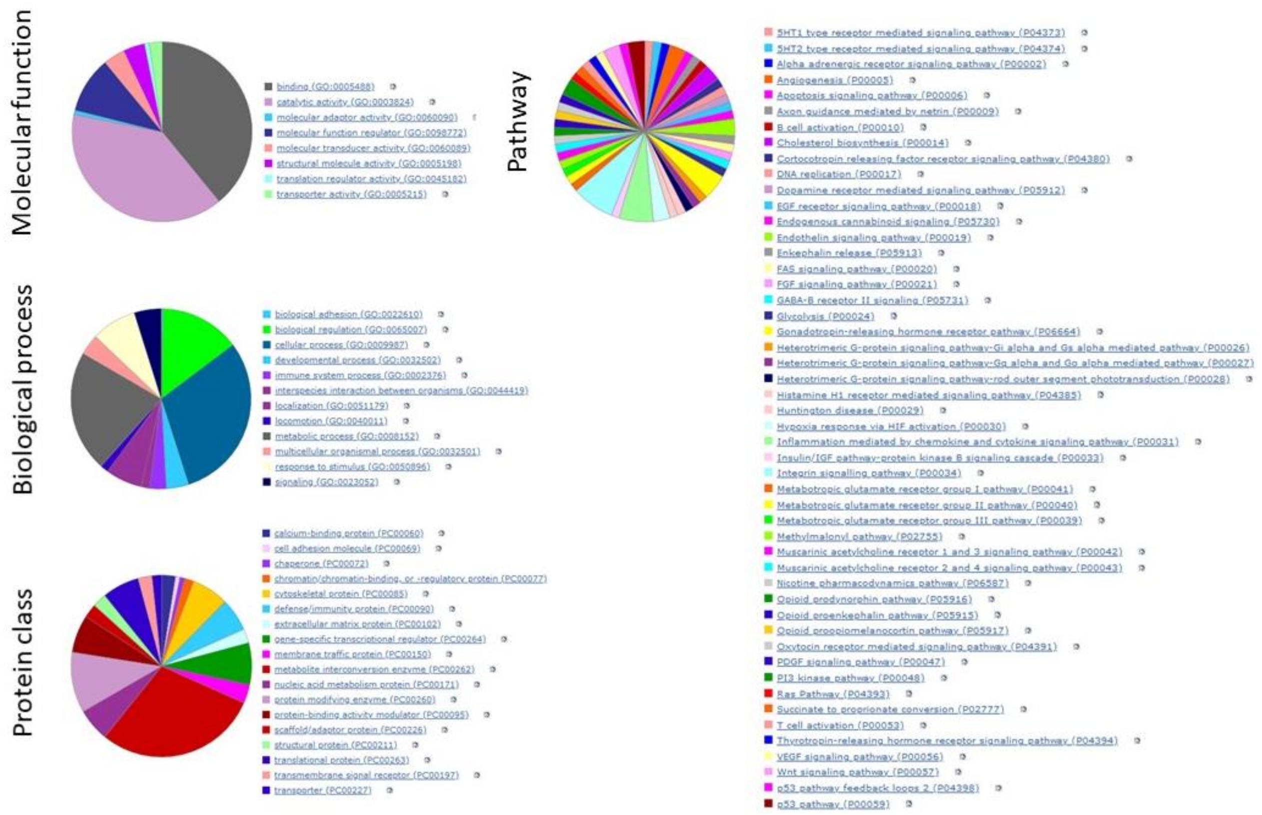

Figure S45: Functional classification of shortlisted proteins upregulated in GC *vs.* NGM antrum.

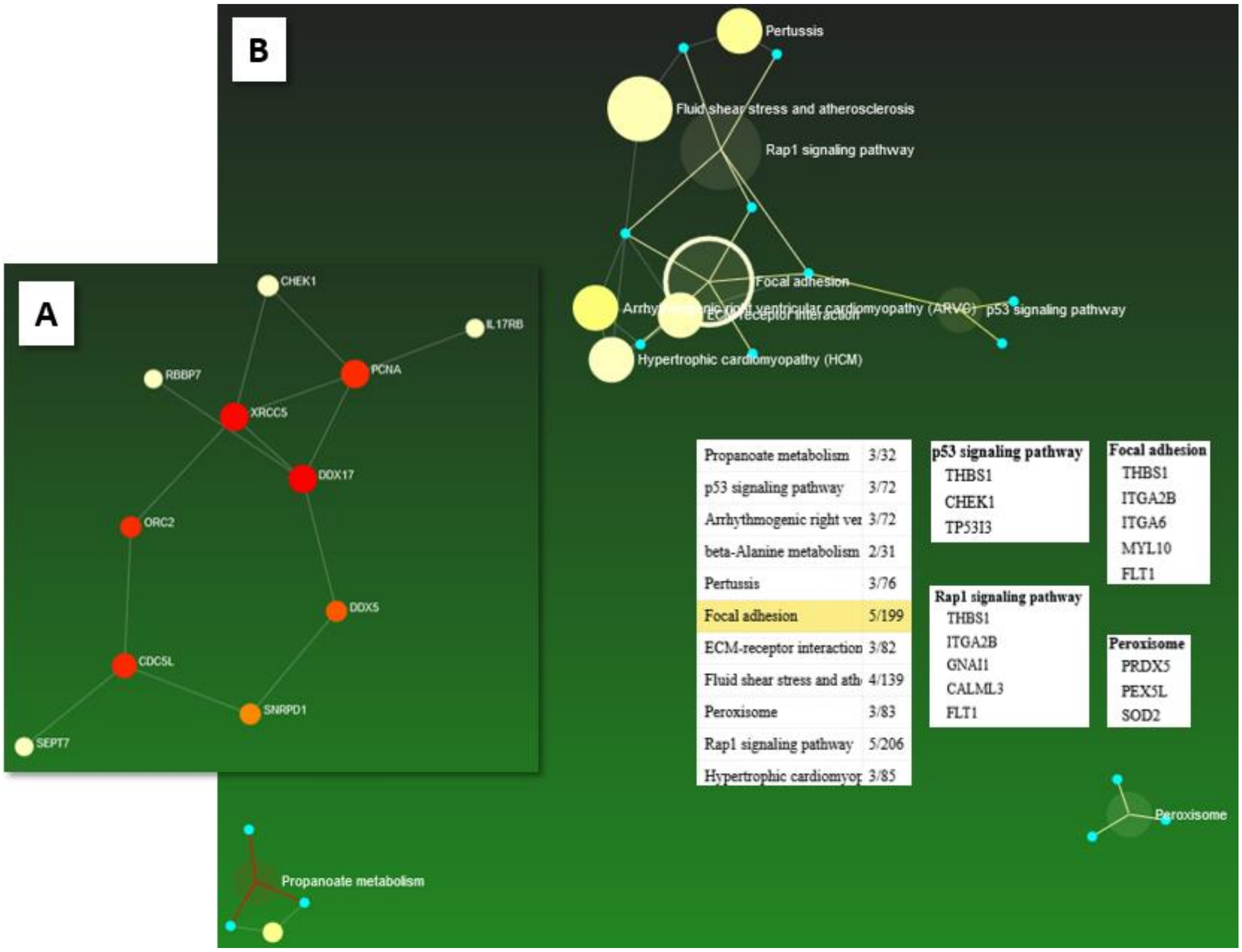

Figure S46: Network analysis (NetworkAnalyst) of shortlisted proteins upregulated in GC *vs.* NGM antrum HPpD. A) Zero-order network. B) Enrichment ORA network.

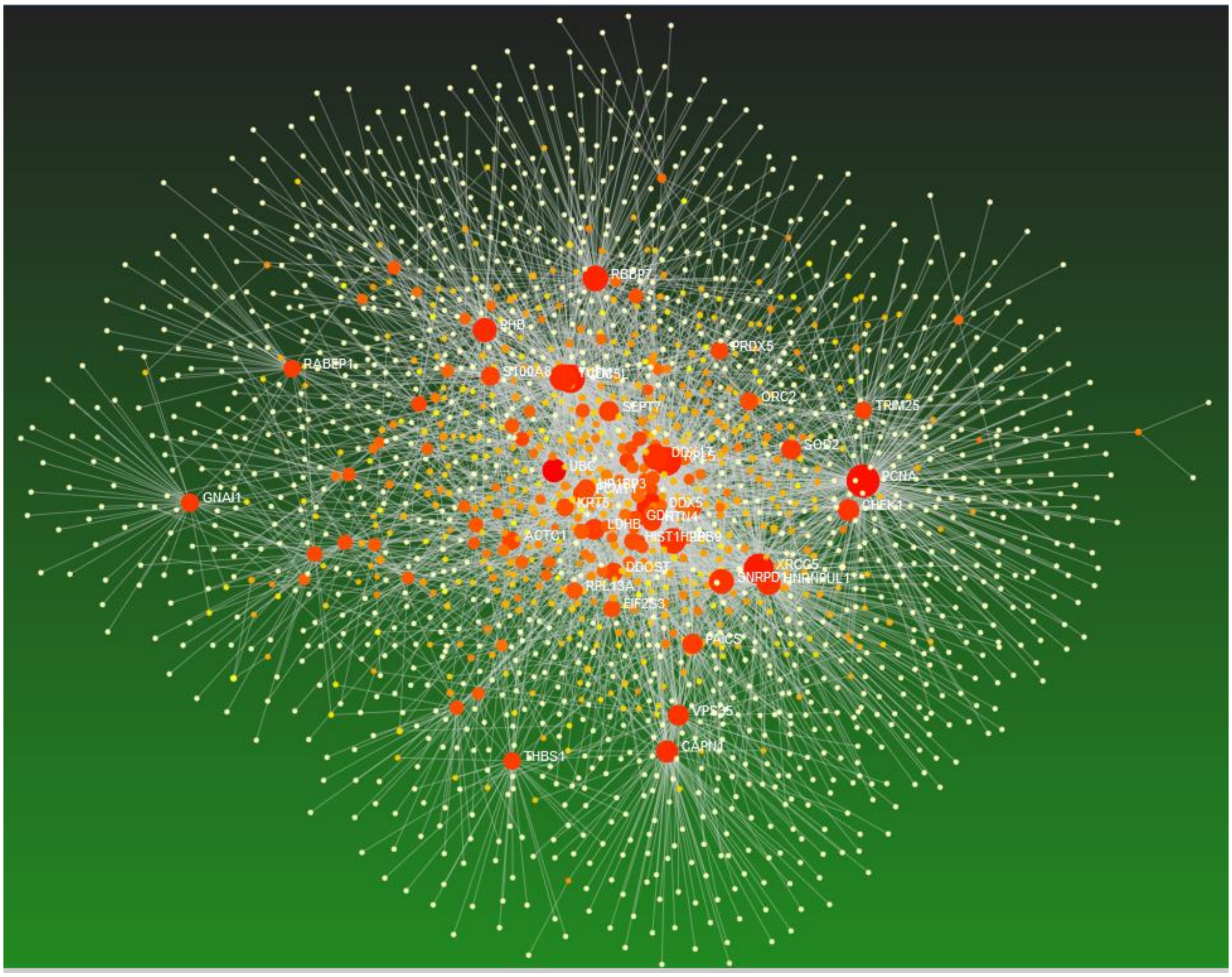

Figure S47: Network visualisation (NetworkAnalyst) of shortlisted proteins upregulated in GC *vs.* NGM antrum HPpD.

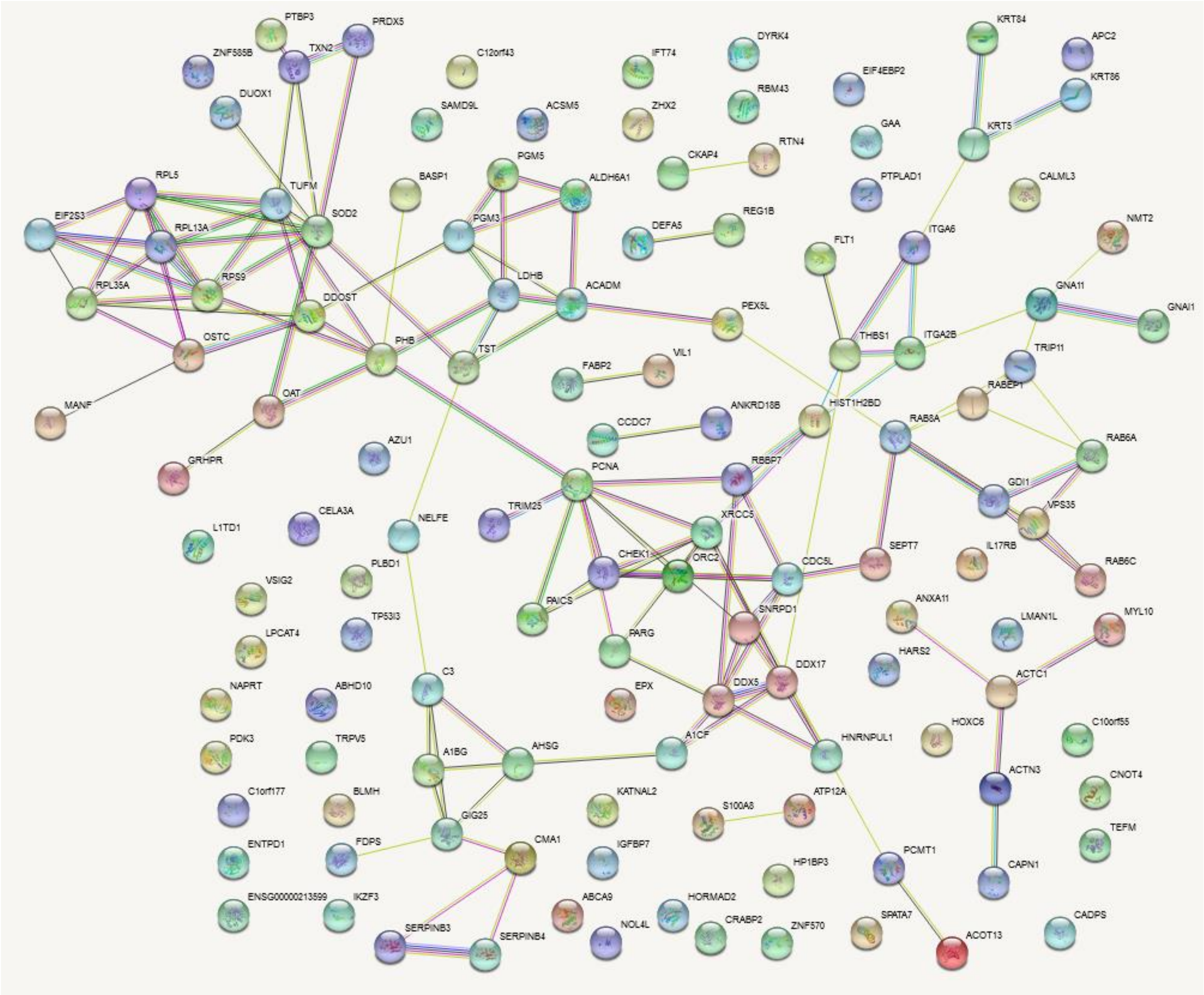

Figure S48: String analysis of shortlisted proteins upregulated in GC *vs*. NGM antrum HPpD.

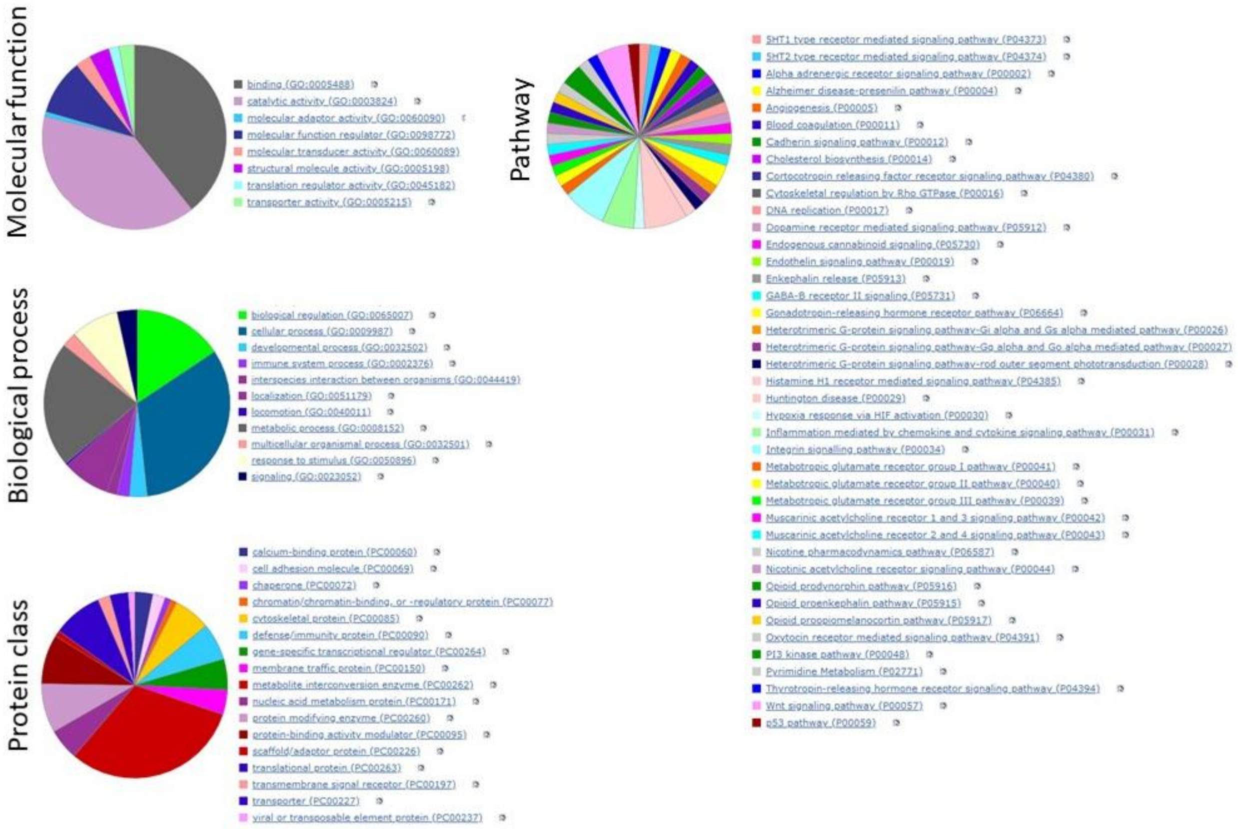

Figure S49: Functional classification of shortlisted proteins upregulated in GC *vs.* NGM antrum HPpD.

Table S3: Upregulated proteins in GC *vs.* NGM (antrum) independently of *H. pylori* infection (confidence cut-off 50, for complete list see Supplementary file "analysis"). *Proteins in bold:* Venn-extracted most abundant proteins (Supplementary Fig. S50), which were upregulated specifically in GC *vs.* all other gastrointestinal diseases when considering both all or only HPpD samples. This list comprises potential GC tissue marker proteins.

**Accession**	**Description**	**Name**
P01024	Complement C3	C3
Q08043	Alpha-actinin-3	ACTN3
P11678	Eosinophil peroxidase	EPX
Q92841	Probable ATP-dependent RNA helicase DDX17	DDX17
P13010	X-ray repair cross-complementing protein 5	XRCC5
P17844	Probable ATP-dependent RNA helicase DDX5	**DDX5**
P09327	Villin-1	**VIL1**
P30044	Peroxiredoxin-5_ mitochondrial	PRDX5
P01011	Alpha-1-antichymotrypsin	**SERPINA3**
P49411	Elongation factor Tu_ mitochondrial	**TUFM**

P17948	Vascular endothelial growth factor receptor 1	**FLT1**
P01834	Immunoglobulin kappa constant	**IGKC**
Q9NSB2	Keratin_ type II cuticular Hb4	KRT84
P39656	Dolichyl-diphosphooligosaccharide--protein glycosyltransferase 48 kDa subunit	DDOST
P41091	Eukaryotic translation initiation factor 2 subunit 3	**EIF2S3**
P58876	Histone H2B type 1-D	**H2BC5**
Q58FG1	Putative heat shock protein HSP 90-alpha A4	HSP90AA4P
Q9BUJ2	Heterogeneous nuclear ribonucleoprotein U-like protein 1	HNRNPUL1
P23229	Integrin alpha-6	**ITGA6**
P63096	Guanine nucleotide-binding protein G(i) subunit alpha-1	**GNAI1**
P20340	Ras-related protein Rab-6A	RAB6A
P05109	Protein S100-A8	S100A8
Q16762	Thiosulfate sulfurtransferase	**TST**
Q6XQN6	Nicotinate phosphoribosyltransferase	NAPRT
O43790	Keratin_ type II cuticular Hb6	KRT86
P46781	40S ribosomal protein S9	**RPS9**
P49961	Ectonucleoside triphosphate diphosphohydrolase 1	**ENTPD1**
Q9NUJ1	Palmitoyl-protein thioesterase ABHD10_ mitochondrial	**ABHD10**
P49590	Histidine-tRNA ligase, mitochondrial	HARS2
O95758	Polypyrimidine tract-binding protein 3	**PTBP3**
P55145	Mesencephalic astrocyte-derived neurotrophic factor	**MANF**
P0DMM9	Sulfotransferase 1A3	**SULT1A3**
P29508	Serpin B3	**SERPINB3**
Q8IYT4	Katanin p60 ATPase-containing subunit A-like 2	**KATNAL2**
Q53FA7	Quinone oxidoreductase PIG3	TP53I3
P29992	Guanine nucleotide-binding protein subunit alpha-11	**GNA11**
P12004	Proliferating cell nuclear antigen	**PCNA**
P30711	Glutathione S-transferase theta-1	**GSTT1**
P40429	60S ribosomal protein L13a	**RPL13A**
Q96LB3	Intraflagellar transport protein 74 homolog	**IFT74**
Q5T7N2	LINE-1 type transposase domain-containing protein 1	L1TD1
O95394	Phosphoacetylglucosamine mutase	PGM3
P48594	Serpin B4	**SERPINB4**
Q6P4A8	Phospholipase B-like 1	**PLBD1**
Q96IQ7	V-set and immunoglobulin domain-containing protein 2	**VSIG2**
P04181	Ornithine aminotransferase_ mitochondrial	**OAT**
P22061	Protein-L-isoaspartate(D-aspartate) O-methyltransferase	**PCMT1**
P27482	Calmodulin-like protein 3	**CALML3**
Q6NUN0	Acyl-coenzyme A synthetase ACSM5_ mitochondrial	ACSM5
Q9NPJ3	Acyl-coenzyme A thioesterase 13	**ACOT13**

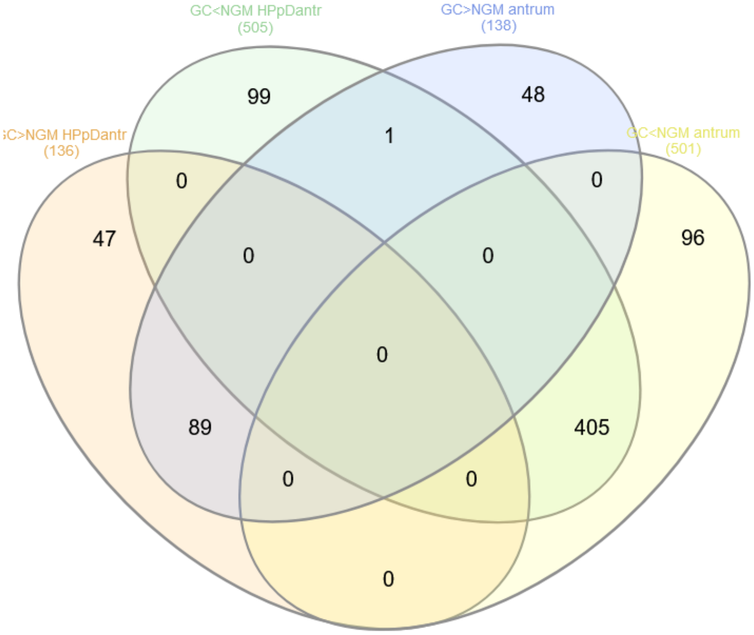

Figure 50: Venn diagram for the comparison of antrum NGM vs. GC – all samples or HPpD. See protein lists in Supplementary file “analysis”.

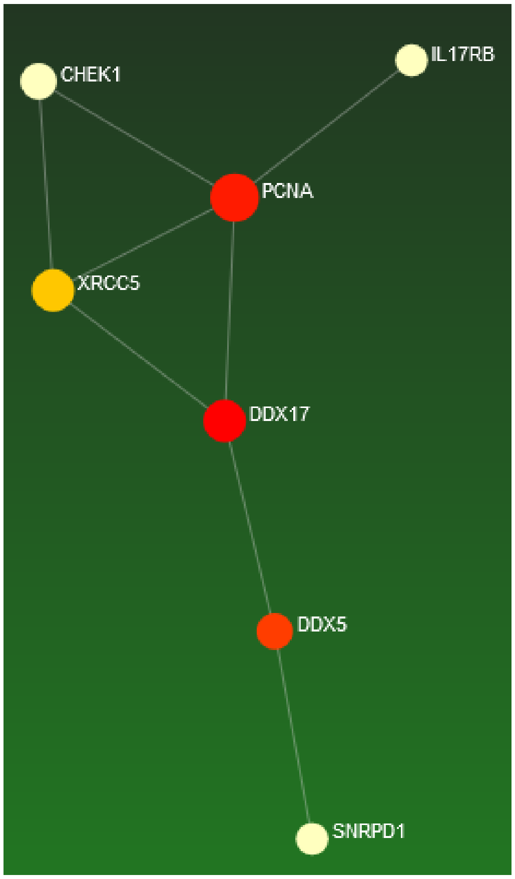

Figure S51: Zero order network of shortlisted proteins upregulated in GC vs. NGM antrum and common for *H. pylori* independent and dependent cancers.

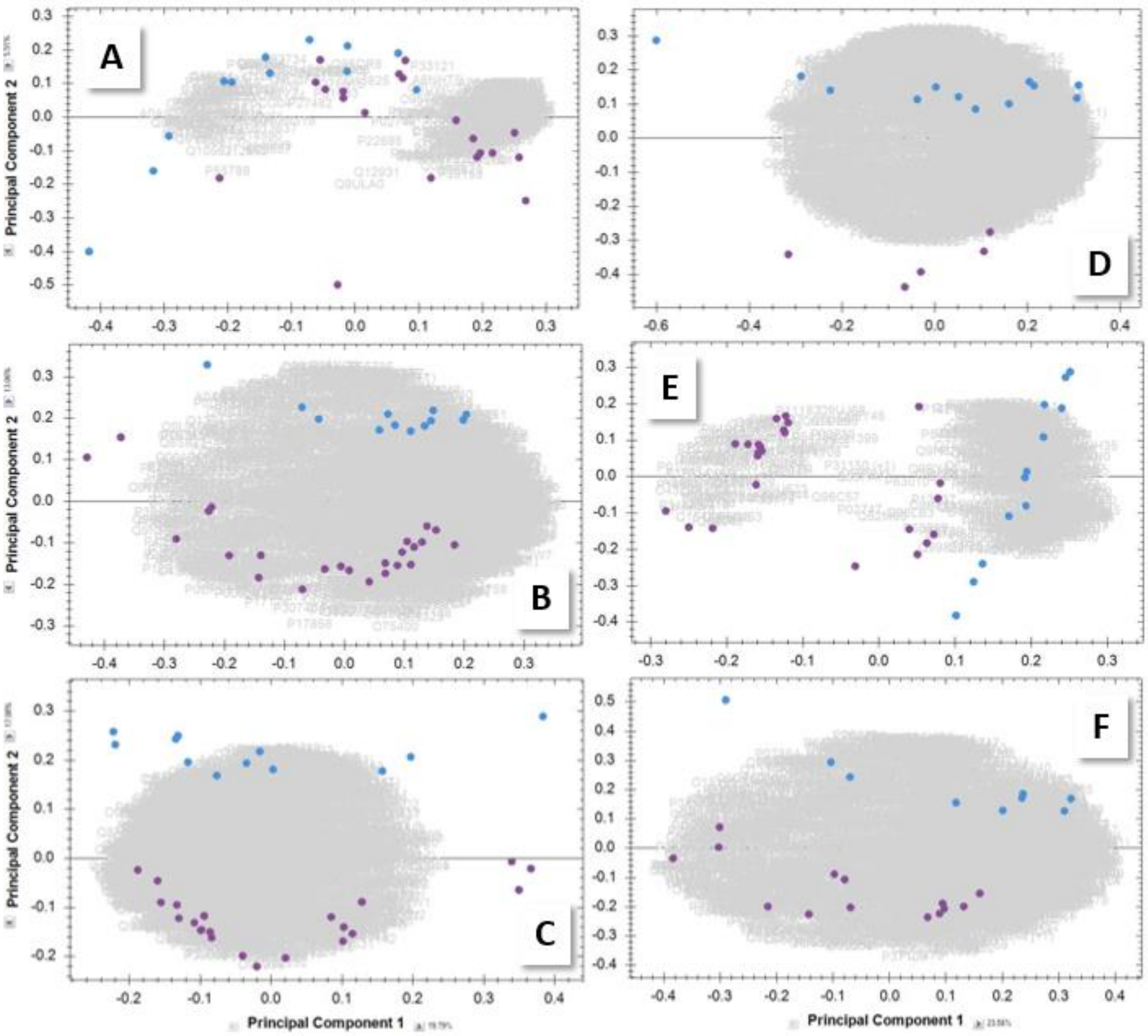

Figure S52: PCA of shortlisted proteins for comparison to NGM of A) MiG (purple), E) U (purple). PCA of entire data (2 matched peptides) of B) MoG (purple), C) MaG (purple), D) PanG (purple), F) Combined MoG / MaG HPpD (purple).

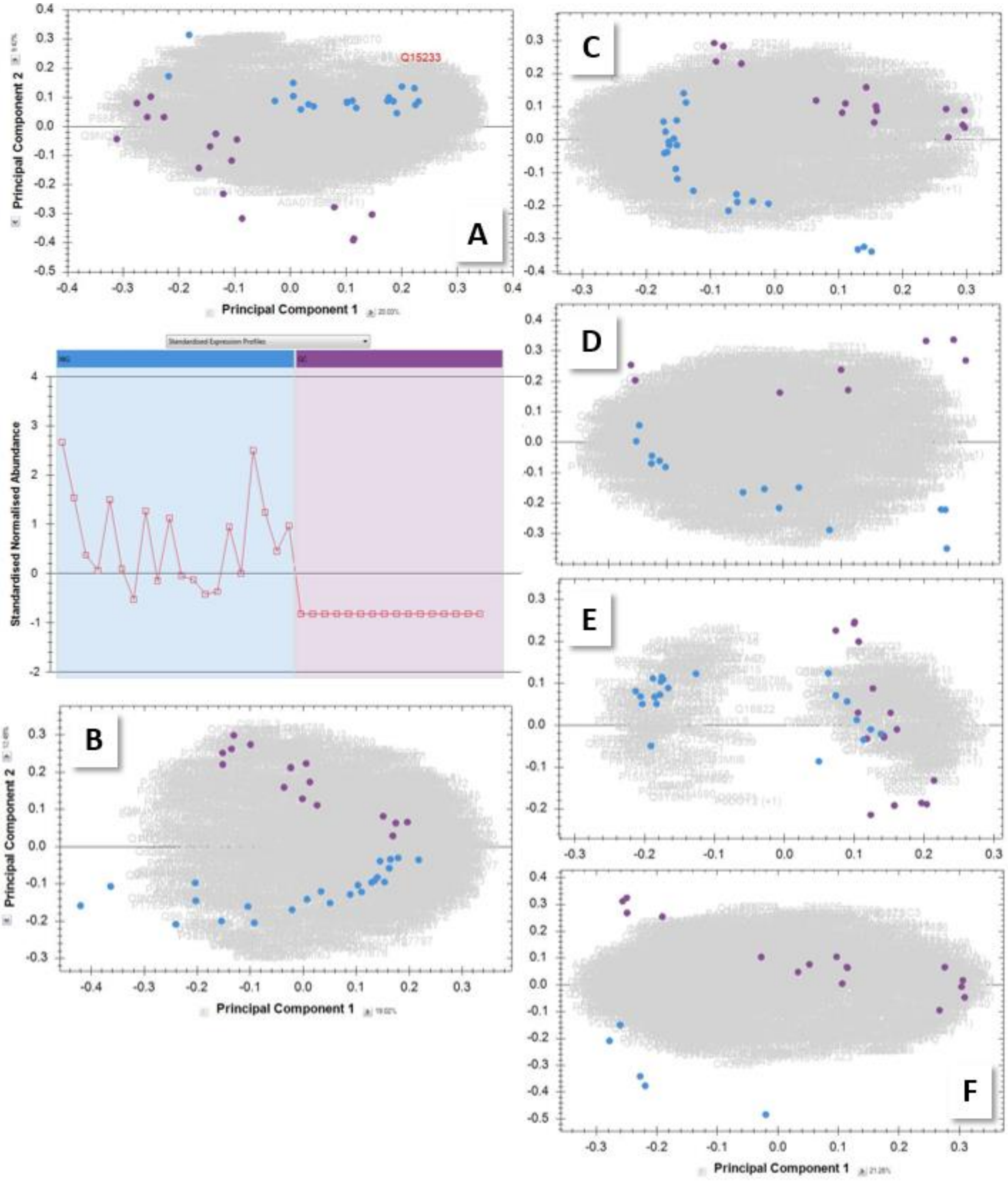

Figure S53: PCA of entire data (2 matched peptides) for comparison to GC of A) MiG (blue, with expression profile of Q15233 - non-POU domain-containing octamer-binding protein), B) MoG (blue), C) MaG (blue), D) Combined MoG / MaG (blue), F) PanG (blue). PCA of shortlisted proteins of E) U (blue).

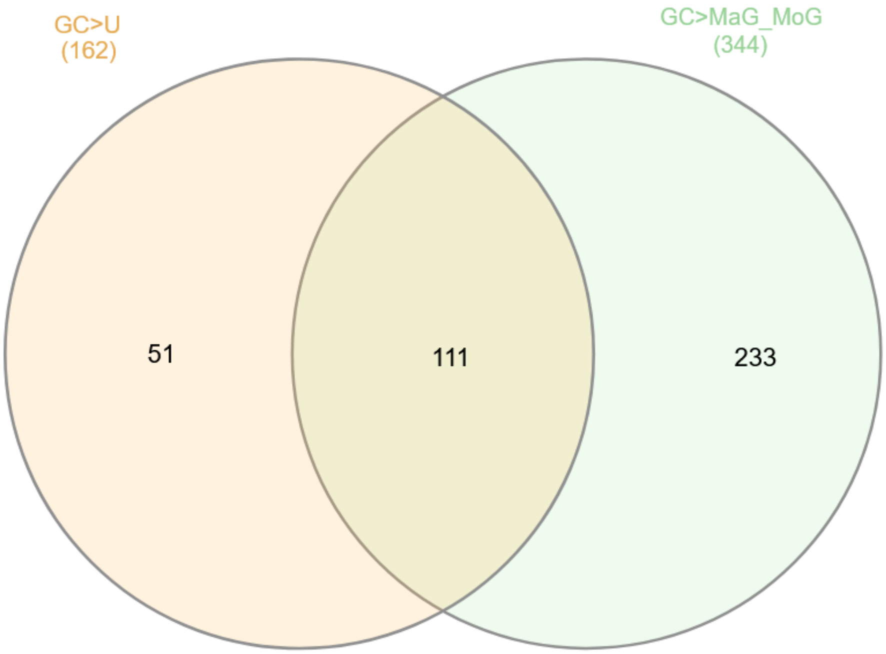

Figure S54: Venn diagram comparing the shortlisted upregulated proteins in HPpD GC compared to U and combined MoG/MaG. The respective proteins are shown in Supplementary file "analysis".

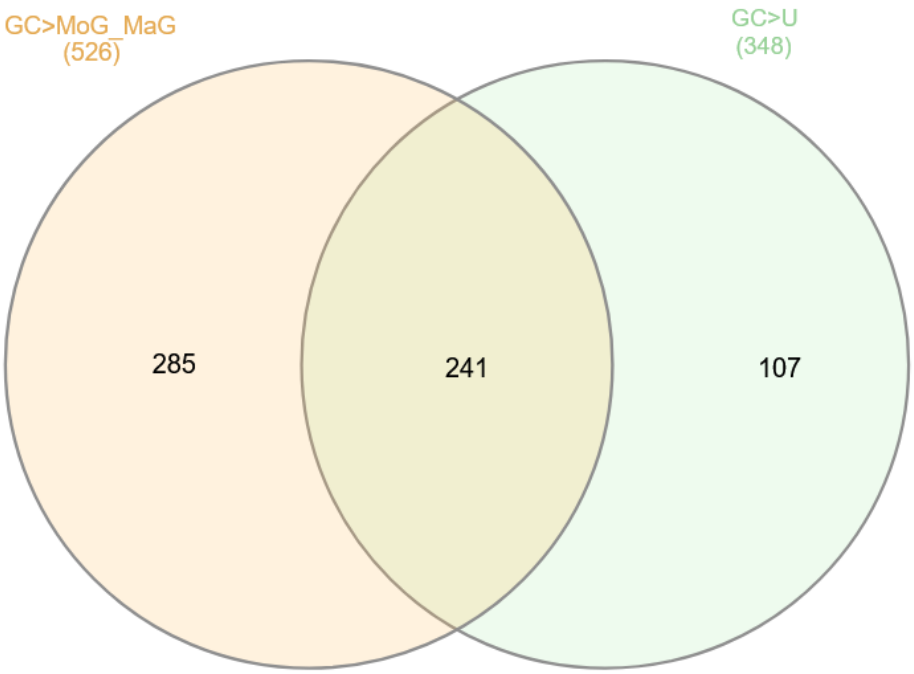

Figure S55: Venn diagram comparing the shortlisted upregulated proteins in GC compared to U and combined MoG/MaG. The respective proteins are shown in Supplementary file "analysis".

Serum analysis

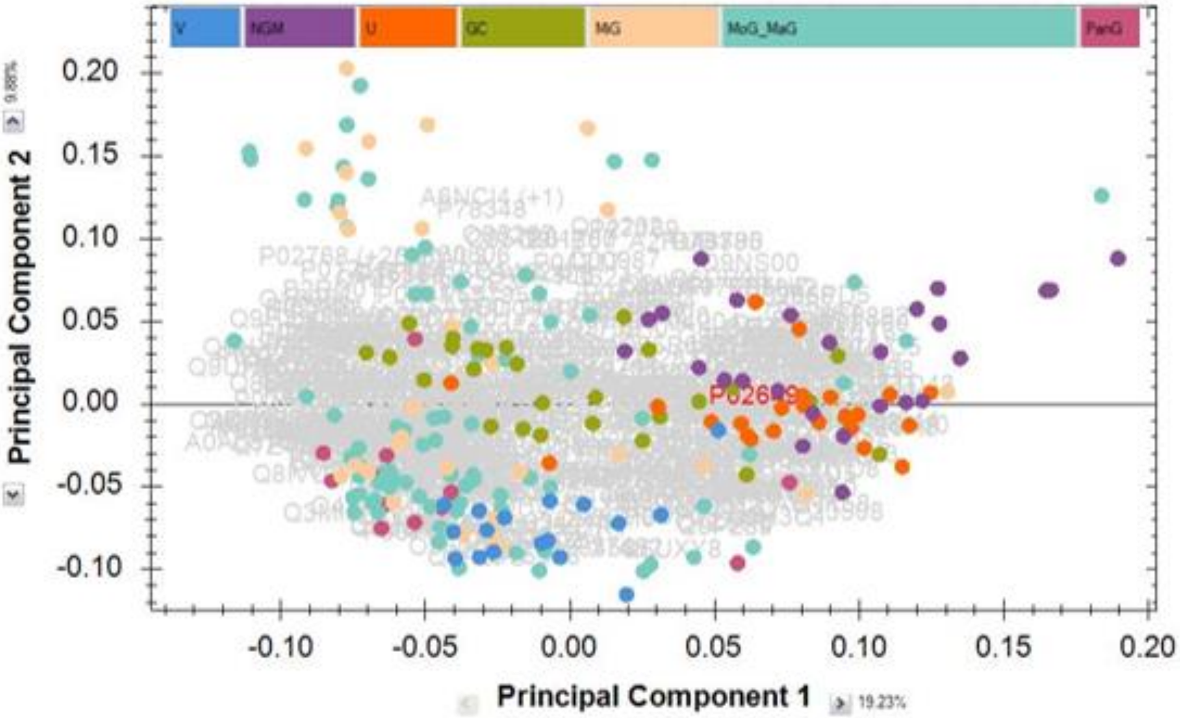

Figure 56: PCA of serum samples based on the clinical assignment. MoG and MaG were combined for clarity.

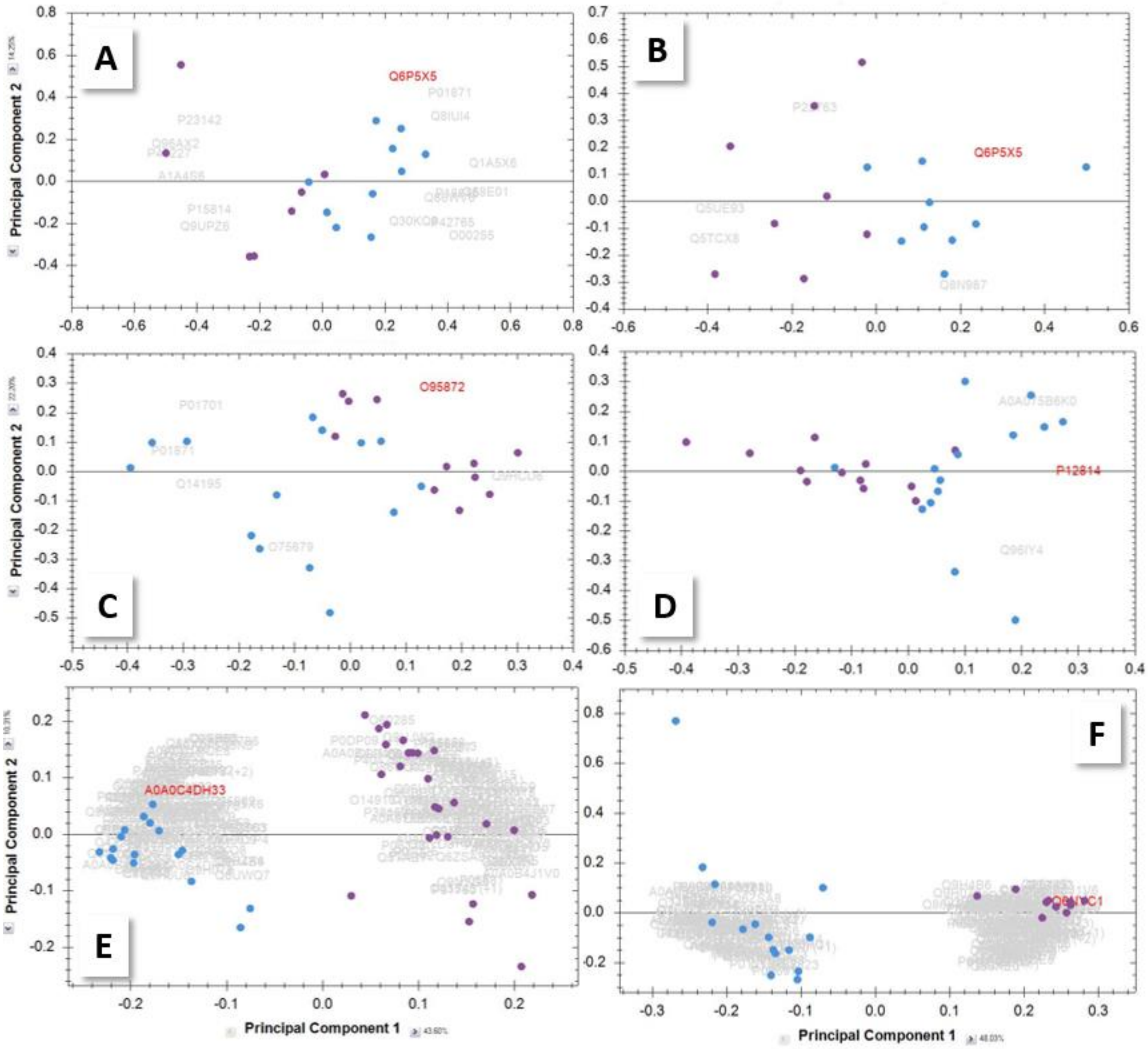

Figure S57: PCA of shortlisted data for comparison of A) V HPp/m (HPp blue), B) V gender (female blue), C) NGM HPp/m (HPp blue), D) NGM gender (female blue), E) NGM *vs.* V (V blue), F) NGM *vs.* V HPp (NGM blue).

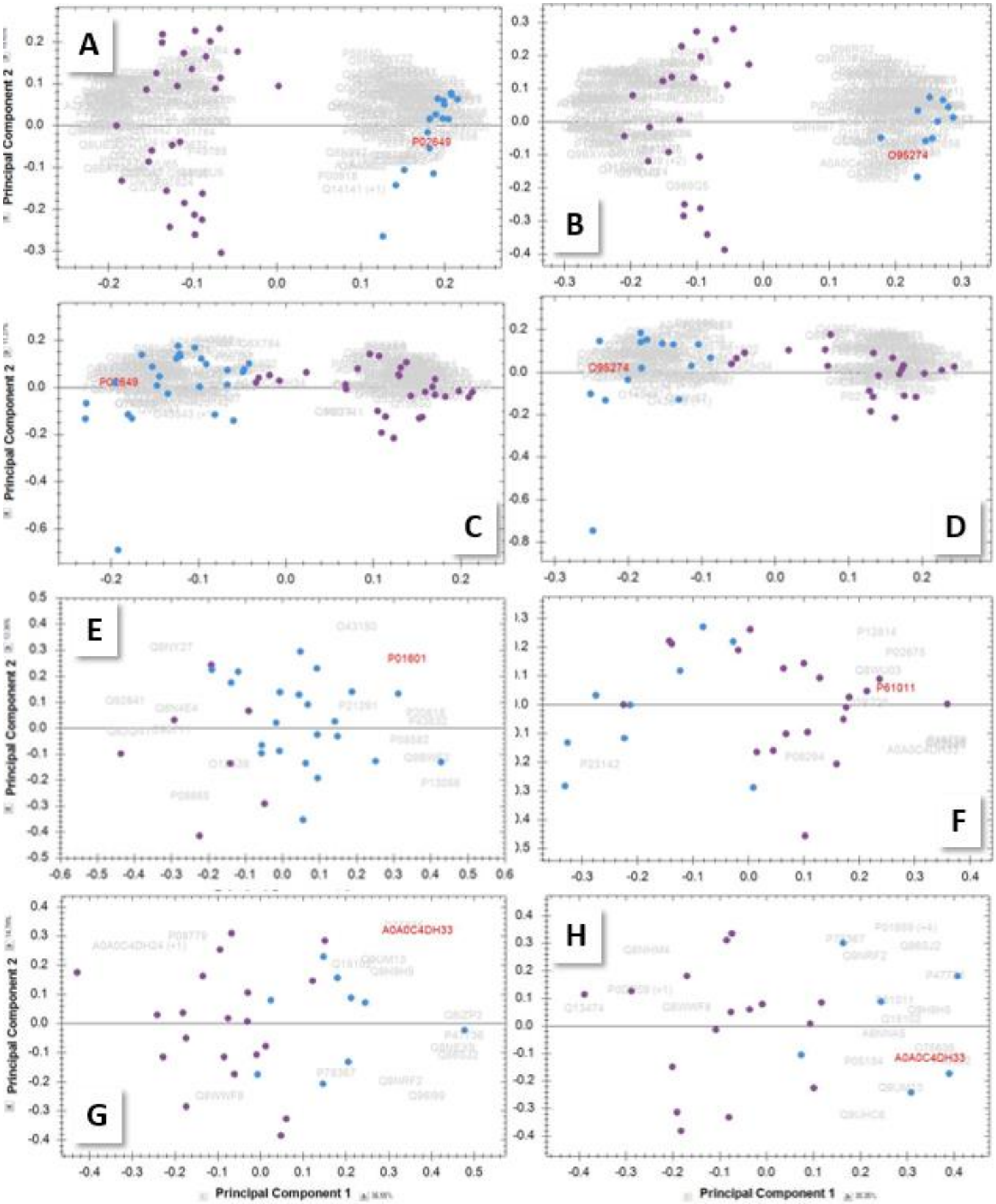

Figure S58: PCA of shortlisted data for comparison of A) GC *vs.* V (V blue), B) GC *vs.* V HPp/m (V blue), C) GC *vs.* NGM (NGM blue), D) GC *vs.* NGM HPp (NGM blue), E) GC HPp/m (HPp blue), F) GC advanced *vs.* 1st stage (1st stage blue), G) GC gender (female blue), H) GC gender HPp/m (female blue).

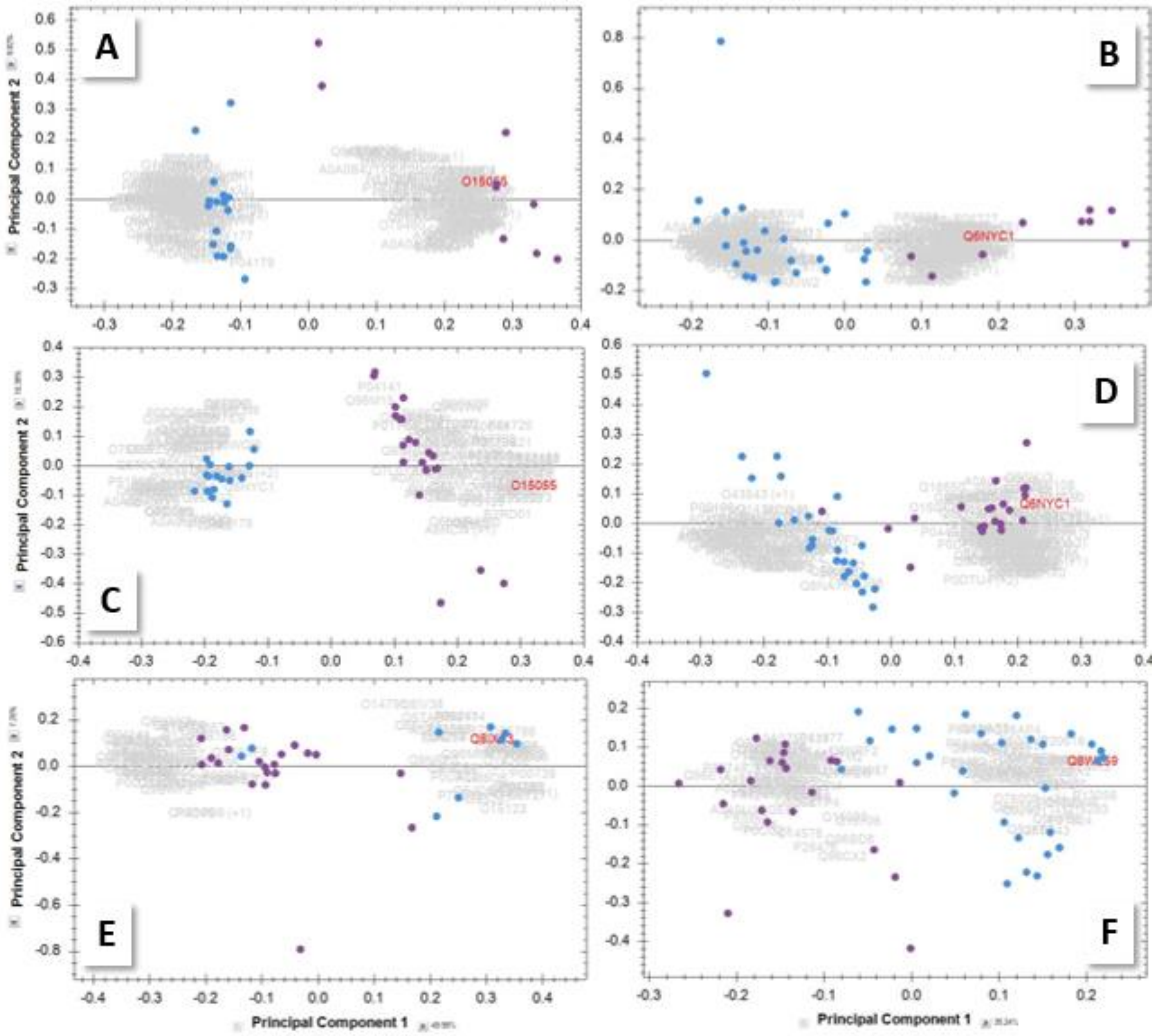

Figure S59: PCA of shortlisted data for comparison of A) MiGn *vs.* V (V blue), B) MiGn *vs.* NGM (NGM blue), C) MiGa *vs.* V (V blue), D) MiGa *vs.* NGM (NGM blue), E) MiGa/n (MiGn blue), F) MiGa *vs.* GC (GC blue).

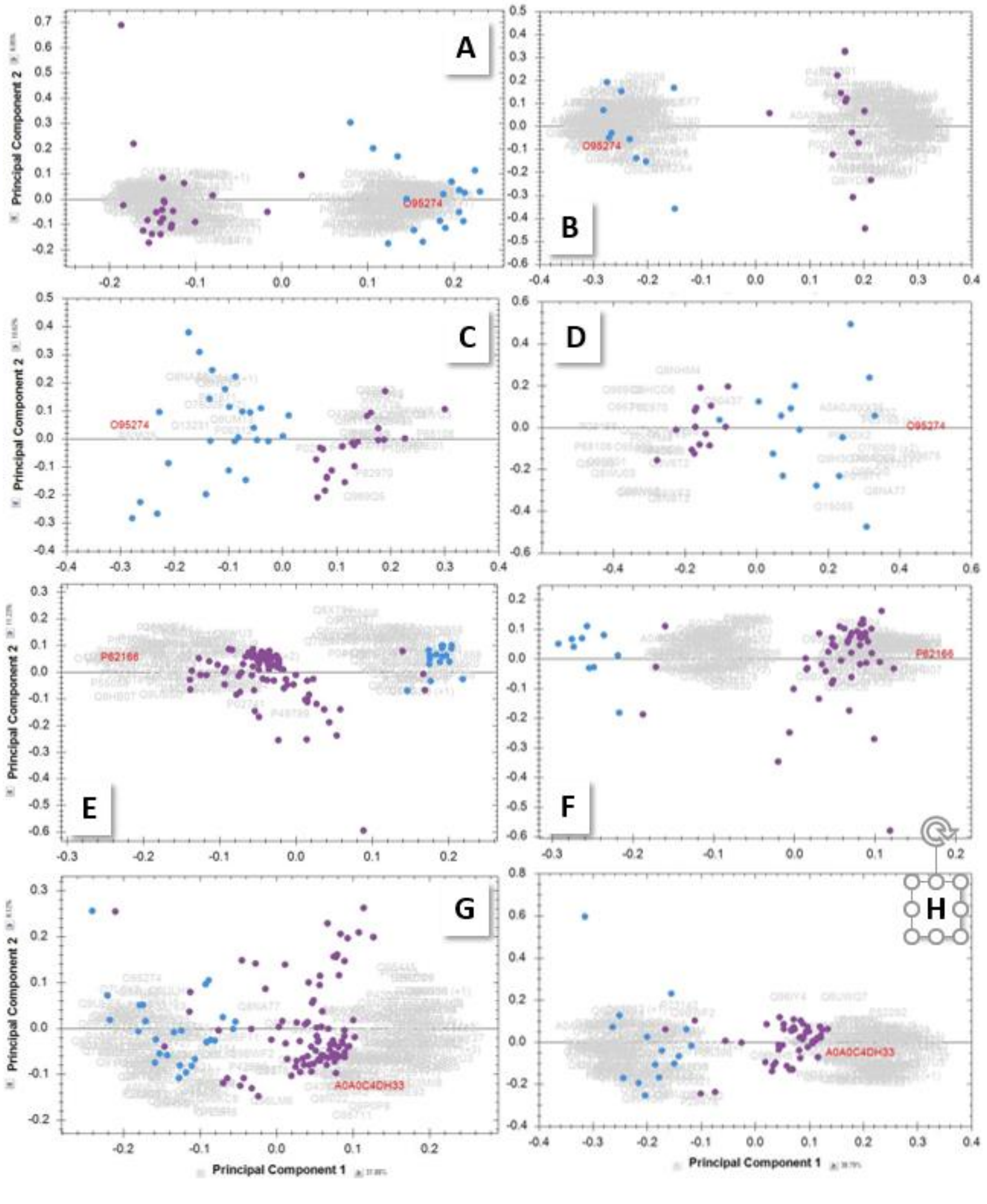

Figure S60: PCA of shortlisted data for comparison of A) V *vs*. U (V blue), B) V *vs*. U HPp (V blue), C) NGM *vs*. U (NGM blue), D) NGM *vs.* U HPp (NGM blue), E) V *vs.* MoG/MaG/PanG (V blue), F) V *vs*. MoG/MaG/PanG HPp (V blue), G) NGM *vs*. MoG/MaG/PanG (NGM blue), H) NGM *vs*. MoG/MaG/PanG HPp (NGM blue).

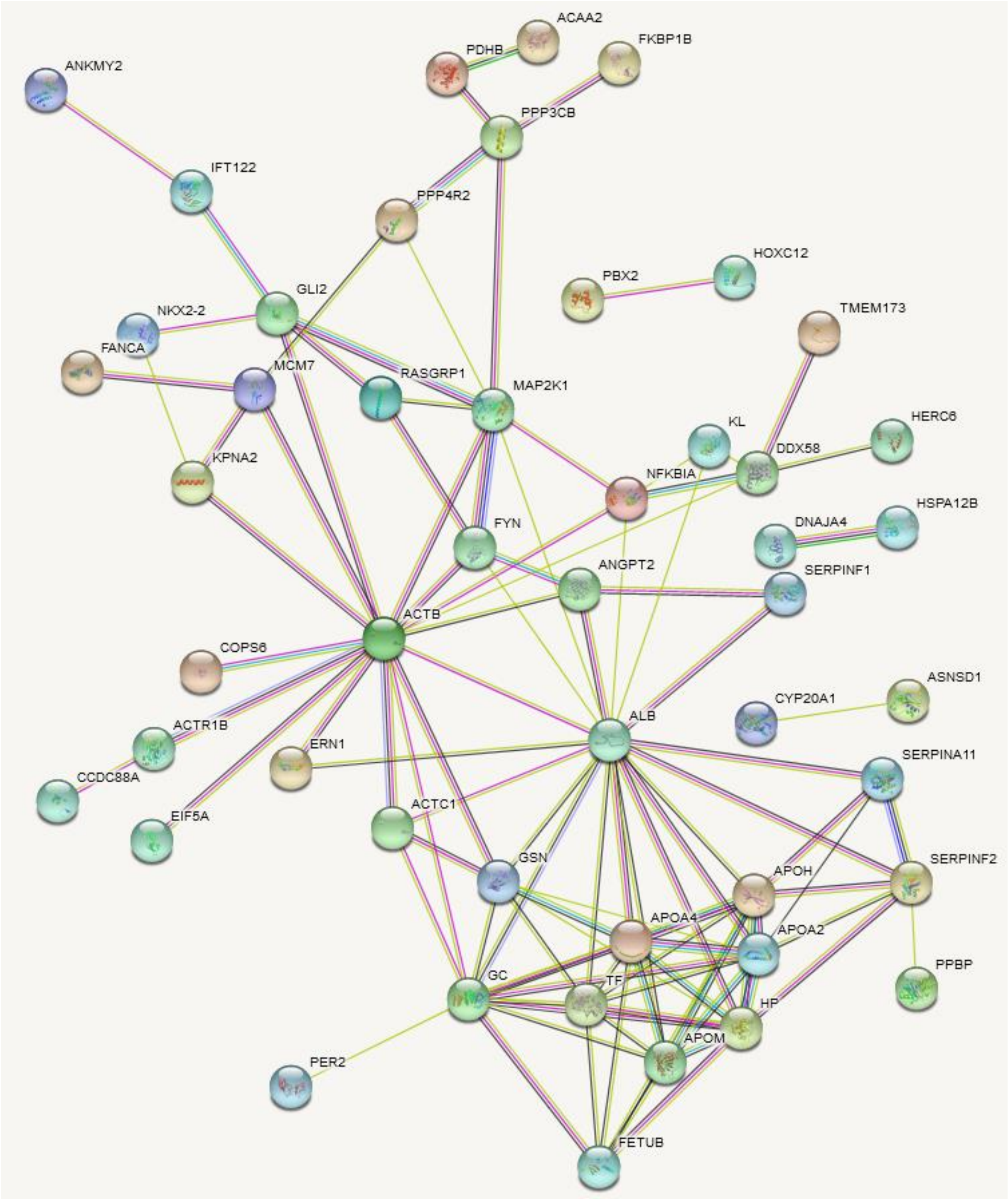

Figure S61: String network of shortlisted proteins upregulated in MiGn *vs*. NGM.

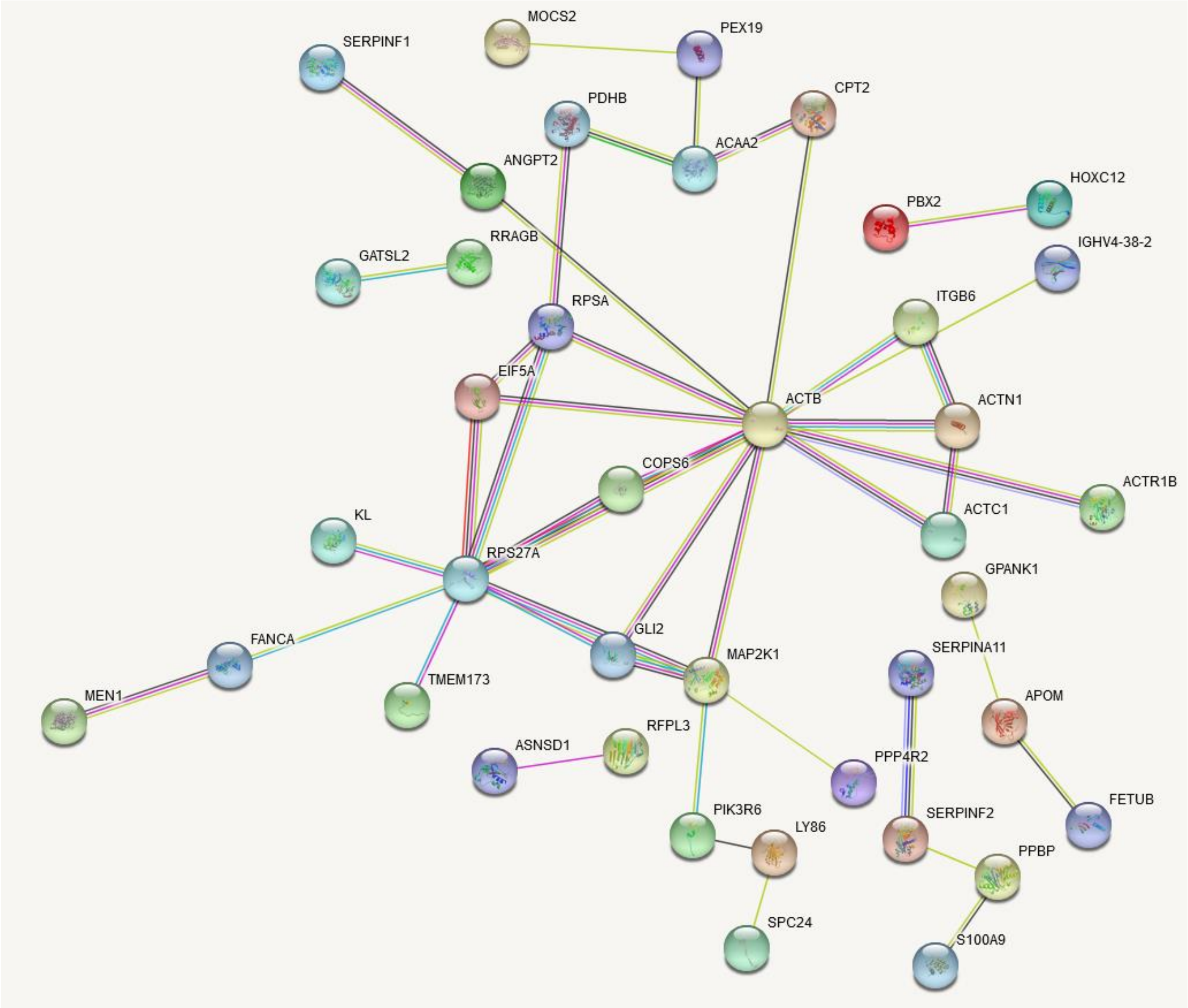

Figure S62: String network of shortlisted proteins upregulated in MiGa *vs*. NGM.

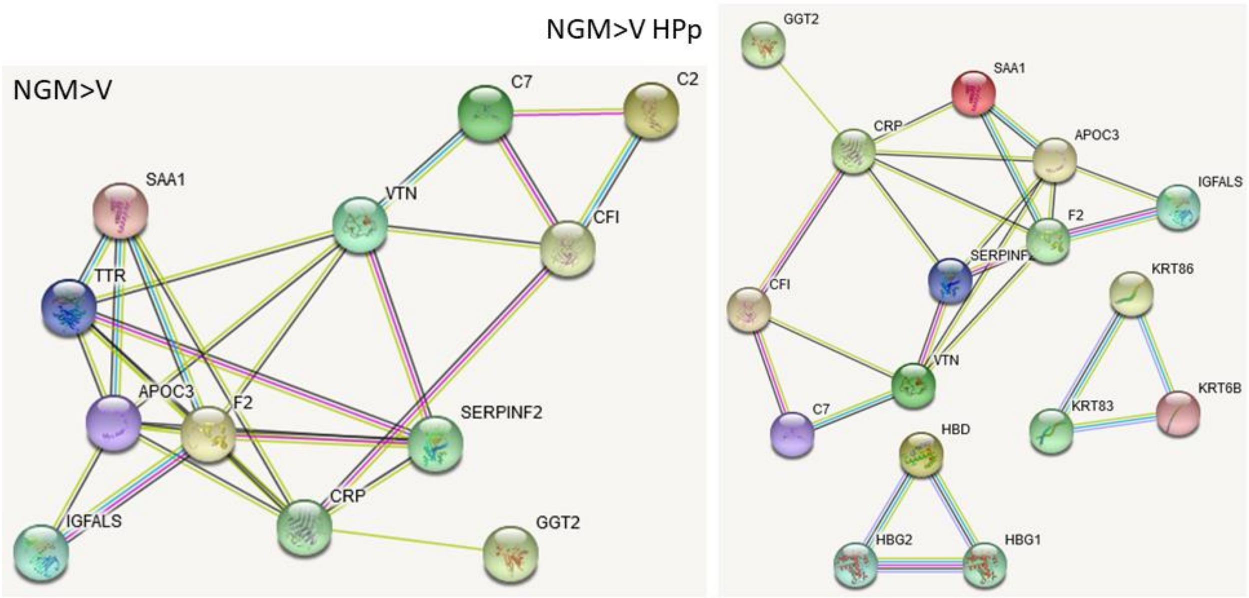

Figure S63: String networks of upregulated proteins in NGM and NGM HPp *vs.* V.

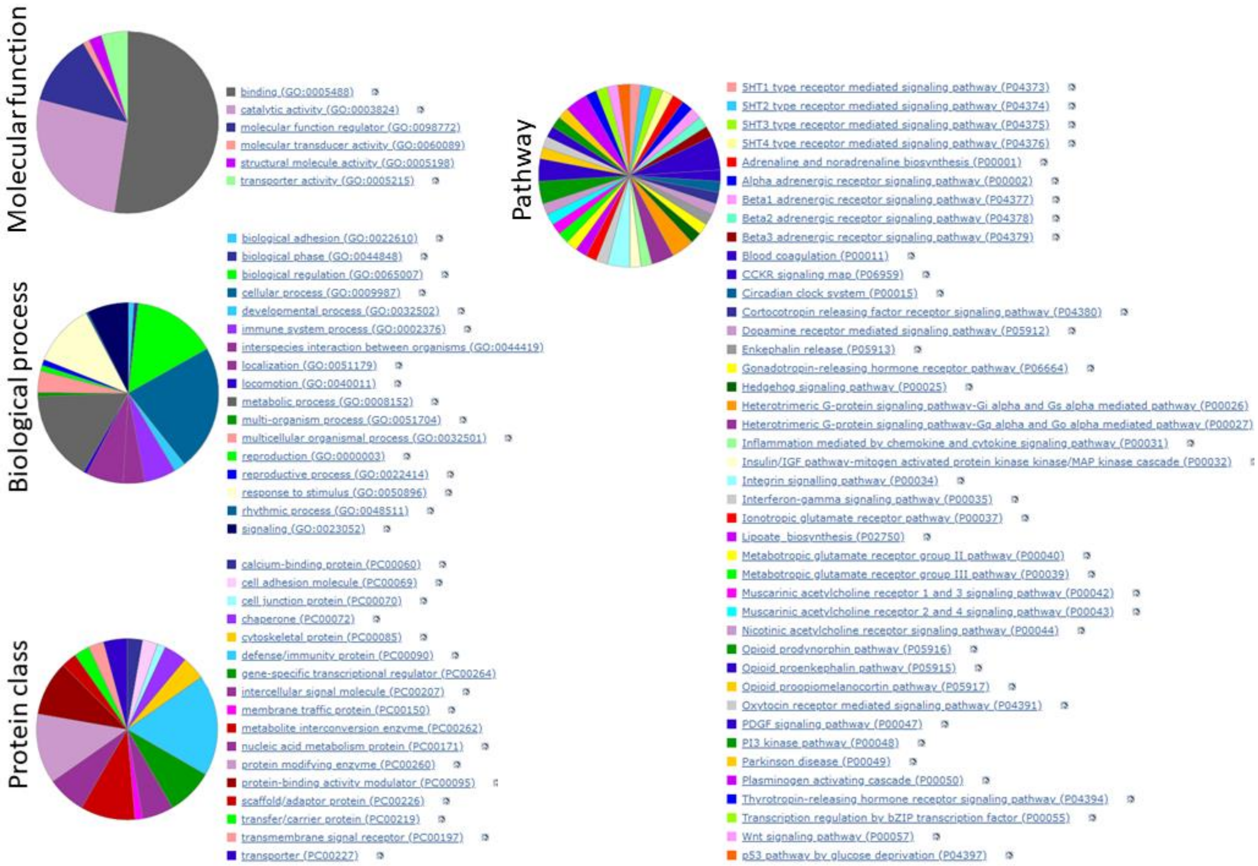

Figure S64: Functional classification of upregulated proteins in NGM *vs.* V.

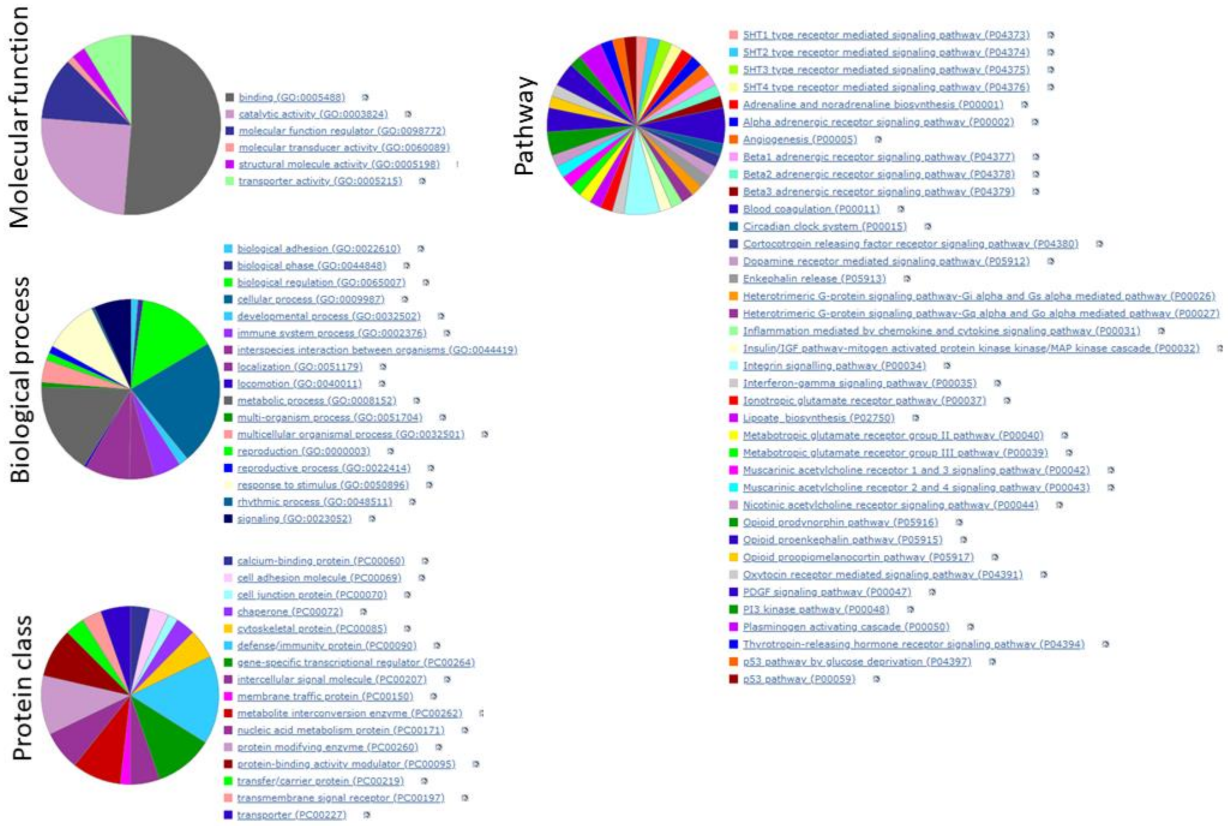

Figure S65: Functional classification of upregulated proteins in NGM *vs*. V HPp.

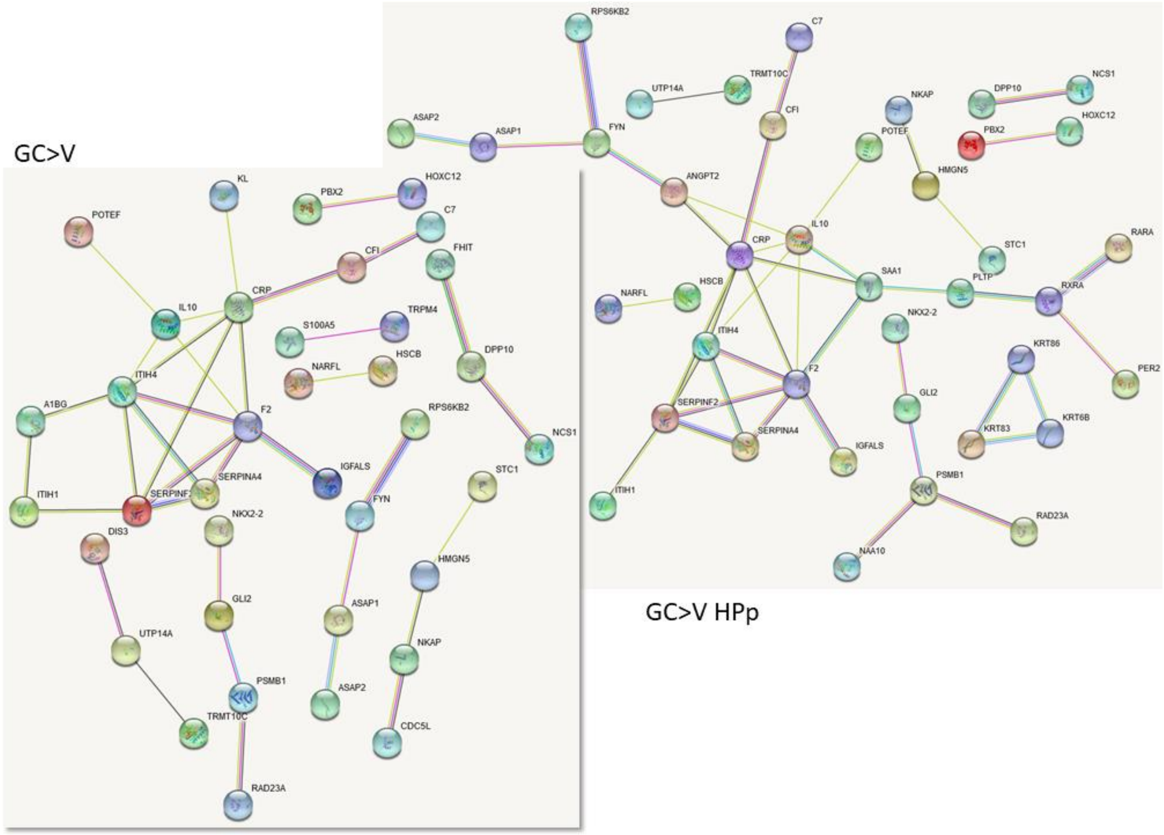

Figure S66: String network of shortlisted proteins upregulated in GC and GC HPp *vs*. V.

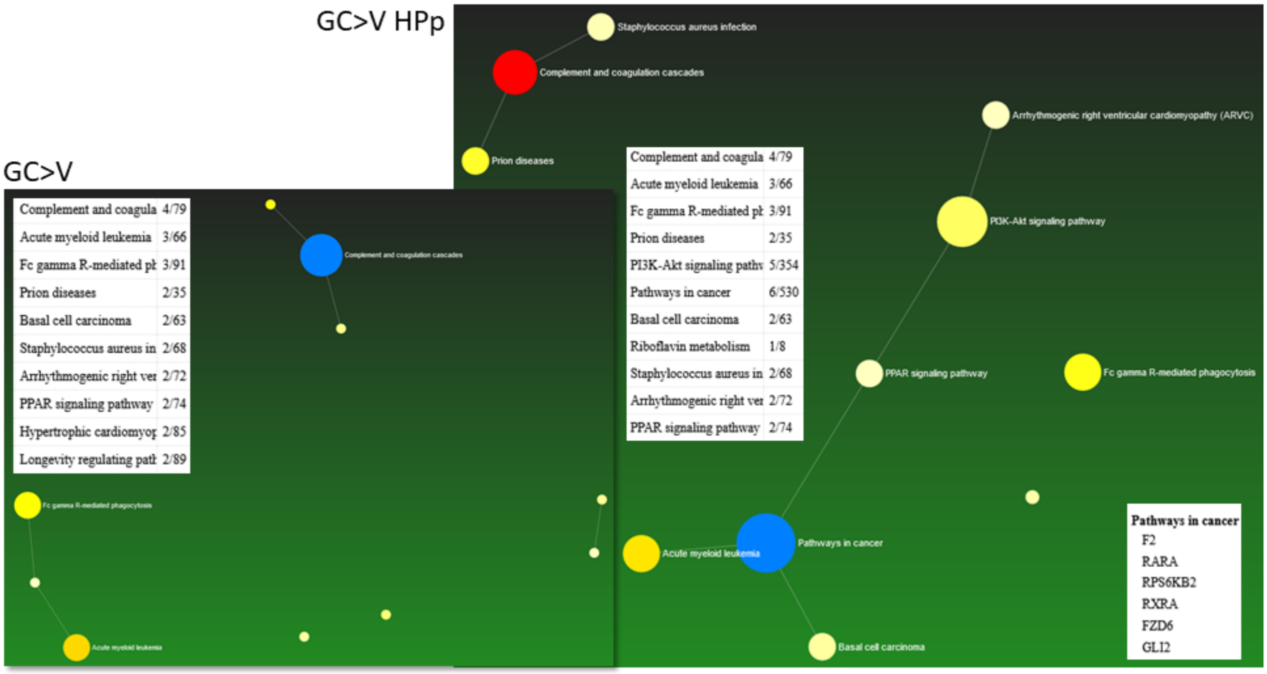

Figure S67: ORA network of upregulated shortlisted proteins in GC and GC HPp *vs*. V.

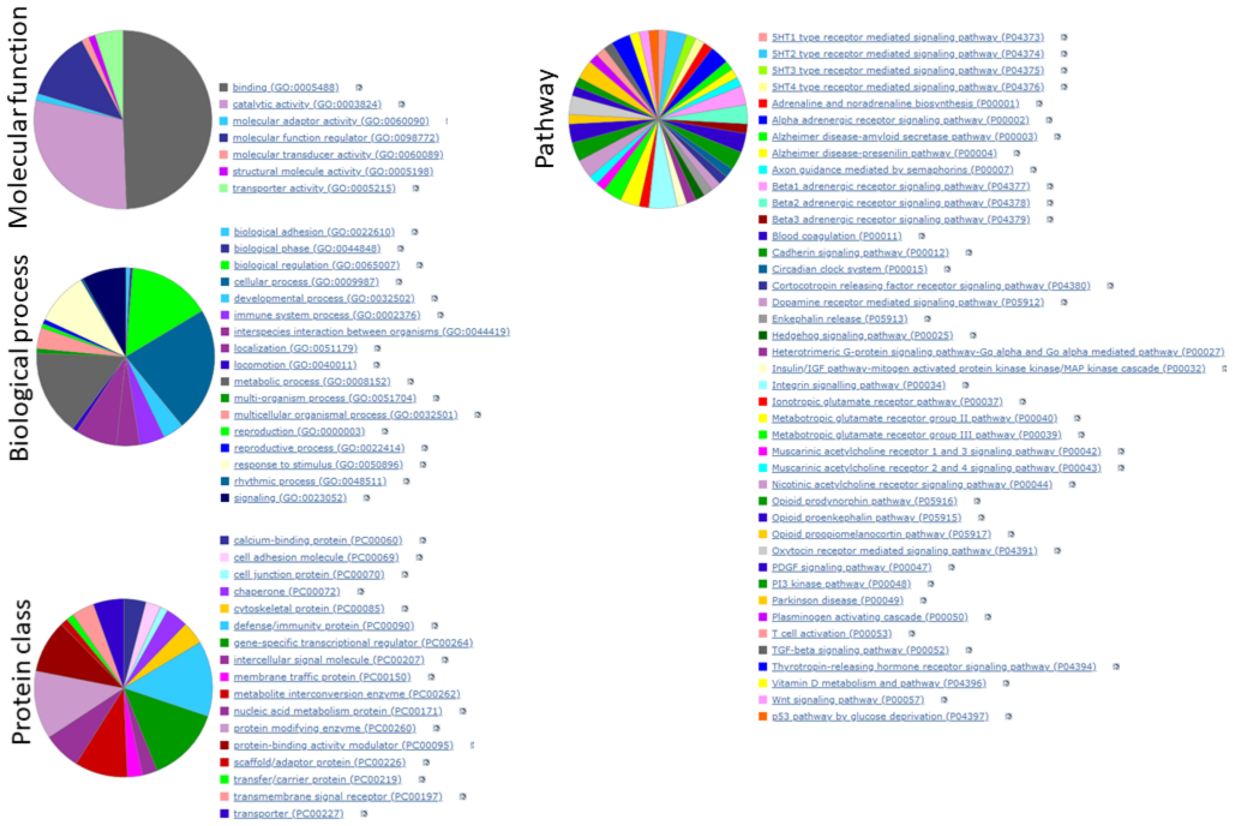

Figure S68: Functional classification of upregulated proteins in GC *vs.* V.

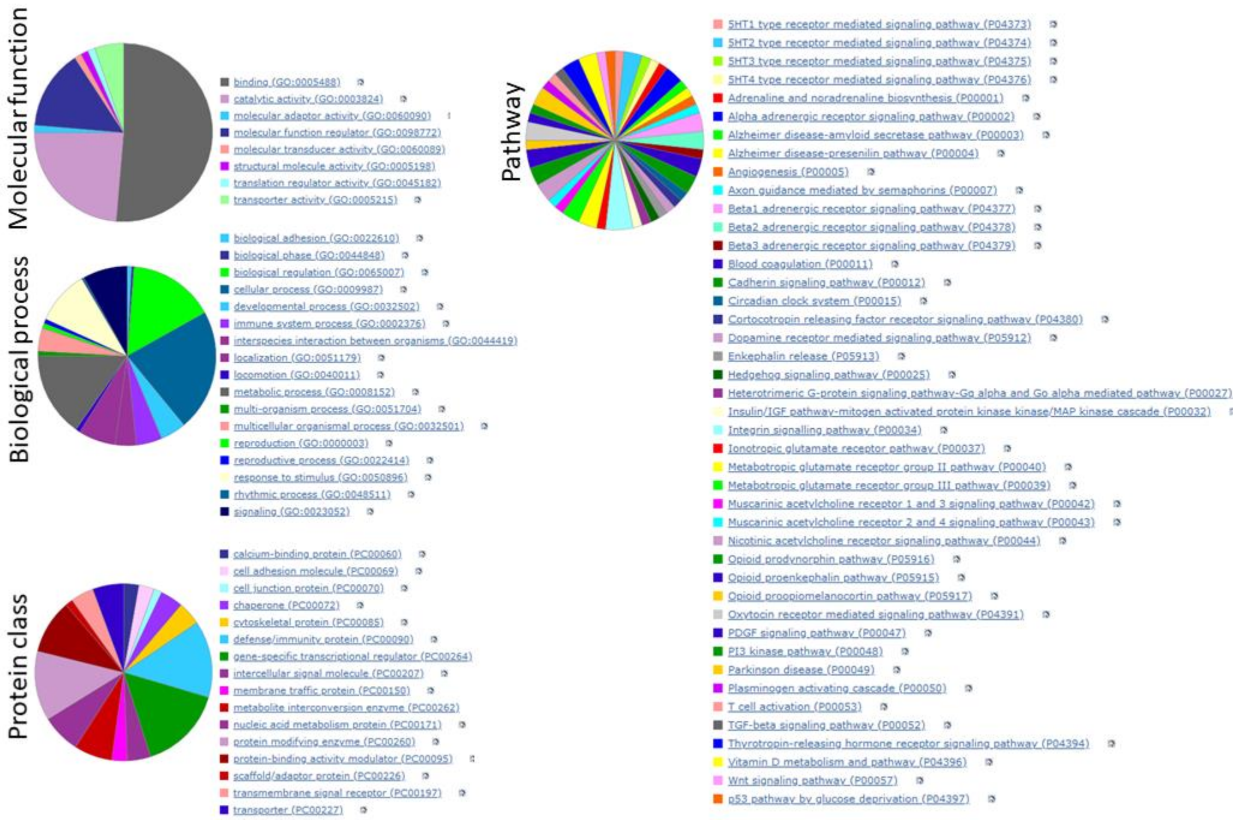

Figure S69: Functional classification of upregulated proteins in GC HPp *vs*. V.

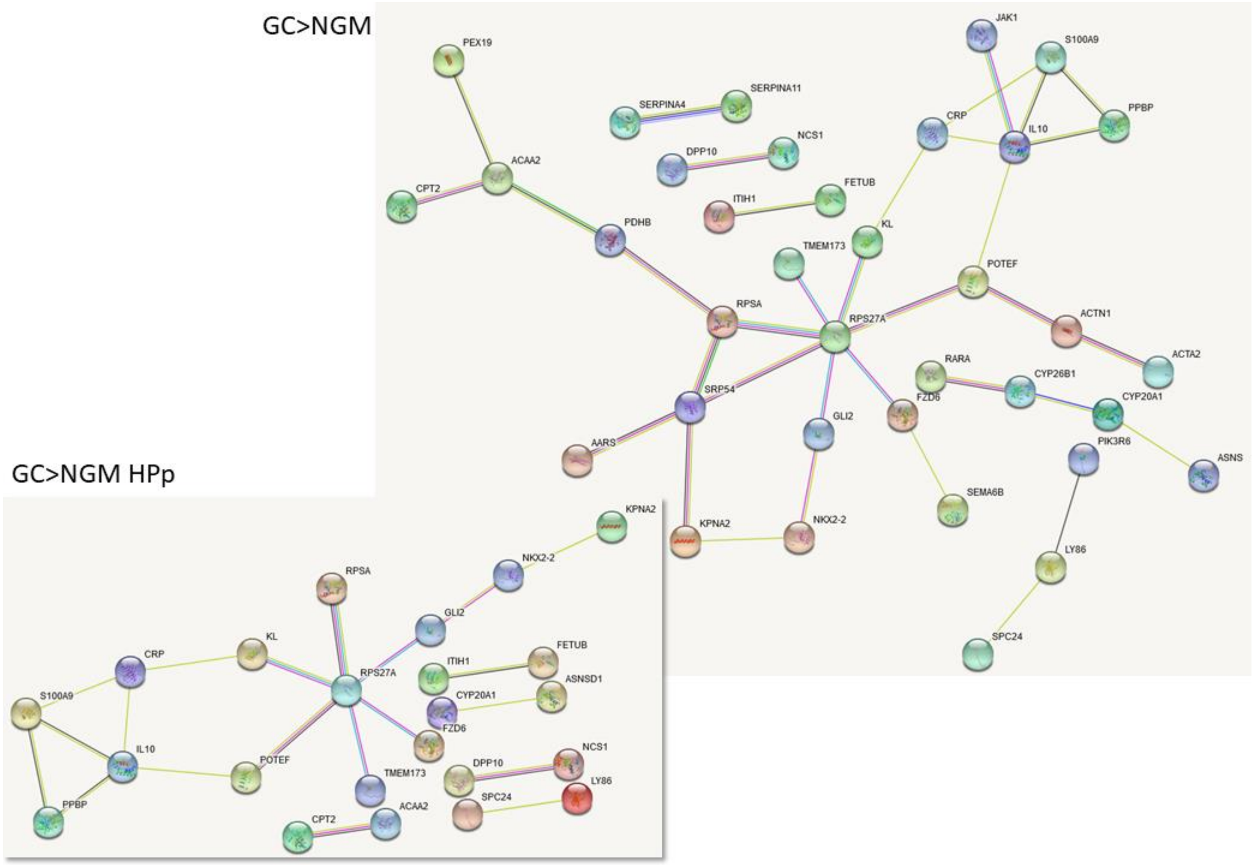

Figure S70: String network of shortlisted proteins upregulated in GC and GC HPp *vs*. NGM.

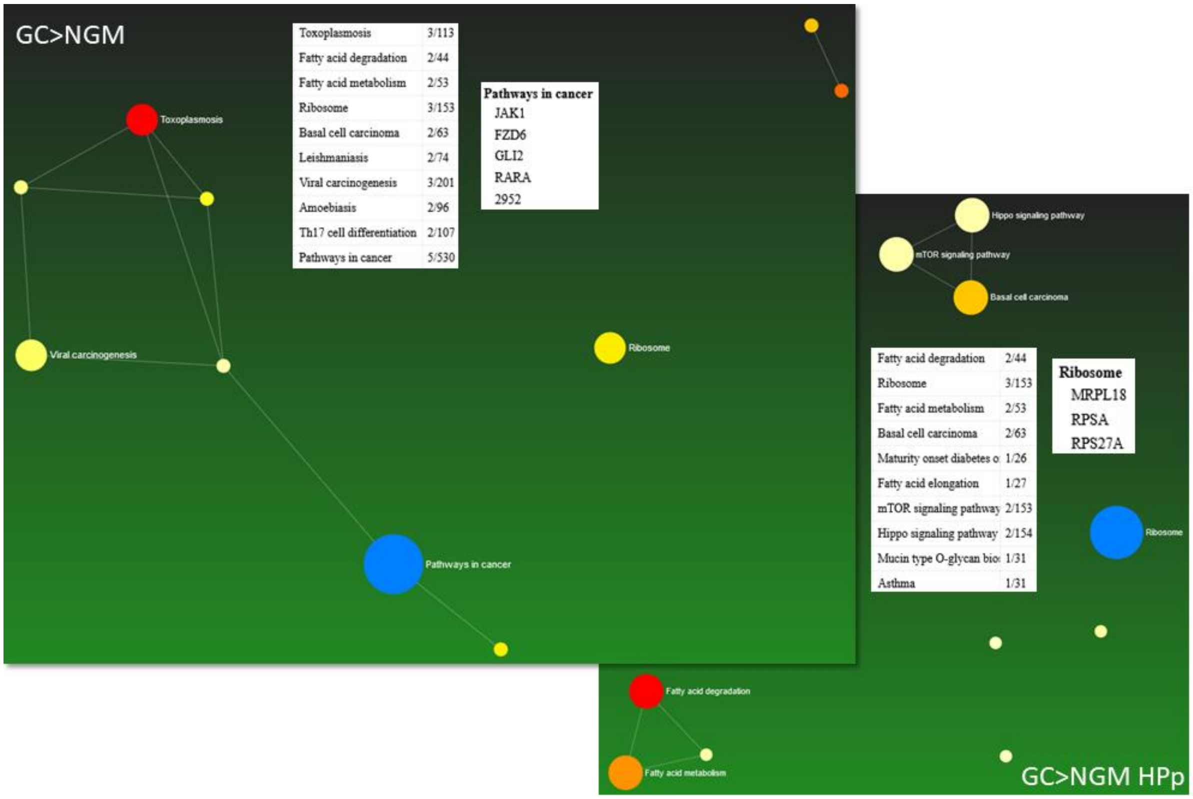

Figure S71: ORA network of upregulated shortlisted proteins in GC and GC HPp *vs*. NGM.

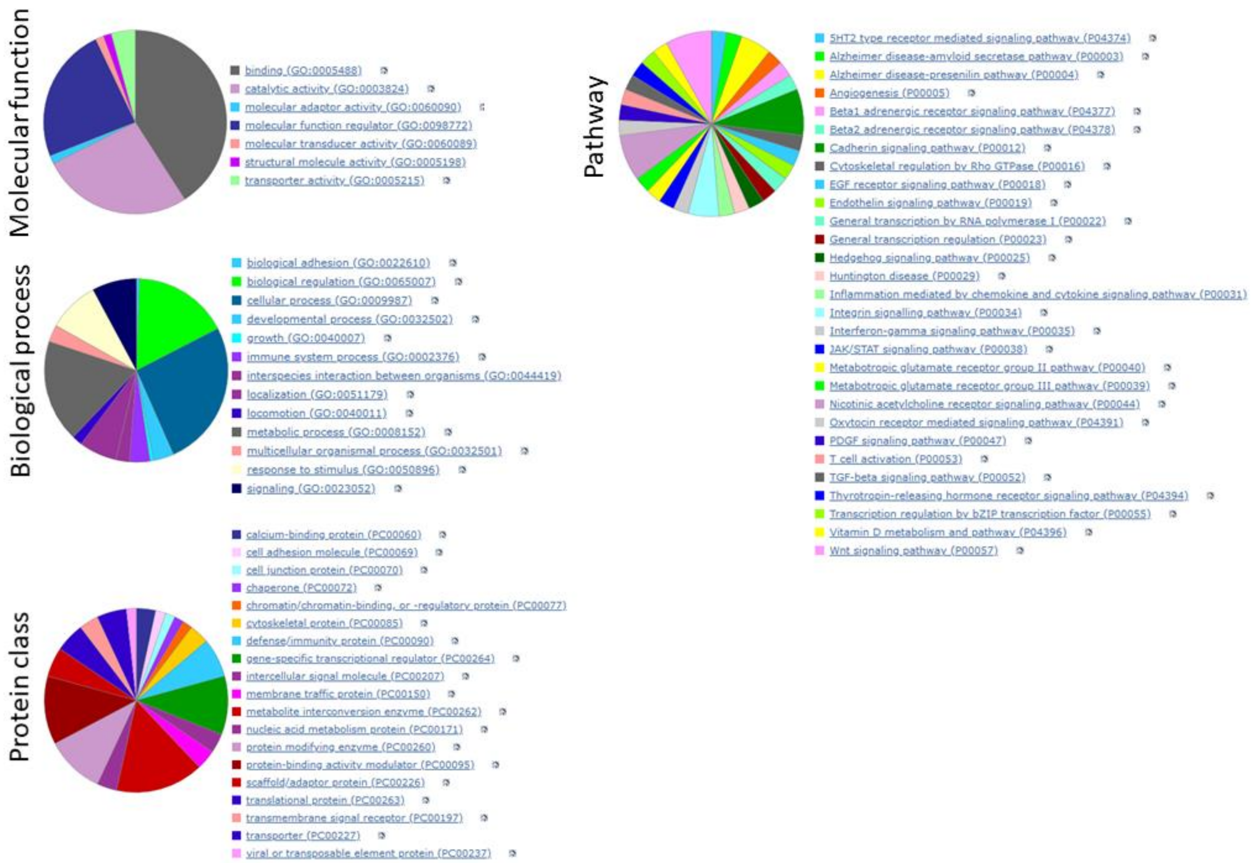

Figure S72: Functional classification of upregulated proteins in GC *vs.* NGM.

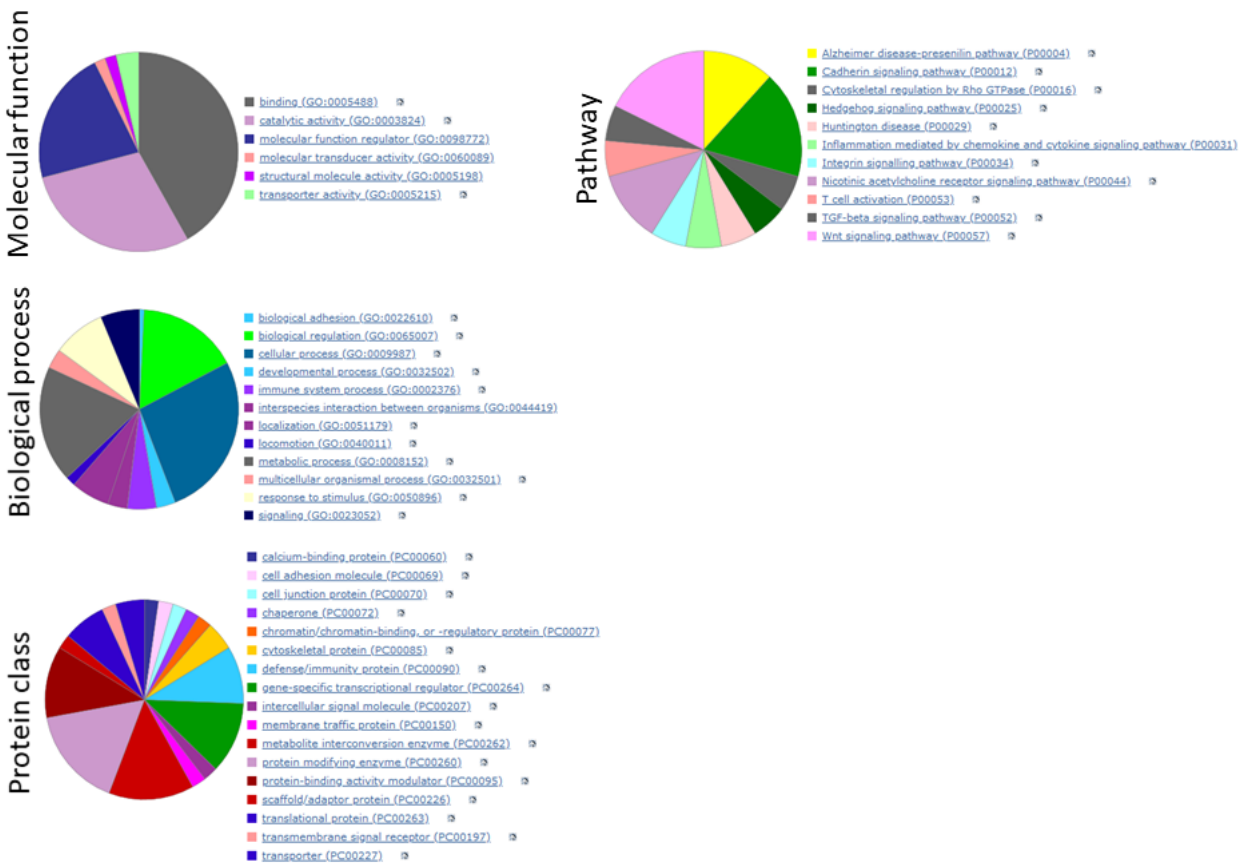

Figure S73: Functional classification of upregulated proteins in GC HPp vs. NGM.

Table S4: Shortlisted proteins only upregulated in GC vs. V and NGM, HPp/m (Supplementary file "serum_analysis").

Accession	Description	Name
P19827	Inter-alpha-trypsin inhibitor heavy chain H1	ITIH1
A5A3E0	POTE ankyrin domain family member F	POTEF
O60353	Frizzled-6	FZD6
P29622	Kallistatin	SERPINA4
Q8N608	Inactive dipeptidyl peptidase 10	DPP10
P02741	C-reactive protein	CRP
P10070	Zinc finger protein GLI2	GLI2
P04433	Immunoglobulin kappa variable 3-11	IGKV3-11
Q68E01	Integrator complex subunit 3	INTS3
O14910	Protein lin-7 homolog A	LIN7A
P68106	Peptidyl-prolyl cis-trans isomerase FKBP1B	FKBP1B
Q53QV2	Protein LBH	LBH
O95096	Homeobox protein Nkx-2.2	NKX2-2
P22301	Interleukin-10	IL10
P62166	Neuronal calcium sensor 1	NCS1
Q86SJ2	Amphoterin-induced protein 2	AMIGO2
A2A3L6	Tetratricopeptide repeat protein 24	TTC24
P0DTU4	T cell receptor beta chain MC.7.G5	TRB
P60606	Cortexin-1	CTXN1
Q5TAB7	Protein ripply2	RIPPLY2

Table S5: GC-specific serum proteins in comparison to U and gastritis extracted by Venn analysis (Supplementary file "serum_analysis"). Proteins detected also in HPp sera were printed in bold; when they were seen only in HPp serum, names were italicized.

**Accession**	**Description**	**Name**
P19827	Inter-alpha-trypsin inhibitor heavy chain H1	**ITIH1**
A5A3E0	POTE ankyrin domain family member F	**POTEF**
O60353	Frizzled-6	**FZD6**
P29622	Kallistatin	**SERPINA4**
Q02952	A-kinase anchor protein 12	**AKAP12**
Q8N608	Inactive dipeptidyl peptidase 10	**DPP10**
P62736	Actin_ aortic smooth muscle	**ACTA2**
Q6UW02	Cytochrome P450 20A1	**CYP20A1**
Q53QV2	Protein LBH	**LBH**
Q86SJ2	Amphoterin-induced protein 2	**AMIGO2**
P22301	Interleukin-10	**IL10**
P55345	Protein arginine N-methyltransferase 2	**PRMT2**
P61106	Ras-related protein Rab-14	**RAB14**
P60606	Cortexin-1	**CTXN1**
A2A3L6	Tetratricopeptide repeat protein 24	**TTC24**
Q02641	Voltage-dependent L-type calcium channel subunit beta-1	CACNB1
P33763	Protein S100-A5	S100A5
P61011	Signal recognition particle 54 kDa protein	SRP54
Q9BUN5	Coiled-coil domain-containing protein 28B	CCDC28B
Q16650	T-box brain protein 1	TBR1
P0DTU4	T cell receptor beta chain MC.7.G5	TRB
P02741	C-reactive protein	***CRP***
Q86T90	Protein hinderin	***KIAA1328***
O43150	Arf-GAP with SH3 domain_ ANK repeat and PH domain-containing protein 2	***ASAP2***
Q8ND90	Paraneoplastic antigen Ma1	***PNMA1***
P06702	Protein S100-A9	***S100A9***
Q9H521	Putative uncharacterized protein LOC645739	***LOC645739***
P49588	Alanine--tRNA ligase_ cytoplasmic	***AARS1***
P42025	Beta-centractin	***ACTR1B***
O43692	Peptidase inhibitor 15	***PI15***

Protein marker information

25preGC-P

We placed peptidase inhibitor 15 (PI15) in our 25preGC panel, which has been suggested as a novel blood diagnostic marker for cholangiocarcinoma [85]. Also, in 25preGC-P, there were with 2-3 fold higher expression in 1st stage GC, beside members of the autoantioxidant system, integrin-β (ITGB6), which enhances proliferation, survival, and motility in GC cells [86], as well as cytochrome P450 26B1 (CYP26B1), which was found with enhanced expression in GC before [87]. Menin (MEN1) was included; it promotes the Wnt signaling pathway in pancreatic endocrine cells [88]. Peroxisomal biogenesis factor 19 (PEX19) is known to dampen the p19ARF-p53-p21WAF1 tumor suppressor pathway [89] and the gene of putative cancer susceptibility gene HEPN1 protein is frequently down-regulated in hepatocellular carcinoma; it suppresses cell growth and induces apoptosis in HepG2 cells [90]. R3H domain-containing protein 1 (R3HDM1), patatin-like phospholipase domain-containing protein 4 (PNPLA4) and Ras-related GTP-binding protein B (RRAGB) are also associated with tumor progression and metastasis [91-93]. Four more enzymes were part of the 25preGC set: phosphoinositide 3-kinase regulatory subunit 6 (PIK3R6) with the knowledge that the activation of the phosphatidylinositol 3-kinase/protein kinase B/mammalian target of rapamycin (PI3K/Akt/mTOR) signaling pathway is critical in cancer patients as well as in laboratory cancer models [94]. β-galactosidase α-2_6-sialyltransferase 2 (ST6GAL2) is associated with improved patient survival in breast cancer [95]. The carnitine system (carnitine O-palmitoyltransferase 2, mitochondrial (CPT2)) and fatty acid oxidation have long been associated with cancer metabolic plasticity [96,97]. Molybdopterin synthase catalytic subunit (MOCS2) is prognostic in renal cancer [98]. In addition, two ribosomal proteins were included (39S ribosomal protein L18, mitochondrial (MRPL18), 40S ribosomal protein SA (RPSA)). An “oncogenic ribosome” which promotes tumor progression has been proposed [99], and mitochondrial species such as MRPL17 seem to be of particular relevance in GC [100]. RPSA mutation/deletion was published for chronic lymphoblastic leukemia [101].

A number of investigations reported the expression of immunoglobulins (Igs) in many human tumor tissues and cells with tumor-derived Igs displaying functions, which are different from classical Igs produced by B lymphocytes and plasma cells [102]. We have four Igs on the panel (IGHV1-8, IGKV3-11, IGHV4-38-2, IGKV1-13). IGKV3 proteins were reported as candidate vaccines for κ-light chain–restricted B-cell non-Hodgkin lymphomas [103], the IGHV4 family for use in *Chlamydophila psittaci*-negative ocular adnexal extranodal marginal zone lymphomas [104], and IGKV1-13 recombinations were detected in solid tumor specimen exome files [105]. We also found G patch domain and ankyrin repeat-containing protein 1 (GPANK1), which was not prognostic in stomach cancer according to the Human Protein Atlas [106] and our own measurements in gastric biopsies, but it was in serum. GPANK1 polymorphism was of interest in SNP screening of the central major histocompatibility complex for candidate susceptibility genes for HIV-related Kaposi's sarcoma [107].

10GC-P

Fold changes of 10GC-P proteins *vs.* NGM were moderate (2-3.4 fold). Most intensity showed inter-α-trypsin inhibitor heavy chain H1 (ITIH1), which was identified as a novel prognostic indicator for hepatocellular carcinoma recently [108], albeit the observation of frequent expression loss of ITIH genes in multiple human solid tumors [109]. It was followed by the Wnt-pathway regulator FZD6 [62] and inactive dipeptidyl peptidase 10 (DPP10), which has been investigated in breast cancer [110]. Kallistatin (SERPINA4) was proposed as biomarker not only for cancer, but also for hypertension and organ injury [63,111]. A-kinase anchor protein 12 (AKAP12) was downregulated in human hepatocellular carcinoma [112]. Protein S100A9 promotes invasion and migration in gastric cancer cells [46] and was selected along with S100A8 as potential biomarker for renal cell carcinoma in the early stages [113]. Information on POTE ankyrin domain family member F (POTEF), number seven on our panel, was not as readily available. From prostate cancer cells it is known that androgen-induced lncRNA POTEF-AS1 regulates the apoptosis-related pathway to facilitate cell survival [114]. POTEE drives colorectal cancer development via regulating SPHK1/p65 signaling [115]. Multifunctional ion channel auxiliary subunits have also been associated with cancer [116] and pathway-level analysis providing enrichment on genomic alterations associated voltage-dependent L-type calcium channel subunit β-1 (CACNB1) with gastric adenocarcinoma [117]. Hinderin is a five-domain protein including coiled-coil motifs with no direct link to cancer [118], but, interestingly, it binds to structural maintenance of chromosomes protein 3 (SMC3). The circular RNA, which is generated from the SMC3 gene locus (circSMC3), regulates gastric cancer tumorigenesis by targeting the miR-4720-3p/TJP1 axis [119]. Last, but not least, CRP was significant in our analysis. Although it is a common inflammation marker in clinical diagnostics, abnormally elevated preoperative serum levels were observed in GC patients and were associated with progressive disease or an advanced stage, and a worse survival in another study [120].
